# Index- and Pareto-based multi-trait selection identifies dual-purpose sorghum and pearl millet cultivars for arid agro-ecosystems of Saudi Arabia

**DOI:** 10.3389/fpls.2026.1795221

**Published:** 2026-08-10

**Authors:** Ephrem Habyarimana, Abdullah Alhendi, Kakoli Ghosh, Niranjan Ravindra Thakur, Ashok Kumar Are, Rajan Sharma, Jagdish Jaba, Shashi Kumar Gupta, Henry F. Ojulong, Riyazaddin Mohammed, Falalou Hamidou, Ahmed Abdelmahoud, Anitha Raman, Rashed Almarri

**Affiliations:** 1 International Crops Research Institute for the Semi-Arid Tropics, Patancheru, India; 2 Food and Agriculture Organization of the United Nations, Riyadh, Saudi Arabia; 3 International Crops Research Institute for the Semi-Arid Tropics, ICRAF House, Gigiri, Nairobi, Kenya; 4 International Crops Research Institute for the Semi-Arid Tropics, Niamey, Niger

**Keywords:** arid agro-ecosystems, dual-purpose crops, genotype-by-environment interaction, multi-trait selection, Pareto optimization, pearl millet, sorghum

## Abstract

**Background:**

Rainfed cereal production in southwestern Saudi Arabia depends largely on farmer-saved landraces, creating an important opportunity to strengthen grain and fodder productivity in mixed crop-livestock systems exposed to heat and variable rainfall. This study evaluated introduced dual-purpose sorghum and pearl millet germplasm under contrasting arid, supplemental-irrigated rainfed environments, and applied complementary quantitative approaches to identify high-performing, stable candidates that balance grain yield and green-fodder production while accounting for genotype × environment interaction (G×E).

**Methods:**

Twenty-seven sorghum and 15 pearl millet entries were evaluated in alpha-lattice trials at the contrasting Jazan lowland and Abha highland sites across four site × season/sowing-window environments. Linear mixed models quantified environment, genotype, and G×E effects and estimated entry-mean reliability. Stability and adaptation patterns were evaluated using the weighted average of absolute scores from BLUPs (WAASB) and GGE biplots. Dual-purpose performance was assessed using a standardized 50:50 grain-yield-fresh-biomass index as a transparent baseline in the absence of validated local economic or farmer-preference weights. Pareto-frontier analysis was used to identify non-dominated genotypes representing efficient grain-green-fodder trade-offs.

**Results:**

The trials revealed substantial genetic variation and contrasting genotype responses across environments, demonstrating considerable scope for improving grain and green-fodder productivity. Selection reliability was very high for sorghum fresh biomass and pearl millet grain yield, and high for pearl millet fresh biomass, providing a strong basis for prioritizing selection candidates within the target production environments. Sorghum grain yield showed lower reliability because only two grain-evaluable environments were available and G×E variance was large, highlighting a clear priority for expanded grain testing. SSV 74, WM 89/90#1615, and 89WM 5003 achieved the highest sorghum DualIndex values, while ICSV 15021 combined the highest mean fresh biomass with the lowest WAASB, identifying it as a particularly promising forage-oriented candidate. In pearl millet, LCICMV-1 (SOSAT-C-88) and ICMV 91450 displayed favorable grain-green-fodder profiles, ICMV 221 recorded the highest mean grain yield, and LCICMV-4 (Jirani) showed promising grain-yield performance under Jazan-associated conditions.

**Conclusions:**

The study identified a valuable portfolio of sorghum and pearl millet candidates addressing complementary production objectives, including high green-fodder productivity, balanced grain-fodder performance, high grain yield, and targeted environmental adaptation. By integrating mixed-model reliability, stability analysis, a transparent grain-green-fodder index, and Pareto-frontier assessment, the study provides a rigorous and practical framework for converting early multi-environment data into evidence-based advancement decisions. The identified genotypes provide a strong foundation for expanded multi-location and participatory on-farm validation, standardized fodder-quality assessment, and staged seed-system development. Collectively, these findings advance the development of productive and resilient dual-purpose cereal options capable of strengthening grain and fodder security in southwestern Saudi Arabia and comparable arid mixed crop-livestock systems.

## 1 Introduction

**Figure 1:**
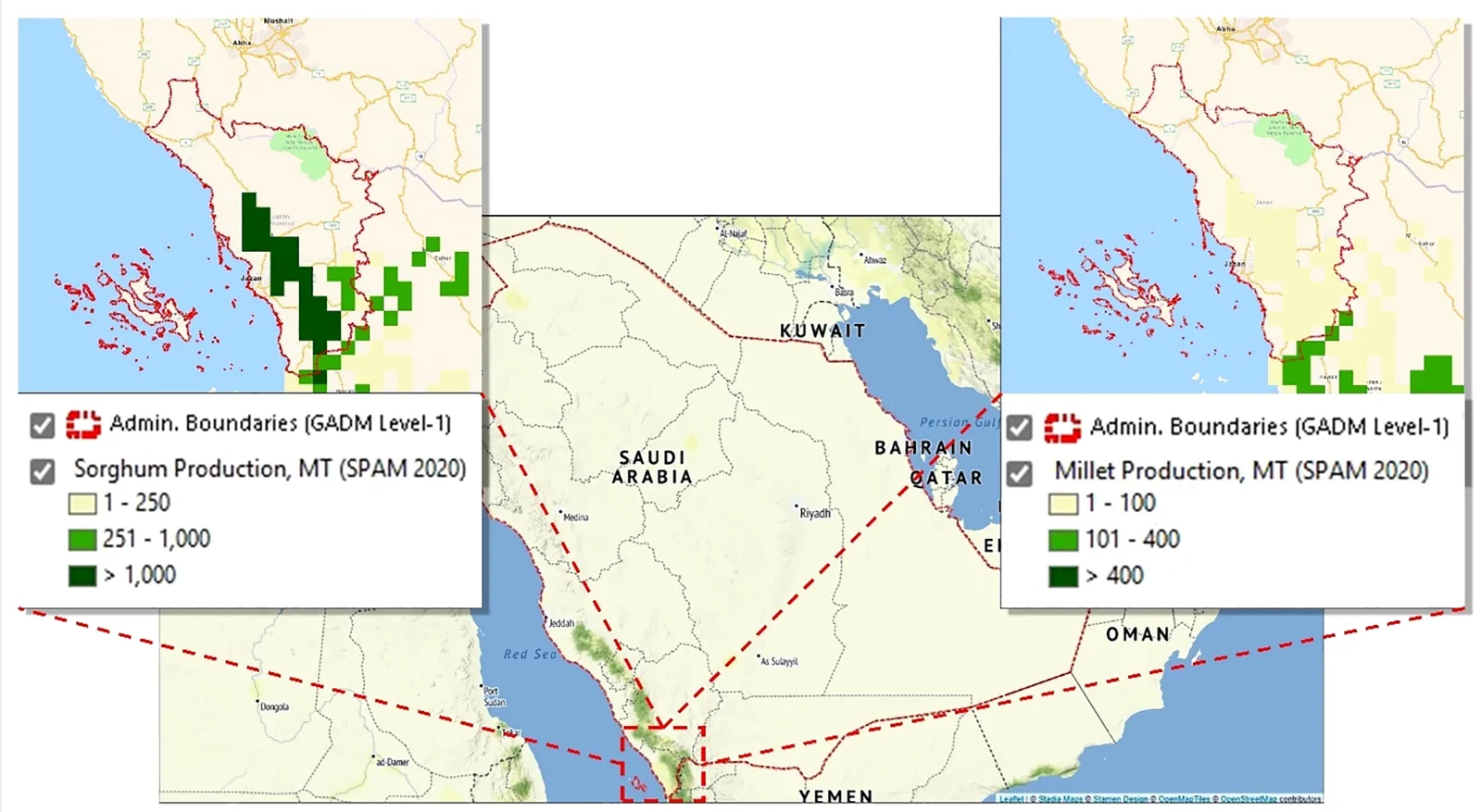
Regional production of sorghum and pearl millet in Saudi Arabia, highlighting the dominance of the Jazan region. Maps are based on data from the USDA Foreign Agricultural Service and the International Production Assessment Division (IPAD).

Sorghum and pearl millet are central to the resilience of rainfed agriculture in southwestern Saudi Arabia (Farrelly and Mitchell, 2024; Agroberichten Buitenland, 2018; Argaam, 2018), where they support household food security and livestock production through grain, stover, and green fodder in mixed crop-livestock systems (**Figure 1**; Food and Agriculture Organization of the United Nations, 2024; Chaturvedi et al., 2022). According to national agricultural statistics, sorghum, including white sorghum, occupied 71,228 ha and produced 191,882 t, while pearl millet occupied 7,061 ha and produced 13,235 t across the Kingdom (General Authority for Statistics, 2023; Saudi Agricultural and Livestock Investment Company, 2023). Their capacity to produce under high temperatures, variable rainfall, and relatively marginal growing conditions makes both crops strategically important for climate-resilient agriculture, particularly where more water-demanding cereals are less dependable (Chaturvedi et al., 2023; Pixley et al., 2023; Varshney et al., 2017).

In mixed crop-livestock systems, the value of sorghum and pearl millet extends beyond grain production. Productive cultivars can contribute simultaneously to household food supply and livestock feeding by providing grain, green fodder, and crop residues. Balanced grain-fodder performance is therefore a practical breeding and deployment objective that directly addresses the needs of farmers managing both crop and livestock enterprises. Recognizing this opportunity, recent FAO-led rainfed cereal initiatives have prioritized Jazan and Asir as strategic agro-ecologies for germplasm introduction, evaluation, participatory validation, and seed-system development in sorghum, pearl millet, and sesame (Food and Agriculture Organization of the United Nations, 2024). The continued importance of farmer-maintained landraces and informal seed exchange, particularly in Jazan, also provides a strong rationale for complementing locally adapted materials with carefully evaluated improved germplasm. Such an approach can broaden the available cultivar portfolio while preserving the adaptation, grain characteristics, fodder value, and other attributes valued by local farming communities.

A major opportunity in evaluating improved germplasm for these production systems is to identify genotypes that combine high productivity with dependable performance across variable growing conditions. Genotype × environment interaction (G×E) can modify the relative performance of cultivars across locations, years, seasons, and sowing windows (Malosetti et al., 2013; Isik et al., 2017; Yan, 2002). Under arid and semi-arid conditions, the timing and intensity of heat and water limitation can have different effects on vegetative growth, flowering, grain formation, and biomass accumulation, thereby generating meaningful differences in genotype responses among environments (Malosetti et al., 2013; Yan, 2002; Pixley et al., 2023; Varshney et al., 2019). Characterizing these responses creates an opportunity to identify both broadly useful cultivars and materials suited to specific production conditions.

Modern dryland breeding, therefore, increasingly emphasizes cultivars that integrate stress adaptation, grain productivity, biomass production, and fodder value within clearly defined target production systems (Pixley et al., 2023; Chaturvedi et al., 2023; Varshney et al., 2019). Multi-environment trials provide the evidence needed to quantify G×E, estimate selection reliability, distinguish repeatable genotype differences from environmental variation, and identify promising adaptation patterns. Mixed-model approaches further strengthen this process by providing adjusted genotype estimates, variance components, entry-mean reliability, and predicted G×E effects while accounting for experimental design and field heterogeneity (Malosetti et al., 2013; Isik et al., 2017; Olivoto et al., 2019; Olivoto and Lúcio, 2020). When combined with appropriate stability analyses, these methods can convert multi-environment data into practical information for candidate advancement and the design of subsequent validation trials (Ma et al., 2024).

Southwestern Saudi Arabia presents a particularly valuable setting for developing this evidence base. The region combines contrasting lowland and highland environments, variable rainfall and temperature regimes, different sowing opportunities, strong demand for both grain and livestock feed, and growing institutional support for rainfed cereal improvement. Integrated evaluations that combine replicated multi-environment testing, mixed-model estimates of trait-specific selection reliability, stability analysis, and explicit assessment of grain-fodder trade-offs remain scarce. Addressing this opportunity can strengthen the transition from germplasm introduction to evidence-based candidate prioritization, participatory validation, and, ultimately, more effective seed delivery. It can also complement the valuable adaptation represented by farmer-maintained landraces with improved materials offering additional productivity, stability, or end-use advantages (Food and Agriculture Organization of the United Nations, 2024; U.S. Department of Agriculture, Foreign Agricultural Service, International Production Assessment Division, 2024; International Crops Research Institute for the Semi-Arid Tropics, 2023, 2025).

Selection for dual-purpose performance is inherently multi-trait because grain yield and fodder production are not necessarily maximized by the same genotype. Selection based on grain alone may overlook genotypes with substantial fodder value, while selection based only on biomass may favor materials that do not adequately contribute to household grain supply. A balanced selection strategy is therefore needed to identify genotypes that combine both outputs or represent efficient alternatives along the grain-fodder continuum (Moeinizade et al., 2020; Joukhadar et al., 2024).

Fresh biomass was selected as the primary fodder-related trait in this study because the local target product profile prioritized green-fodder production and because fresh biomass provides a direct and readily interpretable measure of the material available for immediate livestock feeding. Fresh biomass is also operationally useful in large-scale field evaluation because it can be measured rapidly across many plots without the extensive sample processing, drying capacity, and time required for direct determination of dry matter yield. Importantly, fresh biomass is generally strongly associated with dry biomass at phenotypic and genetic levels in sorghum, supporting its use as a practical high-throughput proxy for overall biomass productivity when standardized oven-drying of all harvested samples is not feasible (Oliveira et al., 2020). This relationship enables breeding programs to differentiate efficiently among genotypes for biomass production while evaluating large populations across multiple environments.

Fresh biomass is also directly relevant to the intended deployment context because green fodder is an immediate and visible output for farmers and livestock producers. Nevertheless, genotypic variation in stem moisture, juiciness, and dry-matter percentage can influence the relationship between fresh and dry biomass. Future evaluations can therefore build on fresh-biomass screening by measuring representative samples dried to constant weight and by incorporating dry-matter percentage, digestibility, crude protein, fiber fractions, lignin, palatability, lodging resistance, and other attributes related to fodder quality and recoverable yield. In this way, fresh biomass provides an efficient first-stage selection trait that can be complemented by increasingly detailed feed-value assessment as promising candidates advance through the validation pipeline.

Multi-trait selection indices offer a practical method for integrating grain and fodder performance into a common decision criterion (Smith, 1936; Hazel, 1943; Moeinizade et al., 2020; Covarrubias-Pazaran, 2021). Building on the foundational Smith-Hazel framework, desired-gain and standardized-index approaches can accommodate traits that differ in units, scale, variance, correlation, and breeding priority (Smith, 1936; Hazel, 1943; Covarrubias-Pazaran, 2021; Joukhadar et al., 2024). In the present study, an equal-weight standardized index reflects the formal dual-purpose target product profile in which grain and green fodder were both identified as priority outputs. In the absence of validated Saudi Arabia-specific economic weights or farmer-preference scores, equal weighting provides a transparent and reproducible baseline for balanced selection.

This baseline can be refined as additional information becomes available. Farmers and livestock users may assign different values to grain and fodder depending on household food requirements, livestock ownership, seasonal feed shortages, market prices, labor availability, and production risk. Participatory evaluation can therefore generate context-specific weights and potentially define separate product profiles for grain-focused, forage-focused, and balanced dual-purpose cultivars. This flexibility enhances the practical value of selection indices by allowing the framework to evolve with stakeholder priorities rather than imposing a fixed valuation of the two outputs.

Pareto-frontier analysis provides a valuable complement to the weighted index. Instead of identifying a single optimum based on predetermined weights, Pareto analysis identifies non-dominated genotypes for which improvement in one evaluated trait would require a reduction in the other (Pareto, 1896; Miettinen, 1999; Deb, 2001; Deb et al., 2002). This approach preserves genotypes representing different but efficient grain-fodder profiles and helps prevent potentially valuable materials from being excluded simply because they do not maximize an equal-weight index. Pareto-front membership indicates an efficient trade-off for the evaluated traits rather than overall biological or statistical superiority. Joint interpretation of the DualIndex and Pareto frontier, therefore, provides both a balanced ranking and a broader portfolio of efficient candidate options.

Applications in forage sorghum and wheat demonstrate the value of multi-trait stability and desired-gain approaches when several breeding objectives must be considered and validated economic weights are unavailable (Behera et al., 2024a; Joukhadar et al., 2024). These studies provide a strong conceptual basis for combining multi-environment reliability estimates, stability metrics, standardized multi-trait indexing, and Pareto analysis (Moeinizade et al., 2020; Shrote and Thompson, 2024). The present study extends this practical application to introduced dual-purpose sorghum and pearl millet germplasm evaluated under the contrasting production conditions of southwestern Saudi Arabia. By connecting regional production priorities with quantitative selection tools, the study provides an actionable pathway from germplasm evaluation to targeted multi-location and participatory validation.

Accordingly, the objectives of this study were to: (i) quantify genotype, environment, and G×E effects for grain yield and fresh biomass in introduced sorghum and pearl millet germplasm; (ii) estimate trait-specific selection reliability and assess genotype stability and adaptation within the tested environments; (iii) identify promising grain-green-fodder candidates using an equal-weight standardized DualIndex and complementary Pareto-frontier analysis; and (iv) define candidate portfolios and evidence priorities for expanded multi-environment and participatory on-farm validation in southwestern Saudi Arabia. By integrating mixed-model inference, WAASB and GGE analyses, standardized index selection, and Pareto-frontier assessment, the study provides a transparent decision-support framework for translating early multi-environment evidence into breeding and validation priorities. The resulting candidate portfolio and analytical approach can support the progressive development of productive, resilient, and farmer-relevant sorghum and pearl millet options for southwestern Saudi Arabia and comparable arid mixed crop-livestock systems.

## 2 Materials and methods

### 2.1 Experimental sites

This study was conducted as part of an ongoing partnership between FAO-KSA and ICRISAT aimed at introducing and validating high-performing sorghum and pearl millet varieties for Saudi Arabian rainfed production with supplemental irrigation (rainfed-supplemented) production systems. Improved genotypes developed by ICRISAT breeding programs in Asia and Africa were introduced into Saudi Arabia for multi-environment evaluation under managed dryland conditions.

Trials were established at two contrasting agro-ecological sites: the Jazan Agricultural Research Centre (JARC; 17.01262°N, 42.855259°E; lowland; 43 m a.s.l.) and the Seed Multiplication Station in Abha (18.249773°N, 42.512345°E; highland; 2,200 m a.s.l.). For this study, an environment was defined as a unique site × season/sowing-window combination, yielding four distinct environments for multi-environment trial (MET) analysis: Jazan-September 2023, Jazan-October 2023, Jazan-September 2024, and Abha-September 2024 (**Table 1**). This definition was adopted because genotype performance in these dryland systems is jointly influenced by location, seasonal conditions, and sowing window. Jazan contributed three environments across two years and sowing windows, whereas Abha contributed one contrasting highland environment because of operational constraints. This imbalance limited the separate estimation of location, year, and sowing-window effects but did not prevent mixed-model estimation of genotype performance and G×E within the tested environments, consistent with established analysis of unbalanced multi-environment trials (Barreto et al., 2024).

**Table 1:** Summary of test environments used in the multi-environment trials.

Env.	Location	Agro-ecology/Elevation	Cropping year/season	Sowing period (day.month.year)	Seasonal rainfall (mm)	Temperature range (°C)
E1	Jazan Agricultural Research Centre	Lowland; 43 m a.s.l.	2023–2024	18.09.2023	37	25.0–35.5
E2	Jazan Agricultural Research Centre	Lowland; 43 m a.s.l.	2023–2024	17.10.2023	37	25.0–35.5
E3	Jazan Agricultural Research Centre	Lowland; 43 m a.s.l.	2024–2025	19.09.2024	69	25.0–35.5
E4	Seed Multiplication Station, Abha	Highland; 2,200 m a.s.l.	2024	09.05.2024	77.8	18.0–31.5

The trial structure enabled estimation of genotype, environment, and G×E effects across the tested conditions. Stability and adaptation patterns were interpreted within these environments and provide a basis for validation across additional years, locations, sowing windows, and farmer-managed conditions.

#### 2.1.1 Site characterization, soil properties, and seasonal weather

Abha (highland site; 2,200 m a.s.l.). Field trials were conducted during the 2024 summer cropping season in a mountainous highland environment. Composite soil samples collected across the experimental area comprised 57% sand, 18% silt, and 25% clay, corresponding to a sandy clay loam texture. Soil pH was 7.93 and electrical conductivity of the saturation extract (ECe) was 1.27 dS m^-1^. Extractable nutrient concentrations were N = 25 ppm, P = 7.8 ppm, and K = 220 ppm. During the growing season, minimum and maximum temperatures ranged from 18.0°C to 31.5°C, mean relative humidity was 42.3%, and cumulative rainfall was 77.8 mm. Supplemental irrigation totaled 231.2 mm for sorghum and 201.7 mm for pearl millet.

Jazan (lowland site; 43 m a.s.l.). Trials were conducted during the 2023-2024 and 2024-2025 cropping seasons. Composite soil samples indicated 53.61% sand, 21.19% silt, and 25.20% clay (sandy clay loam texture). Soil pH was 8.12 and ECe was 0.97 dS m^-1^, with measured nutrient concentrations of N = 20 ppm, P = 12.7 ppm, and K = 195 ppm. Across the two seasons, minimum and maximum temperatures ranged from 25.0°C to 35.5°C, mean relative humidity was 66.3%, and rainfall was 37 mm in 2023-2024 and 69 mm in 2024-2025. Supplemental irrigation for sorghum totaled 285 mm and 277 mm in the respective seasons, while pearl millet received 245.2 mm and 233.8 mm.

At both locations, supplemental irrigation was triggered by persistent crop-specific water-stress symptoms, particularly sustained leaf wilting and loss of leaf turgor, considered together with prevailing weather and field-moisture conditions (Mitchell et al., 2025; Vadez et al., 2024). The supplemental irrigation was provided through drip lines to support crop establishment and prevent severe moisture stress. Irrigation commenced after sowing and continued until crop maturity. Water delivery was maintained uniformly within each trial through routine inspection of drip lines and emitters and correction of leaks or blockages.

At both sites, soil particle-size distribution was determined by the hydrometer method, pH and electrical conductivity by the soil paste method, nitrogen by the Kjeldahl method, phosphorus by the ascorbic acid method, and potassium by flame photometry (Bouyoucos, 1962; Richards, 1954; Jones, 1991; Watanabe and Olsen, 1965; Doll and Lucas, 1973). The low ECe values at both sites indicated non-saline soil conditions, while the slightly alkaline pH values were considered acceptable for sorghum and pearl millet growth under the trial management conditions (Byrt et al., 2023).

Combined rainfall and supplemental irrigation supported crop establishment and growth across the test environments. Rainfall and temperature were used to characterize environmental contrasts and interpret genotype performance but were not included as covariates in the G×E model. Given the four-environment dataset and the confounding of weather with location, season or sowing window, altitude, and management, fitting these variables separately could have resulted in over-parameterization and unstable estimates (Van Eeuwijk et al., 2016; Piepho and Williams, 2024).

### 2.2 Experimental design and plant materials

All trials were established in an alpha-lattice design with three replications. Each plot comprised two 5-m rows, spaced 65 cm between rows and 30 cm between plants. Recommended agronomic practices, including drip irrigation, fertilization, weed control, and pest management, were applied uniformly within each environment. Protective netting was installed at ARC-Jazan to minimize bird-related grain losses. These well-managed on-station conditions supported reliable genotype comparisons, while subsequent farmer-managed trials will validate candidate performance under representative production practices.

A total of 42 entries were evaluated across two crops: 27 sorghum entries and 15 pearl millet entries. The sorghum panel consisted of 24 candidate entries developed by ICRISAT (15 from ICRISAT-India and 9 from ICRISAT-Kenya) together with three local benchmark checks provided by FAO-KSA (Jazan Ahmar, Jazan Abyad, and Jazan Shahla). The pearl millet panel comprised 12 candidate entries from ICRISAT (5 from ICRISAT-India, 3 from ICRISAT-Kenya, and 4 from ICRISAT-Niger) and three local benchmark checks (Baladi, Ubali, and Yamani). Entry details and provenances are presented in **Tables 2**, **3**.

**Table 2:** Sorghum genotypes evaluated in the experiments, with their respective provenances.

S no.	Genotype	Provenance	Candidate/Check
1	ICSR 14001	ICRISAT, India	Candidate
2	ICSR 93034	ICRISAT, India	Candidate
3	ICSR 93036	ICRISAT, India	Candidate
4	ICSV 112 (CSV 13)	ICRISAT, India	Candidate
5	ICSV 12019	ICRISAT, India	Candidate
6	ICSV 15006	ICRISAT, India	Candidate
7	ICSV 15014	ICRISAT, India	Candidate
8	ICSV 15021	ICRISAT, India	Candidate
9	ICSV 17371	ICRISAT, India	Candidate
10	ICSV 18002	ICRISAT, India	Candidate
11	ICSV 25333	ICRISAT, India	Candidate
12	ICSV 745	ICRISAT, India	Candidate
13	SSG 59-3	ICRISAT, India	Candidate
14	SSV 74	ICRISAT, India	Candidate
15	SSV 84	ICRISAT, India	Candidate
16	89WM 5003	ICRISAT, Kenya	Candidate
17	89WM 5056	ICRISAT, Kenya	Candidate
18	Gadam	ICRISAT, Kenya	Candidate
19	IESV 91104DL	ICRISAT, Kenya	Candidate
20	IESV 92053DL	ICRISAT, Kenya	Candidate
21	IESV 92089DL	ICRISAT, Kenya	Candidate
22	KAT 487	ICRISAT, Kenya	Candidate
23	S 35	ICRISAT, Kenya	Candidate
24	WM 89/90#1615	ICRISAT, Kenya	Candidate
25	Jazan Ahmar	FAO-KSA	Check 1
26	Jazan Abyad	FAO-KSA	Check 2
27	Jazan Shahla	FAO-KSA	Check 3

Candidate/Check indicates whether the entry is a local benchmark check (Check 1–3) or a test entry (Candidate).

**Table 3:** Pearl millet genotypes evaluated in the experiments, sorted by provenance.

S no.	Genotype	Provenance	Candidate/Check
1	ICMH 356	ICRISAT, India	Candidate
2	ICMV 05222	ICRISAT, India	Candidate
3	ICMV 221	ICRISAT, India	Candidate
4	ICMV1707	ICRISAT, India	Candidate
5	ICTP 8203	ICRISAT, India	Candidate
6	ICMV 221 White	ICRISAT, Kenya	Candidate
7	ICMV 91450	ICRISAT, Kenya	Candidate
8	Okashana 1	ICRISAT, Kenya	Candidate
9	ICMH 147007	ICRISAT, Niger	Candidate
10	ICMVIS 89305	ICRISAT, Niger	Candidate
11	LCICMV-1 (SOSAT-C-88)	ICRISAT, Niger	Candidate
12	LCICMV-4 (Jirani)	ICRISAT, Niger	Candidate
13	Baladi	FAO-KSA	Check 1
14	Ubali	FAO-KSA	Check 2
15	Yamani	FAO-KSA	Check 3

Candidate/Check indicates whether the entry is a local benchmark check (Check 1–3) or a test entry (Candidate).

### 2.3 Trait measurements

Phenotypic data were recorded for the principal agronomic, grain, and biomass traits required to assess dual-purpose performance. For sorghum, the following traits were measured: days to 50% flowering (DF50), plant height (PH, cm), 100-seed weight (100SW, g), grain yield (GY, kg ha^-1^), juice Brix (%), stalk fresh weight or fresh biomass (FW, kg ha^-1^), stalk dry weight or dry biomass (DW, kg ha^-1^), stalk diameter (SD, mm), internode number, and internode length (INL, cm). For pearl millet, recorded traits included DF50, panicle length (PL, cm), 1000-seed weight (1000SW, g), GY (kg ha^-1^), FW (kg ha^-1^), and DW (kg ha^-1^).

Trait recording followed standard crop descriptor protocols for sorghum and pearl millet, together with relevant DUS and agro-morphological measurement guidelines (International Board for Plant Genetic Resources and International Crops Research Institute for the Semi-Arid Tropics, 1993a, 1993b; Protection of Plant Varieties and Farmers’ Rights Authority, 2019a, 2019b). Flowering time was recorded when 50% of plants in a plot had reached anthesis; plant and panicle traits were measured on representative non-border plants at the recommended crop stage; grain yield was calculated from the harvested plot area and expressed as kg ha^-1^ at approximately 12% grain moisture; and fresh biomass was measured from harvested stalks and converted to a per-hectare basis. For sorghum Brix, juice was extracted from the middle third of representative stalks and measured with a handheld refractometer. In the 2023 Jazan trials (E1 and E2), sorghum grain weight per five plants was also recorded as a supporting grain-related trait. At physiological maturity, panicles from five representative non-border plants per plot were harvested, threshed, and weighed, with grain mass expressed as grams per five plants.

Plot-level sorghum grain yield was not recorded in E1 and E2. In these environments, grain weight from five representative plants was retained only as a supporting grain-related trait and was not extrapolated to kg ha^-1^ because it did not represent whole-plot yield. Consequently, the combined sorghum grain-yield analysis was restricted to the two environments with plot-level grain-yield data, E3 and E4.

Fresh biomass was selected as the principal fodder trait because the locally defined target product profile prioritized cultivars combining grain production with green-fodder availability. As a direct, practical, and field-relevant measure, fresh biomass enabled high-throughput evaluation of fodder productivity and, given its strong association with dry biomass in sorghum (Oliveira et al., 2020), provided a useful proxy for overall biomass yield. Although dry biomass was recorded, suitable facilities were unavailable to oven-dry samples to constant weight and determine the dry matter fraction of fresh materials (Habyarimana et al., 2018) under controlled conditions. The dry-mass measurements were therefore insufficiently standardized for reliable genotype comparisons and were excluded from the primary statistical analyses and DualIndex. Accordingly, the DualIndex represents grain and green-fodder productivity. Future validation will complement fresh-biomass measurements with oven-drying-based biomass yield, dry-matter percentage, and relevant fodder-quality traits to provide a more comprehensive assessment of livestock-feeding value (Habyarimana et al., 2018). Before analysis at ICRISAT, all field data underwent routine quality control and unit standardization.

### 2.4 Statistical analysis

#### 2.4.1 Stability and adaptability

Stability and adaptability were assessed primarily for GY and FW, as these represent the principal grain and green-fodder outputs of dual-purpose sorghum and pearl millet (Olivoto and Nardino, 2021). Additional traits were used to support biological interpretation.

Genotype × environment interaction was evaluated within a two-way framework in which environments were defined as location × season/sowing-window combinations. Several complementary parametric and multivariate methods were used to characterize stability and adaptation: (i) Finlay-Wilkinson regression assessed genotype responsiveness to environmental productivity (Malosetti et al., 2013); (ii) GGE biplots were used to visualize which-won-where patterns, relationships among environments, and mean-stability relationships (Yan and Tinker, 2006); (iii) the weighted average of absolute scores from BLUPs (WAASB) was calculated from the singular-value decomposition of predicted G×E effects, with lower values indicating smaller interaction effects and greater stability within the sampled environments (Olivoto et al., 2019; Olivoto and Lúcio, 2020); and (iv) Wricke’s ecovalence and Shukla’s stability variance were computed as supporting stability indices (Shukla, 1972). Given the limited test set, WAASB and GGE patterns were interpreted within the sampled environments, with GGE sectors treated as preliminary response groups requiring further validation.

These complementary tools were interpreted jointly. WAASB summarized the magnitude of predicted G×E interaction associated with each genotype; GGE biplots provided a visual interpretation of genotype performance and environment discrimination; and Pareto-frontier analysis identified entries for which no other evaluated genotype simultaneously exceeded both GY and FW.

##### DualIndex

To enable simultaneous selection for grain and green-fodder performance, a standardized dual-trait selection index was constructed by combining z-score-standardized GY and FW:


DualIndex = 0.5 × ZGY + 0.5 × ZFW

Equal weighting was used because the study addressed a locally defined dual-purpose target product profile in which grain and green fodder were both prioritized. In the absence of validated Saudi Arabia-specific economic weights, market-based trait values, or farmer-preference scores, assigning unequal weights would have introduced unsupported assumptions about the relative importance of grain and fodder. The 50:50 DualIndex, therefore, provides a transparent and reproducible baseline for balanced selection. Standardizing both traits before index construction placed GY and FW on a common scale and prevented the larger numerical range or variance of biomass from disproportionately influencing the index. Equal weighting provides a transparent baseline that can be refined using context-specific farmer preferences, feed demand, market values, labor requirements, and production risks.

##### Pareto analysis

Because any single weighted index can favor one production objective depending on the selected weights, Pareto-frontier analysis was employed as a complementary weight-free approach to evaluate GY-FW trade-offs (Pareto, 1896; Deb, 2001; Moeinizade et al., 2020). A genotype was classified as Pareto-efficient, or non-dominated, when no other evaluated genotype simultaneously exceeded it for both traits.

#### 2.4.2 Variance components and dependability metrics for grain yield and fresh biomass

For each crop × target trait combination, GY and FW plot-level data were analyzed across all usable environments using a linear mixed model that accommodated the alpha-lattice structure within each environment. Summary outputs were converted from kg ha^-1^ to t ha^-1^ where appropriate, while statistical analyses were performed using the original plot-level data. Environments were treated as fixed effects, while genotype and G×E were modeled as random effects to partition genetic and interaction variance and estimate selection reliability. Replication and incomplete-block effects were included within environment. The model was specified as:


$$Yijkl=\mu +Ej+Gi+\left(G\times E\right)ij+Rk\left(j\right)+Bl\left(kj\right)+\epsilon ijkl$$

where μ is the overall mean; Ej is the fixed effect of environment j; Gi is the random effect of genotype i; (G×E)ij is the random genotype × environment interaction; Rk(j) is replication nested within environment; Bl(kj) is incomplete block nested within replication and environment; and ϵijkl is the residual error.

Variance components were estimated using restricted maximum likelihood (REML), including genotypic variance (σ^2^G), genotype × environment variance (σ^2^GE), and residual variance (σ^2^e). For each balanced crop × trait dataset, with e usable environments and r replications per environment, entry-mean broad-sense heritability was calculated as:


$$H^{2}entry=\sigma^{2}G/[\sigma^{2}G+(\sigma^{2}GE/e)+(\sigma^{2}e/er)]$$

where σ^2^G, σ^2^GE, and σ^2^e denote the genotypic, genotype × environment, and residual variance components, respectively. In the present analyses, r = 3; e = 2 for sorghum grain yield; and e = 4 for sorghum fresh biomass and for both target traits in pearl millet. H^2^entry was interpreted as the reliability of entry means under the sampled environments, replication structure, data completeness, and fitted model (Barreto et al., 2024). The coefficient of variation (CV%) was reported as an additional measure of experimental precision (Malosetti et al., 2013; Covarrubias-Pazaran, 2019).

Adjusted genotype means and predicted genetic effects were subsequently used for stability assessment, index construction, and graphical interpretation. This analytical workflow separated three complementary questions: (i) mean performance, (ii) stability within the sampled environments, and (iii) grain-green-fodder trade-offs.

To test systematic sources of variation, a supplementary ANOVA was conducted using Type III sums of squares with sum-to-zero contrasts, allowing for imbalance caused by missing plots or incomplete genotype representation across environments. The REML mixed model remained the primary framework for estimating genetic and G×E variance components, predicting genetic effects, and calculating H^2^entry. Type III ANOVA was retained only as a supplementary fixed-effect classification of environment, genotype, and G×E, and its P-values were not used to estimate or test the REML random-effect variance components. For each crop × trait combination, the study reports the numbers of environments, genotypes, usable observations, trait mean and CV%, H^2^entry, supplementary Type III P-values, and REML variance components. The pest and disease catalogues produced in this work are complementary extension outputs and are retained as **Supplementary Materials S3**, **S4**.

## 3 Results

### 3.1 Genotype, environment, and G×E effects and selection reliability

BLUP-based model-adjusted entry means, adjusted for replication, block, and field heterogeneity and averaged over usable environments, are reported in **Supplementary Materials S1**, **S2**. Supplementary Type III ANOVA indicated significant environment, genotype, and G×E effects (P < 0.001) for grain yield and fresh biomass in both crops (**Table 4**), while the REML mixed model provided the variance components and entry-mean reliability estimates summarized in **Tables 4**, **5**. The significant G×E terms indicate heterogeneous genotype responses among the sampled environments; evidence of actual rank crossover was evaluated separately using the stability statistics and GGE patterns. These findings support joint consideration of mean performance, stability, and grain-biomass trade-offs rather than reliance on single-environment or single-trait rankings (Malosetti et al., 2013; Yan and Kang, 2002).

**Table 4:** Dependability summary for grain yield (GY) and fresh biomass (FW).

Crop	Trait	Env (n)	G (n)	Obs (n)	Mean (t ha^-1^)	CV (%)	H^2^	*P* (Env)	*P* (G)	*P* (G×E)
Sorghum	GY	2	27	162	0.815	48.4	0.238	1.9e–32	3.2e–09	2.6e–07
Sorghum	FW	4	27	324	25.59	20.0	0.916	7.3e–64	2.0e–27	2.3e–44
Pearl millet	GY	4	15	180	1.10	32.2	0.886	5.4e–31	1.4e–12	3.8e–21
Pearl millet	FW	4	15	180	9.09	25.6	0.829	3.5e–30	1.2e–15	1.2e–24

H^2^ = entry-mean broad-sense heritability calculated from REML mixed-model variance components as H^2^entry = σ^2^G/[σ^2^G + (σ^2^GE/e) + (σ^2^e/er)], where e is the number of usable environments and r is the number of replications per environment. P-values are supplementary omnibus tests from Type III ANOVA with sum-to-zero coding and should not be interpreted as tests of the REML random-effect variance components. G (n) and Obs (n) denote the numbers of entries and usable plot-level observations for each crop × trait combination. Obs (n) equals genotypes × usable environments × replications for complete crop × trait datasets. Means were converted to t ha^-1^ for readability.

**Table 5:** Variance components (REML mixed model) supporting H^2^.

Crop	Trait	σ^2^_G_	σ^2^_GE_	σ^2^_e_
Sorghum	Grain yield	54,833.8	324,532.0	81,022.0
Sorghum	Fresh biomass	33,688,985.6	6,023,810.5	18,774,943.4
Pearl millet	Grain yield	93,738.9	14,449.8	102,016.2
Pearl millet	Fresh biomass	4,542,033.6	2,576,152.7	3,552,496.6

σ^2^ estimates were obtained from the REML mixed model with environment fixed and genotype and G×E random; replication and incomplete-block effects were accounted for within environment where applicable.

Trial precision and selection reliability varied markedly among crop × trait combinations (**Tables 4**, **5**). Sorghum fresh biomass showed a strong genetic signal (CV = 20.0%) and very high entry-mean heritability (H^2^ = 0.916), supported by a large genotypic variance (σ^2^G) relative to G×E and residual variances. Pearl millet grain yield also exhibited high dependability (H^2^ = 0.886; CV = 32.2%), with σ^2^G exceeding σ^2^GE, while pearl millet fresh biomass showed high entry-mean reliability (H^2^ = 0.829; CV = 25.6%).

In contrast, sorghum grain yield had low entry-mean heritability (H^2^ = 0.238; CV = 48.4%), reflecting evaluation in only two grain-evaluable environments and a strong G×E component (σ^2^GE ≫ σ^2^G; **Tables 4**, **5**). Grain yield in **Table 4** was estimated from E3 and E4, whereas the 2023 Jazan trials, E1 and E2, contributed grain-related information through grain weight per five plants rather than plot-level grain-yield estimates. Entry-mean heritability is interpreted as reliability conditional on the present environments, replication structure, data completeness, and magnitude of G×E. The low reliability of sorghum grain yield reflects limited grain-evaluable information and environmental sensitivity rather than the absence of exploitable genetic variation.

Sorghum grain-yield results provide promising early evidence for the DualIndex and Pareto analysis. However, because standardization does not account for trait reliability, grain-inclusive rankings should guide further evaluation rather than final recommendation. Additional grain-evaluable environments and sensitivity analyses will strengthen candidate prioritization. Overall, sorghum fresh biomass and pearl millet grain yield and fresh biomass provided stronger evidence for candidate prioritization within the target production environments than sorghum grain yield.

### 3.2 Dual-purpose ranking using a standardized selection index and Pareto efficiency

To identify genotypes combining grain and green-fodder performance, adjusted means for grain yield and fresh biomass were standardized and combined in the equal-weight DualIndex (DualIndex = 0.5 × Zgrain yield + 0.5 × Zfresh biomass). Pareto-frontier analysis was used as a complementary weight-free approach to identify non-dominated grain-biomass trade-offs.

**Figure 2:**
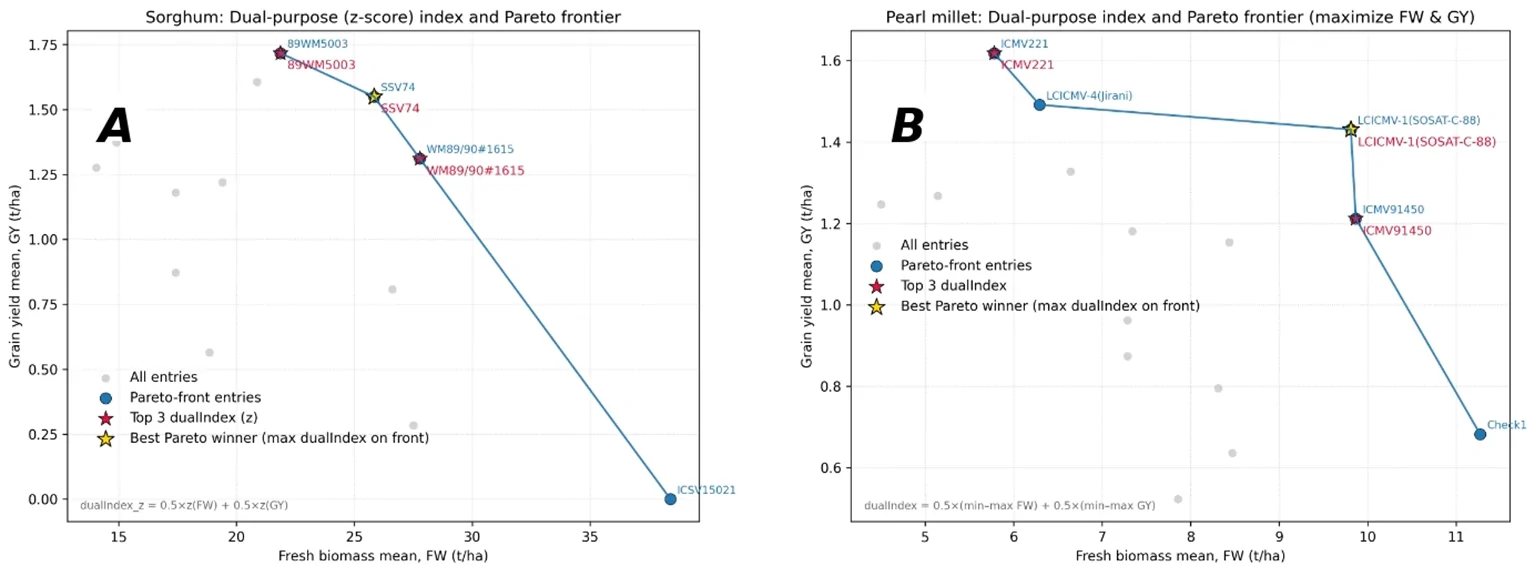
Multi-trait selection for dual-purpose performance in sorghum and pearl millet based on fresh biomass (FW) and grain yield (GY). Genotypes were evaluated using a dual-purpose selection index and Pareto-frontier analysis to identify entries that jointly optimize FW and GY. Scatterplots display mean FW and GY, with the Pareto frontier defining non-dominated genotypes exhibiting favorable trade-offs between the two traits. To maintain readability, labels are shown for Pareto-front entries, the top three DualIndex (*z*) genotypes, and the best Pareto winners; other entries are displayed as background points (refer to **Supplementary Materials S1**, **S2** for detailed performance information on all entries). **(A)** Sorghum and **(B)** pearl millet.

In sorghum, the highest DualIndex values were recorded for SSV 74, WM 89/90#1615, and 89WM 5003, indicating the strongest balanced grain-fresh-biomass profiles within the tested environments. SSV 74 also occurred on the Pareto frontier, indicating that no other evaluated sorghum genotype exceeded it simultaneously for both grain yield and fresh biomass. Because sorghum grain yield was based on only two grain-evaluable environments and had low reliability, these rankings represent preliminary candidate prioritization for expanded validation. In pearl millet, LCICMV-1 (SOSAT-C-88) had the highest DualIndex, followed by ICMV 91450, and occurred among the Pareto-efficient entries relative to the evaluated set (**Figure 2B**). These genotypes are promising candidates within the target production environments for participatory on-farm validation.

The DualIndex provided a transparent baseline for balanced grain-green-fodder selection, while Pareto analysis retained efficient alternative trade-offs. Future participatory and economic assessments can refine the 50:50 weighting by incorporating context-specific farmer, livestock-user, market, and seed-system priorities.

### 3.3 Stability and adaptation patterns for grain and biomass

**Figure 3:**
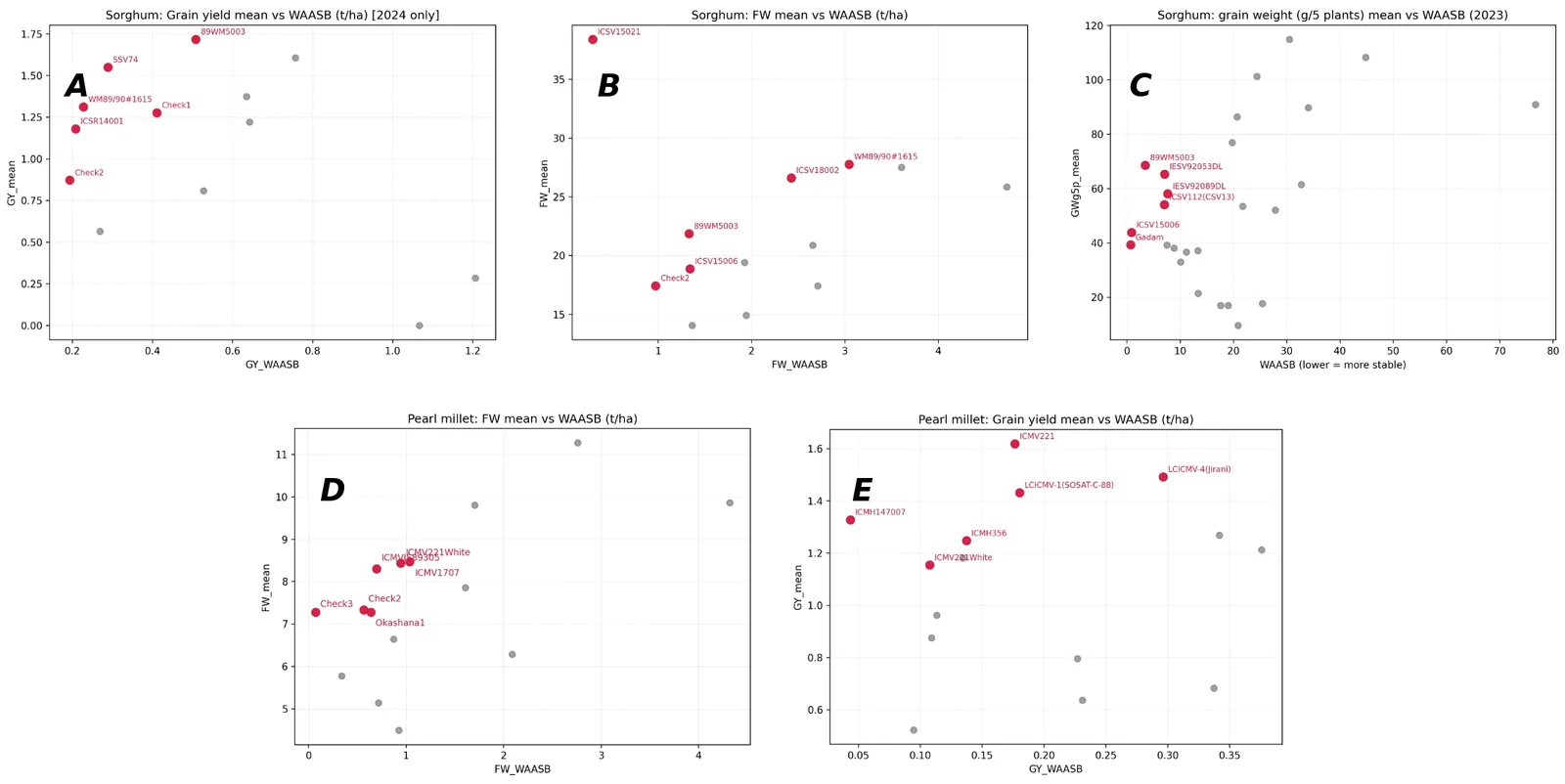
Association between mean performance and yield stability (WAASB) for key agronomic traits in sorghum and pearl millet. Panels show: **(A)** sorghum grain yield mean vs. WAASB (2024), **(B)** sorghum fresh biomass (FW) mean vs. WAASB, **(C)** sorghum grain weight (g per five plants) mean vs. WAASB (2023), **(D)** pearl millet fresh biomass mean vs. WAASB, and **(E)** pearl millet grain yield mean vs. WAASB. Lower WAASB values indicate greater stability across environments. Highlighted genotypes represent selected or check entries, while grey points denote the full set of evaluated genotypes.

**Figure 4:**
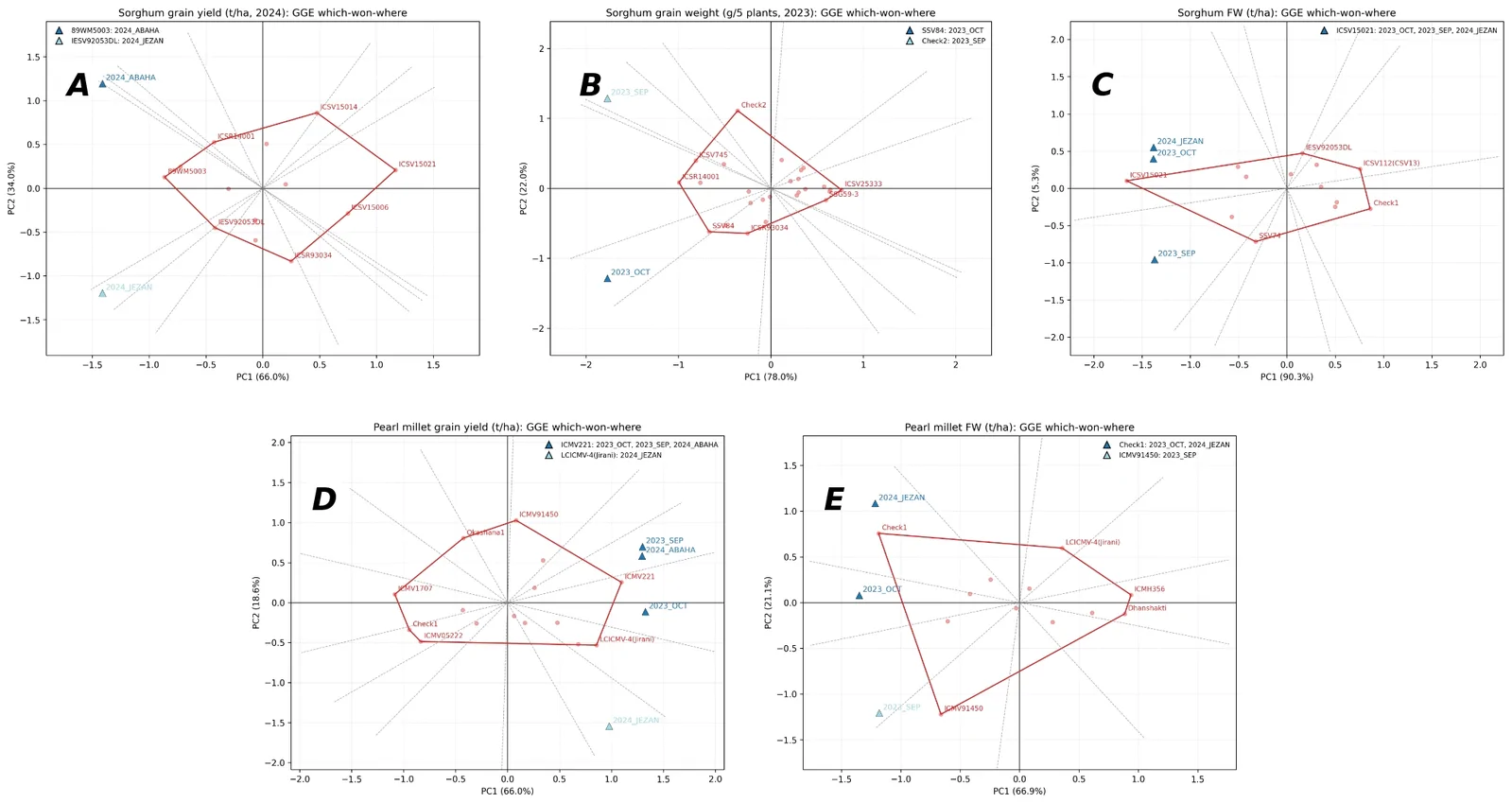
GGE biplot “which-won-where” analysis illustrating genotype performance and mega-environment patterns for key agronomic traits in sorghum and pearl millet across multi-environment trials. Panels depict: **(A)** sorghum grain yield (t ha^-1^, 2024), **(B)** sorghum grain weight (g per five plants, 2023), **(C)** sorghum fresh biomass (FW, t ha^-1^), **(D)** pearl millet grain yield (t ha^-1^), and **(E)** pearl millet fresh biomass (FW, t ha^-1^). In each biplot, polygon-vertex genotypes identify those with the highest performance in specific environments, while sector divisions indicate crossover interactions and potential mega-environments. The first two principal components (PC1 and PC2) capture the majority of genotype × environment interaction for each trait.

Mean-stability relationships based on WAASB and GGE biplots (**Figures 3**, **4**) identified contrasting performance and interaction patterns within the sampled environments. For sorghum fresh biomass, ICSV 15021 combined the highest mean yield (approximately 38.4 t ha^-1^) with the lowest WAASB (approximately 0.30) and favorable GGE performance, supporting its advancement to expanded adaptation testing. For grain yield, 89WM 5003 ranked first (approximately 1.72 t ha^-1^), followed by IESV 92053DL (approximately 1.61 t ha^-1^) and SSV 74 (approximately 1.55 t ha^-1^). Check 2 and ICSR 14001 had the lowest WAASB values, demonstrating that high yield and stability were not always associated. The crossover responses observed between the two grain-evaluable environments highlight the value of further testing across years, sowing windows, and target locations.

In pearl millet, fresh-biomass performance differed among environments. Check 1 recorded the highest mean fresh biomass (~11.3 t ha^-1^), while ICMV 91450 (~9.9 t ha^-1^) and LCICMV-1 (~9.8 t ha^-1^) were among the leading grain-green-fodder candidates; ICMV 221 had the lowest biomass WAASB. For grain yield, ICMV 221 had the highest mean (~1.62 t ha^-1^), followed by LCICMV-4 (Jirani; ~1.49 t ha^-1^) and LCICMV-1 (~1.43 t ha^-1^); ICMH 147007 had a very low WAASB (~0.044). The pearl millet GGE biplots suggested at least two preliminary response groupings, including a Jazan-associated sector in which LCICMV-4 was favored. Within the current evidence base, LCICMV-1 and ICMV 91450 are promising candidates for additional dual-purpose validation, while ICMV 221 and LCICMV-4 merit further grain-focused or environment-targeted evaluation.

## 4 Discussion

This study demonstrates the value of integrating mixed-model reliability estimates, an equal-weight DualIndex, Pareto-frontier analysis, and stability metrics to support dual-purpose sorghum and pearl millet improvement in southwestern Saudi Arabia. Bringing these complementary tools together provides a practical framework for evaluating grain and green-fodder performance simultaneously, identifying promising candidate portfolios, and translating multi-environment data into clear advancement priorities. This integrated application is particularly relevant in arid mixed crop-livestock systems, where sorghum and pearl millet can strengthen food, nutrition, feed, and fodder security under conditions in which more water-demanding cereals are less dependable (Chaturvedi et al., 2022, 2023). By combining productivity, reliability, stability, and trade-off efficiency, the framework advances cultivar evaluation beyond simple mean-performance ranking and establishes a strong analytical foundation for expanded validation and stakeholder-informed selection (Malosetti et al., 2013; Yan and Kang, 2002; Olivoto and Lúcio, 2020).

To our knowledge, this study provides new multi-environment evidence of the potential of introduced sorghum and pearl millet germplasm to improve grain and green-fodder production in the rainfed-supplemented production systems of southwestern Saudi Arabia. The regional importance of these findings is underscored by the central role of Jazan and Asir in national rainfed cereal production and by the continuing need to expand farmers’ access to productive, resilient, and end-use-appropriate cultivars (Food and Agriculture Organization of the United Nations, 2024). The introduced germplasm broadens the range of genetic and production options available for complementing locally adapted landraces. It also creates opportunities to develop cultivar portfolios addressing different farmer objectives, including grain productivity, immediate green-fodder supply, balanced dual-purpose performance, and adaptation to specific production environments.

The contrasting elevation, temperature, rainfall, sowing-window, and management conditions represented in the trials generated meaningful environmental differentiation. Soil electrical conductivity remained below 2 dS m^-1^ at both sites, indicating that soil salinity was unlikely to be the primary limitation to crop performance. Instead, differences in rainfall, temperature, altitude, sowing window, and managed irrigation probably contributed collectively to the observed environmental responses (Abdella et al., 2024; Odnoletkova and Patzek, 2023). Although the four-environment structure did not allow these factors to be separated statistically, the environmental contrasts were sufficient to reveal substantial genetic variation and important differences in genotype response. Thus, the weather and site information provide valuable biological context for interpreting performance patterns and designing a more extensive future testing network.

### High genetic variance and reliability support crop- and trait-specific advancement

The analyses revealed substantial environment, genotype, and G×E effects across the target traits, demonstrating both the availability of useful genetic variation and the importance of multi-environment evaluation. The significant G×E effects showed that genotype responses differed among the sampled conditions, while the WAASB and GGE analyses provided complementary information on the nature of those response patterns. Together, these results confirm that candidate selection in southwestern Saudi Arabia will benefit from combining mean performance with reliability and stability rather than relying on single-environment observations.

Selection reliability was particularly strong for sorghum fresh biomass, with an entry-mean heritability of 0.916. Pearl millet grain yield also showed very high reliability at 0.886, while pearl millet fresh biomass had high reliability at 0.829. These values indicate that the trials captured strong and repeatable genetic differences for traits directly relevant to grain and green-fodder production. The results, therefore, provide a sound basis for advancing biomass-focused sorghum and grain and dual-purpose pearl millet candidates to broader testing (Bomma et al., 2024; Kanatti et al., 2022; Ma et al., 2024).

The strong genetic signal for sorghum fresh biomass is especially important for mixed crop-livestock systems, where timely fodder availability can be as important as grain production. Genotypic variance was large relative to G×E and residual variance, indicating that differences among sorghum entries were expressed comparatively consistently within the tested environments. Similar results have been reported in forage sorghum studies, where substantial genetic variation and BLUP-based stability analyses supported effective selection for forage-yield traits despite significant environmental interaction (Behera et al., 2024a, 2024b). The present findings confirm that fresh-biomass improvement represents an immediate and promising entry point for strengthening dual-purpose sorghum production in southwestern Saudi Arabia.

Sorghum grain yield showed lower entry-mean reliability at 0.238 because plot-level grain yield was available for only two environments and G×E variance was large relative to genotypic variance. Rather than diminishing the contribution of the study, this result provides a clear and actionable diagnostic for strengthening the next testing phase. It shows that additional grain-evaluable environments are required to distinguish repeatable genetic differences from environment-specific responses with greater precision. The current grain-yield results can therefore guide the selection of promising entries for expanded evaluation while ensuring that final grain-focused advancement decisions are based on a broader environmental sample.

Grain formation is particularly sensitive to the timing of heat and moisture stress during flowering and grain filling. By contrast, vegetative biomass integrates crop growth over a longer period and may retain a stronger genetic signal under managed water support (Malosetti et al., 2013; Serba and Yadav, 2016). This biological distinction helps explain why sorghum biomass was more reliable than grain yield in the present trials. It also supports a differentiated advancement strategy in which high-confidence biomass candidates can proceed to broader validation while grain-focused conclusions are strengthened through additional testing.

### Valuable portfolio of candidate genotypes identified

The combination of mean performance, entry-mean reliability, WAASB, GGE patterns, DualIndex values, and Pareto efficiency identified several promising materials with complementary production profiles. This portfolio-based outcome is one of the study’s principal strengths because farming systems differ in their requirements for grain, fodder, maturity, stability, and location-specific adaptation. Rather than seeking a single universally superior cultivar, the results provide a set of candidate options that can be matched to contrasting product profiles and production environments.

ICSV 15021 emerged as a particularly strong forage-oriented sorghum candidate. It combined the highest mean fresh biomass, approximately 38.4 t ha^-1^, with the lowest WAASB, approximately 0.30. This combination of high productivity and comparatively small G×E effects within the tested environments provides a strong rationale for advancing ICSV 15021 to expanded multi-location and participatory on-farm evaluation. Limited seed increase would be valuable for supplying these validation trials and accelerating the collection of farmer, livestock-user, and fodder-quality evidence.

SSV 74, WM 89/90#1615, and 89WM 5003 had the highest sorghum DualIndex values, identifying them as promising candidates for balanced grain-green-fodder performance. The occurrence of SSV 74 on the Pareto frontier further indicates that no other evaluated sorghum genotype exceeded it simultaneously for both grain yield and fresh biomass. These materials provide useful starting points for broadening the dual-purpose sorghum portfolio. Their grain-related performance should now be verified across additional seasons, sowing windows, and farmer-managed fields, while their strong biomass profiles can be examined in more detail through standardized dry-matter and feed-quality assessment.

The pearl millet results were similarly promising. LCICMV-1 (SOSAT-C-88) achieved the highest DualIndex and displayed a favorable grain-green-fodder profile, while ICMV 91450 provided another attractive dual-purpose option. ICMV 221 recorded the highest mean grain yield, approximately 1.62 t ha^-1^, and represents a strong candidate for grain-focused validation. ICMH 147007 showed a very low grain-yield WAASB, suggesting comparatively small G×E effects within the sampled environments. LCICMV-4 (Jirani) performed favorably in the Jazan-associated GGE sector and therefore merits targeted validation under comparable lowland production conditions.

Collectively, these genotypes constitute a strategically useful portfolio. ICSV 15021 addresses high green-fodder productivity; SSV 74, WM 89/90#1615, and 89WM 5003 represent balanced sorghum options; LCICMV-1 and ICMV 91450 provide dual-purpose pearl millet alternatives; ICMV 221 addresses grain productivity; and LCICMV-4 offers potential for targeted environmental deployment. This diversity of candidate profiles can increase the likelihood that future cultivar development responds effectively to the varied priorities of farmers, livestock producers, extension services, and seed systems.

### A practical framework for staged candidate advancement

The study also provides a useful foundation for structuring advancement decisions. A two-stage scheme could first use on-station MET data to retain high-ranking DualIndex and Pareto-efficient entries while considering reliability and stability. A subsequent stage could evaluate a smaller candidate set under farmer-managed conditions, incorporating farmer preferences, production risks, and relevant economic information. This staged approach can increase testing efficiency by directing resources toward entries with the strongest combined evidence.

Illustrative thresholds, such as retaining the top 20 to 30% of entries, applying a 10 to 15% selection intensity in later testing, assessing superiority relative to local checks, or requiring success across a defined proportion of site-seasons, provide useful starting points for pipeline planning. These thresholds can be progressively calibrated as the evidence base expands. Additional MET data, acceptable production risk, product-profile requirements, farmer preferences, market information, and the performance of relevant local checks can all be used to convert provisional guidelines into locally validated advancement criteria.

Reliability thresholds and WAASB rankings can similarly serve as decision aids rather than fixed release rules. Their practical value lies in making candidate advancement transparent and reproducible. For traits with high reliability, such as sorghum fresh biomass and pearl millet grain yield, stronger advancement decisions can be made from the present evidence. For sorghum grain yield, the same framework identifies the need for further testing before high-confidence decisions are reached. In this way, the analytical approach does not merely rank genotypes; it also indicates the strength of evidence supporting each decision and identifies the most important information gaps.

### Fresh biomass provides an efficient and relevant proxy for biomass productivity

A major strength of the study is the explicit integration of grain yield and fresh biomass into dual-purpose selection. Fresh biomass was selected because green fodder was a priority output in the locally defined target product profile and because the trait is directly relevant to farmers and livestock producers who use freshly harvested plant material for feeding. It is also readily measurable at the plot level and supports rapid evaluation of many genotypes across environments.

Direct measurement of dry biomass yield in large field trials requires destructive sampling, handling of large volumes of harvested material, substantial drying capacity, and sufficient time to bring samples to constant weight. These requirements can constrain throughput and limit the number of entries and environments that can be evaluated. Fresh biomass, therefore, offers an important operational advantage in breeding programs and METs.

Crucially, fresh biomass yield is generally strongly associated with dry biomass yield at both phenotypic and genetic levels in sorghum (Oliveira et al., 2020; Habyarimana et al., 2018). Fresh and dry biomass were strongly correlated in both crops, with coefficients of *r* = 0.92 in sorghum and *r* = 0.98 in pearl millet. These relationships are consistent with reported correlations of *r* = 0.77 to 0.78 in sorghum (Nazli et al., 2024) and *r* = 0.63 to 0.79 between green and dry forage yields in pearl millet (Govintharaj et al., 2018). This strong relationship means that genotypes producing greater fresh biomass also tend to produce greater dry matter, supporting fresh biomass as a practical proxy for dry biomass productivity. The high entry-mean reliability observed for sorghum and pearl millet fresh biomass yields in the present study further strengthens its value as a selection trait.

In the current trials, dry-biomass observations were recorded, but the large number and volume of samples could not be oven-dried under controlled conditions to constant weight because suitable drying facilities were unavailable at the trial sites. The resulting dry-mass observations were therefore not sufficiently standardized for rigorous genotype comparisons. Under these logistical conditions, fresh biomass was the most consistently measured and operationally relevant fodder-yield trait available for the DualIndex.

The index should consequently be interpreted as measuring grain and green-fodder productivity. This interpretation aligns closely with the intended deployment context, in which immediate green-fodder availability was a priority. The use of fresh biomass, therefore, represents a practical strength of the evaluation rather than simply a methodological compromise. It enabled high-throughput comparison of candidate productivity across a multi-environment trial network while retaining direct relevance to livestock-feeding needs.

Future evaluations can build on this strong foundation by determining fresh-to-dry biomass relationships from representative subsamples dried to constant weight. Measuring dry-matter percentage will help identify genotypes whose moisture content causes deviations from the general fresh-to-dry biomass relationship. Fodder assessment can then be progressively expanded to include digestibility, crude protein, neutral and acid detergent fiber, lignin, palatability, and harvestability. Plant height, stem diameter, and direct lodging scores can provide additional information on standability and recoverable grain and fodder yield. These measurements will complement the practical value of fresh biomass and enable the development of increasingly comprehensive dual-purpose product profiles.

### DualIndex and Pareto analysis provide complementary selection perspectives

The equal-weight DualIndex represented the locally defined objective of balancing grain and green-fodder production. In the absence of validated Saudi Arabia-specific economic weights or farmer-preference scores, equal weighting provided a transparent and reproducible baseline. It enabled both traits to contribute explicitly to candidate ranking after standardization and prevented the larger numerical scale of biomass from dominating the index.

The value assigned to grain and fodder will nevertheless vary among farming households and production environments. Farmers with greater livestock ownership or severe seasonal feed shortages may prioritize fodder, while households focused on food supply or grain markets may place more weight on grain yield and quality. Participatory studies can build directly on the current index by eliciting preferences for grain production, green and dry fodder yield, maturity, palatability, stem juiciness, lodging resistance, threshability, pest and disease response, and production risk. These preferences can be translated into context-specific weights or separate target product profiles.

Pareto-frontier analysis strengthened this framework by preserving genotypes representing alternative efficient grain-fodder trade-offs without requiring additional predetermined weights (Pareto, 1896; Deb, 2001; Moeinizade et al., 2020). A Pareto-efficient genotype is not exceeded simultaneously for both evaluated traits by another entry. This does not establish universal superiority but identifies efficient grain-fodder trade-offs among the evaluated candidates.

Joint interpretation of the DualIndex and Pareto frontier therefore offers a balanced ranking while retaining valuable alternative profiles that could match different farmer and market priorities. As additional information becomes available, sensitivity analyses can compare the original 50:50 index with alternative grain-to-fodder weights, reliability-adjusted indices, and broader multi-trait selection approaches. This will reveal whether candidate rankings remain consistent across decision scenarios and will help identify genotypes that are robust to changes in stakeholder priorities.

### Stability analyses guide future testing and targeting

WAASB and GGE analyses provided useful complementary insights into stability and environmental response. WAASB quantified the relative magnitude of predicted G×E effects, while GGE biplots illustrated genotype-environment patterns and possible crossover responses. The emergence of preliminary pearl millet response groupings, including a Jazan-associated sector favoring LCICMV-4, provides a valuable hypothesis for designing the next stage of targeted evaluation.

These response groupings should be validated across additional years and sites before being used to define formal recommendation domains. Nevertheless, their identification is an important outcome because it directs future testing toward specific genotype-environment combinations. Candidate genotypes showing favorable performance across the sampled environments can be evaluated more widely, while entries with apparent environment-specific advantages can be tested intensively in the corresponding production conditions.

The contrast between high mean performance and low WAASB also provides useful breeding information. Genotypes with the greatest yield are not always those with the smallest interaction effects. This highlights the importance of retaining both productivity and stability information in candidate advancement. Farmers and production systems differ in their tolerance for risk, and a portfolio containing both high-potential and comparatively stable options may provide greater value than a single generalized recommendation.

### Managed trials provide a strong platform for farmer-based validation

The rainfed-supplemented, on-station trials provided a controlled and informative platform for comparing genetic performance across contrasting environments. Supplemental irrigation supported uniform establishment, reduced the risk of complete crop failure, and enabled meaningful differentiation among genotypes. Protective netting at Jazan also reduced bird-related losses during grain filling, thereby improving the ability to assess genetic potential.

These management practices create a strong foundation for identifying promising materials before exposing a smaller candidate set to the greater variability of farmer-managed conditions. The logical next step is therefore not to discount the on-station findings, but to use the evidence efficiently in designing targeted validation under fully rainfed and farmer-managed production systems. Such testing will determine whether genotype rankings remain consistent when entries encounter variation in establishment, soil moisture, labor, pest pressure, bird damage, and crop management.

Farmer-managed trials will also provide information that cannot be obtained fully from research-station experiments. These include farmer preferences, labor requirements, ease of harvesting and processing, grain-use quality, livestock acceptance, marketability, and perceived production risk. Integrating these dimensions with the quantitative framework developed here will strengthen the relevance and adoption potential of the selected materials.

### Pathway from promising germplasm to farmer impact

The study has practical implications extending beyond genotype ranking. The identification of promising materials is most valuable when connected to seed availability, extension support, pest and disease management, participatory evaluation, and national registration pathways. The project’s sorghum and pearl millet pest and disease catalogues are therefore useful complementary outputs that can strengthen farmer and extension capacity, even though those resources were not components of the cultivar-evaluation methodology.

Recent Saudi rainfed cereal initiatives have identified weak seed chains, limited access to improved varieties, management gaps, and farmer capacity as major constraints (Food and Agriculture Organization of the United Nations, 2024). The candidate portfolio identified in this study creates an opportunity to address these limitations through a staged seed and validation strategy. Initial seed increase can ensure sufficient experimental seed for multi-location and participatory trials. Candidates demonstrating consistent performance, acceptable quality, and farmer preference can then progress toward registration, pre-release multiplication, and targeted deployment in accordance with national procedures.

This staged pathway links genetic evidence with delivery mechanisms and reduces the time between candidate identification and farmer benefit. ICSV 15021 can advance through forage-focused validation; SSV 74, WM 89/90#1615, and 89WM 5003 through balanced dual-purpose testing; LCICMV-1 and ICMV 91450 through pearl millet dual-purpose evaluation; ICMV 221 through grain-focused testing; and LCICMV-4 through targeted Jazan validation. Maintaining these complementary profiles through the validation pipeline can increase the probability of developing cultivars suited to diverse farmer needs.

### Future priorities

The present study establishes a strong foundation for a progressive sorghum and pearl millet improvement pathway in southwestern Saudi Arabia. Five priorities can build on this foundation.

First, sorghum grain testing should be expanded across additional seasons, sowing windows, locations, fully rainfed environments, and farmer-managed fields. This will improve grain-yield reliability and determine whether the promising grain-inclusive profiles identified here are repeatable. Second, participatory on-farm trials should evaluate the candidate portfolio while collecting structured preference information from farmers, livestock users, extension personnel, and other value-chain participants. These data will support locally relevant product profiles and trait-weighting scenarios. Third, fodder assessment should combine high-throughput fresh-biomass measurement with representative oven-dried samples, dry-matter percentage, digestibility, crude protein, fiber fractions, lignin, palatability, and lodging resistance. This will connect field productivity with actual livestock-feeding value. Fourth, sensitivity analyses should compare alternative grain:fodder weights, reliability-adjusted indices, and expanded multi-trait formulations. Such analyses will identify candidates that remain competitive across different farmer, market, and production objectives. Fifth, seed increase, registration, extension, and deployment activities should be aligned with staged evidence generation. Experimental seed production can support immediate validation, while broader multiplication and scaling can follow confirmation of performance, quality, farmer acceptance, and compliance with national requirements.

Overall, the study delivers two mutually reinforcing outputs: a valuable portfolio of promising sorghum and pearl millet genotypes and a transparent framework for advancing them. The strong reliability of sorghum fresh biomass and pearl millet grain and fresh-biomass performance demonstrates substantial opportunities for near-term progress. The identification of complementary forage, grain, dual-purpose, and environment-targeted candidates creates a clear pathway toward more productive and resilient cereal systems. By combining expanded METs, participatory validation, standardized fodder-quality assessment, and staged seed-system development (Varshney et al., 2017; 2019), the present findings can contribute directly to improved grain and fodder security, stronger mixed crop-livestock systems, and enhanced climate resilience in southwestern Saudi Arabia and comparable arid environments.

## 5 Conclusions

This study demonstrates the strong potential of introduced sorghum and pearl millet germplasm to expand grain and green-fodder options for rainfed-supplemented production systems in southwestern Saudi Arabia. Across four contrasting site × season/sowing-window environments, the integrated use of mixed-model reliability estimates, WAASB and GGE analyses, an equal-weight grain-fresh-biomass DualIndex, and Pareto-frontier assessment provided a transparent and practical framework for identifying promising dual-purpose, grain-focused, and forage-oriented materials. This combined approach represents an important step toward strengthening evidence-based cultivar evaluation and advancement in Saudi Arabian dryland cereal systems.

The study revealed substantial genetic variation and meaningful differences in genotype responses across environments. Particularly strong selection reliability was obtained for sorghum fresh biomass (H^2^ = 0.916), pearl millet grain yield (H^2^ = 0.886), and pearl millet fresh biomass (H^2^ = 0.829). These results provide a robust basis for prioritizing biomass-focused sorghum and grain and dual-purpose pearl millet candidates within the tested environments. Although sorghum grain yield had lower reliability (H^2^ = 0.238) because it was evaluated in only two grain-evaluable environments, the results successfully identified promising materials and established clear priorities for expanded testing.

Several genotypes showed considerable potential for strengthening future grain and fodder production. ICSV 15021 emerged as a particularly promising sorghum candidate for high and stable fresh-biomass production. SSV 74, WM 89/90#1615, and 89WM 5003 displayed favorable grain-green-fodder profiles and merit advancement to broader multi-environment and participatory evaluation. In pearl millet, LCICMV-1 (SOSAT-C-88) and ICMV 91450 showed strong dual-purpose potential, ICMV 221 was the leading grain-yield candidate, and LCICMV-4 (Jirani) demonstrated potential for targeted validation under Jazan-associated conditions. Together, these genotypes provide a valuable portfolio of candidates addressing different production objectives, including grain productivity, green-fodder supply, and location-specific adaptation. Limited seed increase should support their timely evaluation in expanded trials and farmer-managed environments.

A major practical contribution of the study is the demonstration that cultivar advancement can be strengthened by jointly considering mean performance, selection reliability, stability, and grain-fodder trade-offs. The equal-weight DualIndex offers a transparent and reproducible baseline for balanced selection, while Pareto analysis preserves alternative non-dominated production profiles that might otherwise be overlooked by a single weighted index. This framework can be readily refined as farmer and livestock-user preferences, market information, feed requirements, and production-risk considerations become available.

Fresh biomass was an especially relevant and operationally valuable trait in the present study. It directly represented the green-fodder output prioritized in the local target product profile and provided a practical, high-throughput proxy for biomass productivity under large-scale field conditions. Although dry-biomass observations were recorded, the absence of controlled oven-drying facilities prevented samples from being dried to constant weight and therefore limited the reliability of genotype comparisons based on dry mass. The present DualIndex is consequently best interpreted as an indicator of grain-green-fodder productivity. Future evaluations can build on this foundation by appropriately measuring the dry mass fraction of fresh materials, digestibility, crude protein, fiber fractions, lignin, lodging resistance, and other traits relevant to farmers and livestock production.

The findings establish a clear pathway from on-station evaluation to farmer impact. Expanding sorghum grain testing across additional seasons, sowing windows, locations, fully rainfed environments, and farmer-managed fields will strengthen grain-yield reliability and clarify repeatable adaptation patterns. Participatory evaluations will further refine product profiles and trait weights, while standardized fodder-quality assessment will ensure that biomass productivity translates into livestock-feeding value. Aligning these activities with staged seed increase, variety registration, extension support, and seed-system development will accelerate the conversion of the identified candidates into robust, farmer-preferred cultivar options. Collectively, the study provides both promising genetic materials and a practical decision framework for strengthening grain and fodder security, production resilience, and sustainable mixed crop-livestock systems in southwestern Saudi Arabia and comparable arid environments.

## Data Availability

The original contributions presented in the study are included in the article/**Supplementary Material**. Further inquiries can be directed to the corresponding author.
