# Supplementary material for: Index- and Pareto-based multi-trait selection identifies dual-purpose sorghum and pearl millet cultivars for arid agro-ecosystems of Saudi Arabia
Source: Front Plant Sci. 2026 Aug 10;17:1795221. doi: 10.3389/fpls.2026.1795221 (PMC13500633; doi:10.3389/fpls.2026.1795221)
Supplement: Supplementary file 1 [file DataSheet1.pdf]

Supplementary Information

Index- and Pareto-Based Multi-Trait Selection Identifies Dual-Purpose Sorghum and Pearl Millet Cultivars for Arid Agro-Ecosystems of Saudi Arabia

Authors: Ephrem Habyarimana[1]*, Abdullah Alhendi[2], Kakoli Ghosh[2], Niranjan Ravindra Thakur[1], Ashok Kumar Are[2], Rajan Sharma[1], Jagdish Jaba[1], Shashi Kumar Gupta[1], Henry F. Ojulong[3], Riyazaddin Mohammed[4], Falalou Hamidou[4], Ahmed Abdelmahoud[2], Anitha Raman[1], Rashed Almarri[2]

Affiliations:

[1]International Crops Research Institute for the Semi-Arid Tropics, 502324 Patancheru, Telangana, India.

[2]Food and Agriculture Organization of the United Nations, P.O. Box 558, Riyadh 11421, Saudi Arabia.

[3]International Crops Research Institute for the Semi-Arid Tropics, ICRAF House, Gigiri Nairobi, Kenya.

[4]International Crops Research Institute for the Semi-Arid Tropics, Niamey BP 12404, Niger

*Correspondence: Ephrem Habyarimana – Ephrem.Habyarimana@icrisat.org

S1. Sorghum BLUP-based model-adjusted entry means (i.e., adjusted for replication/block/field heterogeneity) and averaged over environments

Entry	DF50	PH	100SW	GY	Brix	FW	DW	SD	Internodes	INL
89WM5003	49.44	186.98	2.55	1213.71	15.16	45590.12	28065.3	13.52	9.35	9.6
CSV745	38.66	162.28	2.41	1056.62	NA	NA	NA	NA	NA	9.75
Check1	43.06	269.17	2.47	1109.97	10.56	38377.45	19594.19	11.81	9.93	10
Check2	44.94	299.79	2.3	1014.54	10.3	37839.83	18955.39	11.43	11.07	10.77
Gadam	58.68	151.43	2.49	1122.03	9.22	31073.23	17233.39	15.42	8.34	7.08
ICSR14001	55.6	156.61	2.96	1078.23	11.14	45443.89	26676.59	15.6	8.98	9.29
ICSR93034	34.28	192.45	2.21	1095.12	NA	NA	NA	NA	NA	10.45
ICSR93036	55.62	272.02	1.9	1056.04	13.75	48971.58	27093.21	13.01	10.6	11.78
ICSV112(CSV13)	37.8	162.25	2.3	1131	NA	NA	NA	NA	NA	9.13
ICSV12019	77.55	228.24	2.13	1022.42	14.54	45297.35	25371.21	15.01	8.41	9.27
ICSV15006	57.61	262.5	2.47	942.88	13.76	54210.37	27648.69	14.45	10.93	11.96
ICSV15014	39.94	215.2	2.6	877.09	NA	NA	NA	NA	NA	12.36
ICSV17371	35.3	195.43	2.39	1040.14	NA	NA	NA	NA	NA	11.78
ICSV18002	37.82	222.58	2.53	1001.5	NA	NA	NA	NA	NA	**13.11**
ICSV25333	66.23	321.34		1018.32	14.55	45900.84	24315.79	13.94	11.81	11.19
IESV91104DL	65.85	221.18	2.37	1036.78	14	49402.87	28343.04	17.87	11.38	10.64
IESV92053DL	31.98	185.11	2.67	1186.54	NA	NA	NA	NA	NA	9.66
IESV92089DL	66.28	217.05	2.45	1055.48	15.41	49238.38	30148.36	16.27	10	9.74
SSG59-3	70.81	297.09	2.12	1115.1	14.51	51216.39	29315.14	10.52	10.08	10.74
SSV74	33.34	227.78	3.11	1174.92	NA	NA	NA	NA	NA	10.37
WM89/90#1615	59.17	213.85	2.47	1118.21	14.74	53040.25	33342.38	14.36	10	10.53

DF50 = days to 50% flowering; PH = plant height (cm); 100SW = 100-seed weight (g); GY = grain yield (kg ha-1); Brix = juice Brix (%); FW = stalk fresh weight or fresh biomass (kg ha-1); DW = stalk dry weight or dry biomass (kg ha-1); SD = stalk diameter (mm); INL = internode length (cm).

S2. Pearl millet BLUP-based model-adjusted entry means (i.e., adjusted for replication/block/field heterogeneity) and averaged over environments

Entry	DF50	PL	1000SW	GY	FW	DW
**Check1**	55.37	27.6	9.74	727.49	25211.64	10823.46
**Check2**	50.01	20.12	11.97	1160.03	12104.13	4764.12
**Check3**	48.73	27.81	12.46	943.11	NA	NA
**Dhanshakti**	49.73	25.12	11.61	1277.54	10058.62	3524.49
**ICMH147007**	50.96	36.81	9.73	1313.67	NA	NA
**ICMH356**	54.86	21.81	10.73	1237.2	10834.45	4141.58
**ICMV05222**	57.1	32.63	10.09	666.97	15644.26	6009.76
**ICMV1707**	56.47	25.83	8.4	714.36	15173.74	6165.47
**ICMV221**	49.37	23.41	11.83	1553.6	10469.32	4316.47
**ICMV221White**	55.67	25.78	10.91	1132.73	12934.03	5415.47
**ICMV91450**	56.21	28.48	10.34	1156.5	12568.08	5949.05
**ICMVIS89305**	51.59	48.3	9.79	908.01	NA	NA
**LCICMV-1(SOSAT-C-88)**	54.99	29.63	10.39	1379.72	15846.8	6171.76
**LCICMV-4(Jirani)**	51.22	25	10.4	1427.51	9125.03	3168.87
**Okashana1**	57.24	26.02	10.44	982.78	15315.63	5787.6

DF50 = days to 50% flowering; PL = panicle length (cm); 1000SW = 1000-seed weight (g); GY = grain yield (kg ha-1); FW = fresh biomass (kg ha-1); DW = dry biomass (kg ha-1).

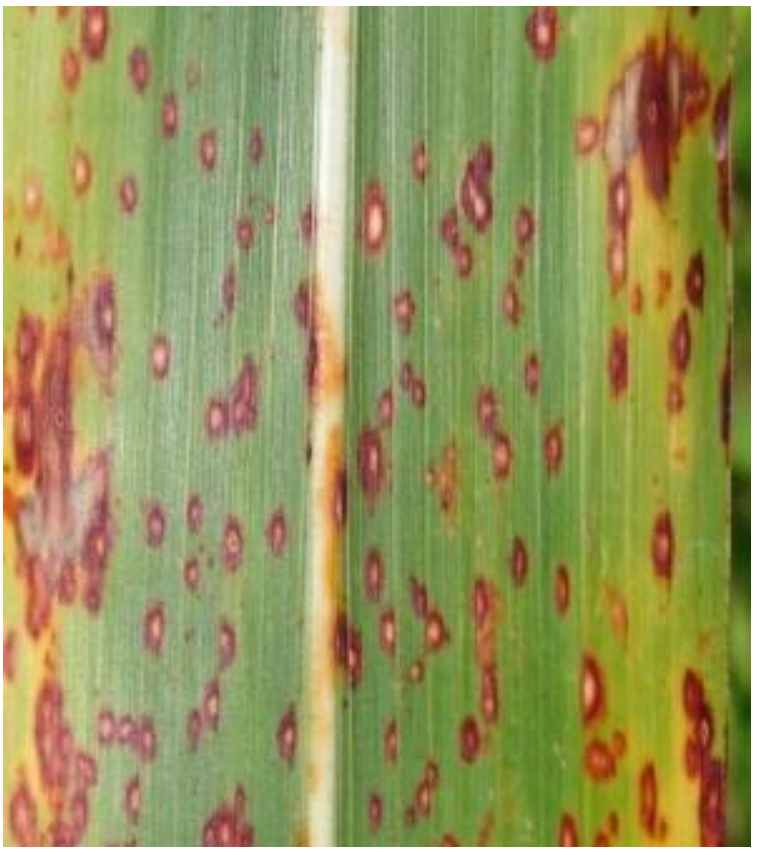
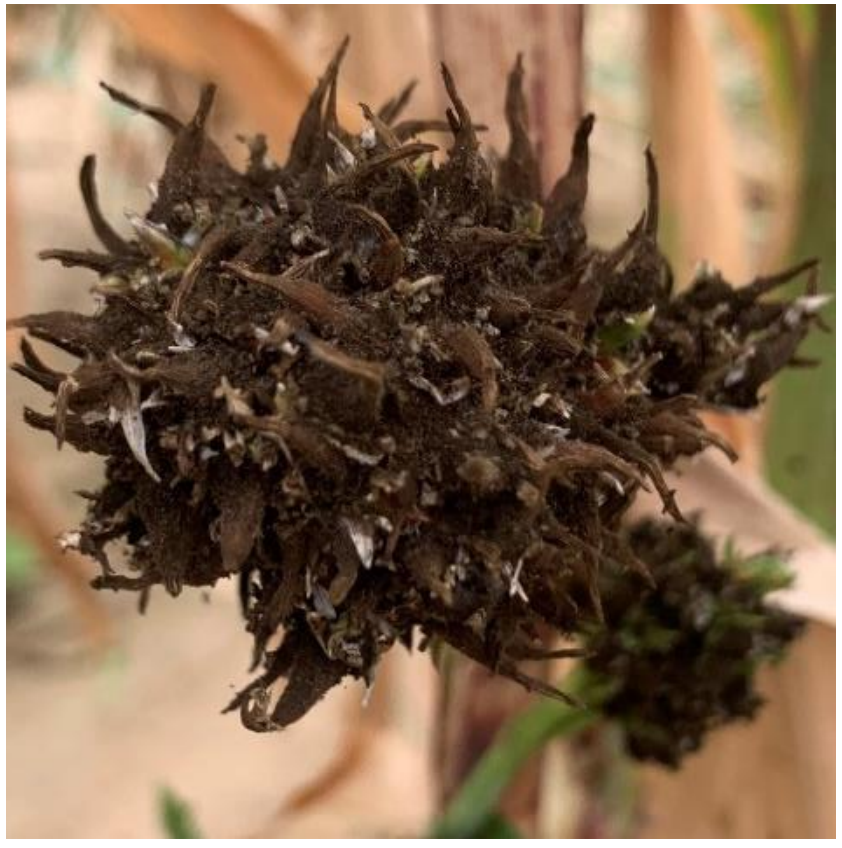
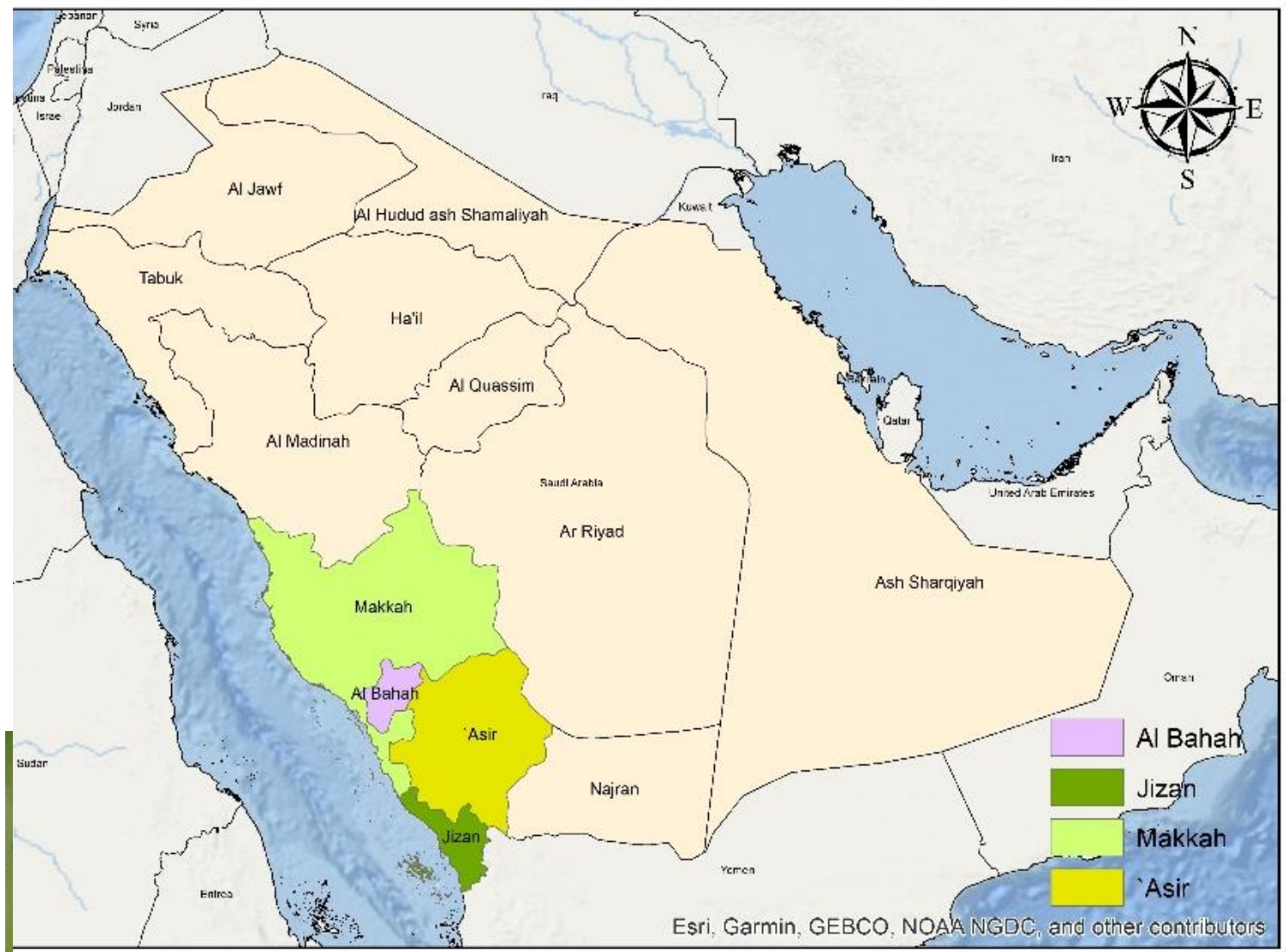

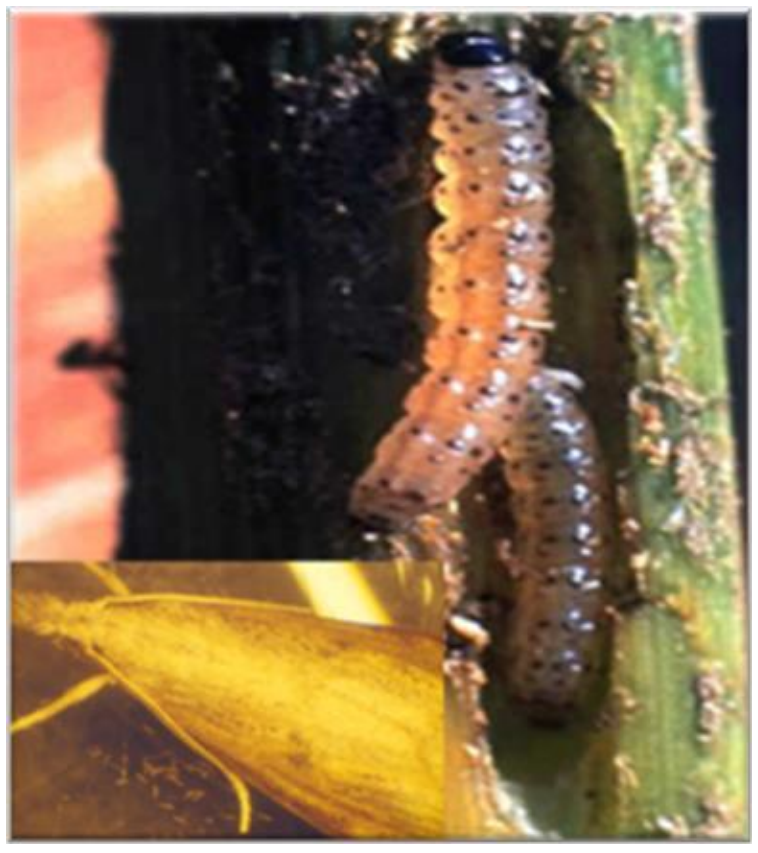
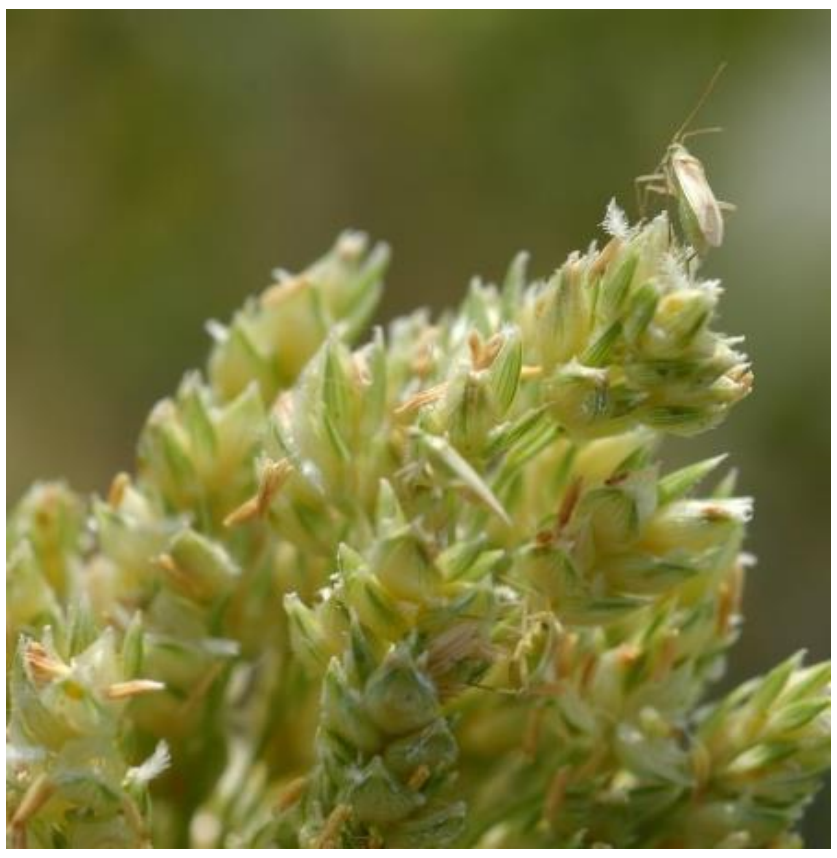
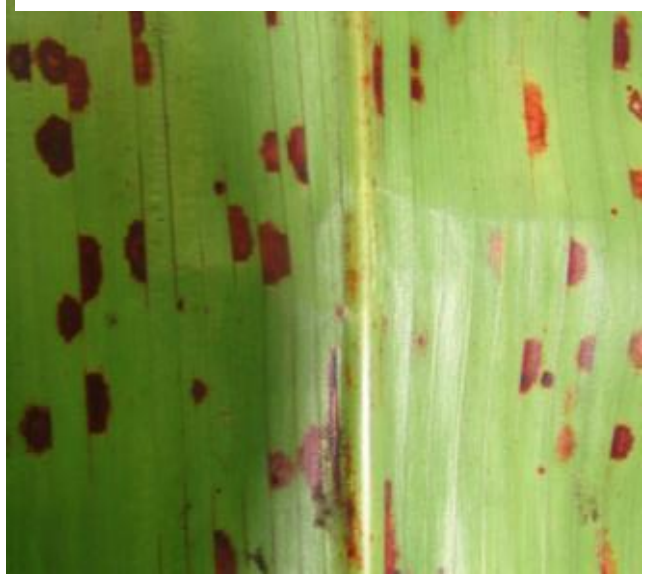
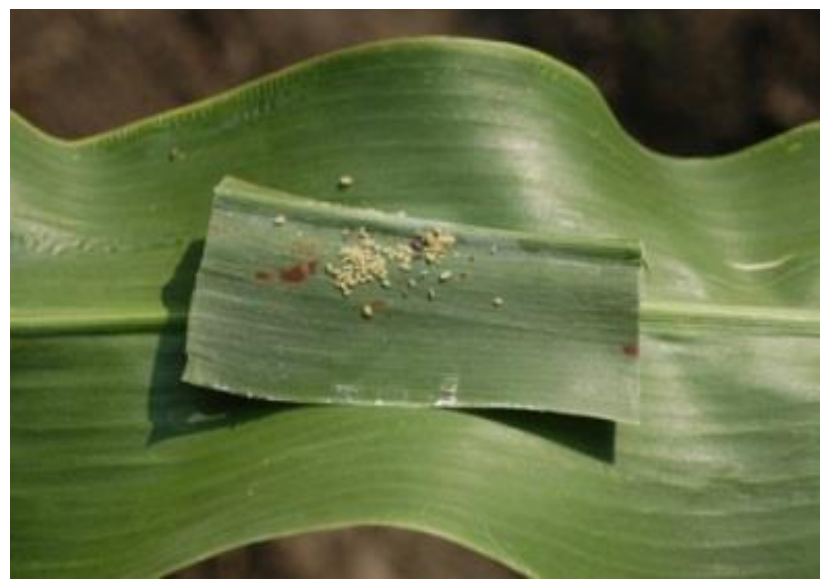

S3. Pests and diseases catalogue - Sorghum

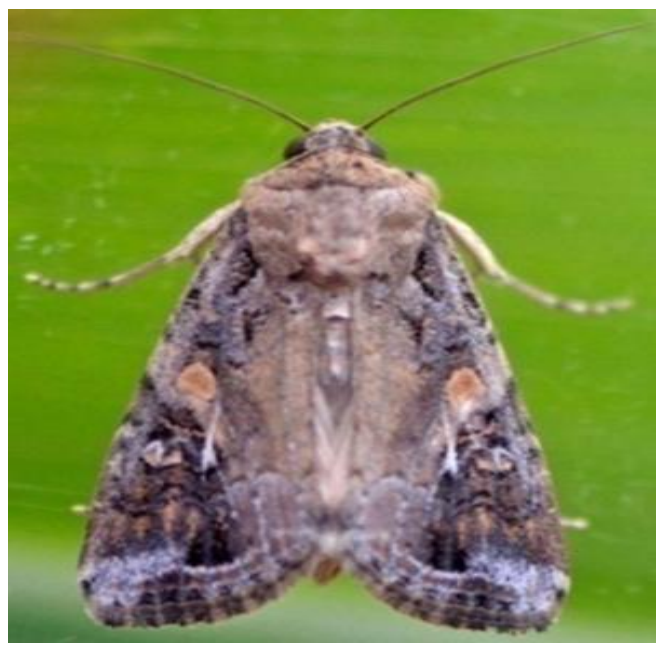

Sorghum is an important millet crop in the semi-arid tropics. Grain yields on farmers' fields in Asia and Africa are generally low mainly due to insect pests and diseases. These diseases can be classified according to the plant parts on which they occur, such as seedling diseases, foliar diseases, root and stalk diseases, leaf sheath diseases, panicle and grain diseases, and storage diseases. Diseases are also classified according to the symptoms they produce on plants and are called seedling blight, root and stalk rots, leaf blight, leaf spot, rust, smut, ergot, wilt, downy mildew, grain mold, leaf stripe, leaf streak, leaf mosaic, etc. Nearly 150 insect species and over 50 diseases have been reported as pests of sorghum, though only a few are economically important globally, several others are regionally and locally important in specific agroecosystems. Among insect pests, shoot fly (*Atherigona soccata*), stem borers (*Chilo partellus* and *Sesamia inferens*), armyworm (*Mythimna separata*), sorghum midge (*Stenodiplosis sorghicola*), head bug (*Calocoris angustatus*), and head caterpillars (*Helicoverpa, Eublemma* and *Pyroderces*) and recent invasive pest fall armyworm (*Spodoptera frugiperda*) are the major pests of sorghum. Sorghum is grown in the South-West of the Kingdom of Saudi Arabia (KSA) mainly in Al Baha, Aseer, Makkah and Jazan. Jazan region grows more than 40,000 ha or around 80% of all sorghum grown in the country. The insect pests and diseases observed in the region are - Chinch bug, aphids, stem borer, fall armyworm, smuts, gray leaf spot, downy mildew, sooty stripe, anthracnose etc. This digital catalogue provides detailed pest/disease description, pictorial representation of damage symptoms of insect pests and diagnostic signs and symptoms of diseases, and integrated pest/disease management options in sorghum.

ICRISAT
INTERNATIONAL CROPS RESEARCH INSTITUTE FOR THE SEMI-ARID TROPICS
FIAT PANIS
Food and Agriculture Organization of the United Nations
وزارة البيئة والمياه والزراعة
Ministry of Environment Water & Agriculture
Kingdom of Saudi Arabia المملكة العربية السعودية

Diseases of sorghum

1. Anthracnose, caused by *Colletotrichum graminicola* (syn. *C. sublineola*)

Pathogen/disease description

The disease is more prevalent and severe in warm and humid environments where it causes substantial economic losses. The disease has been observed in the Jazan region of the KSA. Anthracnose appears on several plant parts. The pathogen causes seedling blight, leaf blight, stalk rot, head blight and grain molding, and thus limits both grain and forage production. Among these, foliar anthracnose is the most pronounced and devastating on the forage and grain sorghum cultivars. Yield losses of 50% or more can occur under severe conditions.

Disease symptoms

- The foliar symptoms appear 30-40 days after emergence. Typical symptoms are small, circular, elliptical or elongated spots usually about 5 mm in diameter. These spots develop gray to straw-colored centers with wide margins that may be tan, red, or blackish purple. In some cultivars that develop low levels of foliar infection, midrib infection occurs more prominently.
- On the centers of the spots, few to numerous small, circular, erumpent black dots develop; these are acervuli of the fungus.
- Elliptical to elongated lesions of straw color, with red purple or black embedded acervuli are seen on the midrib.

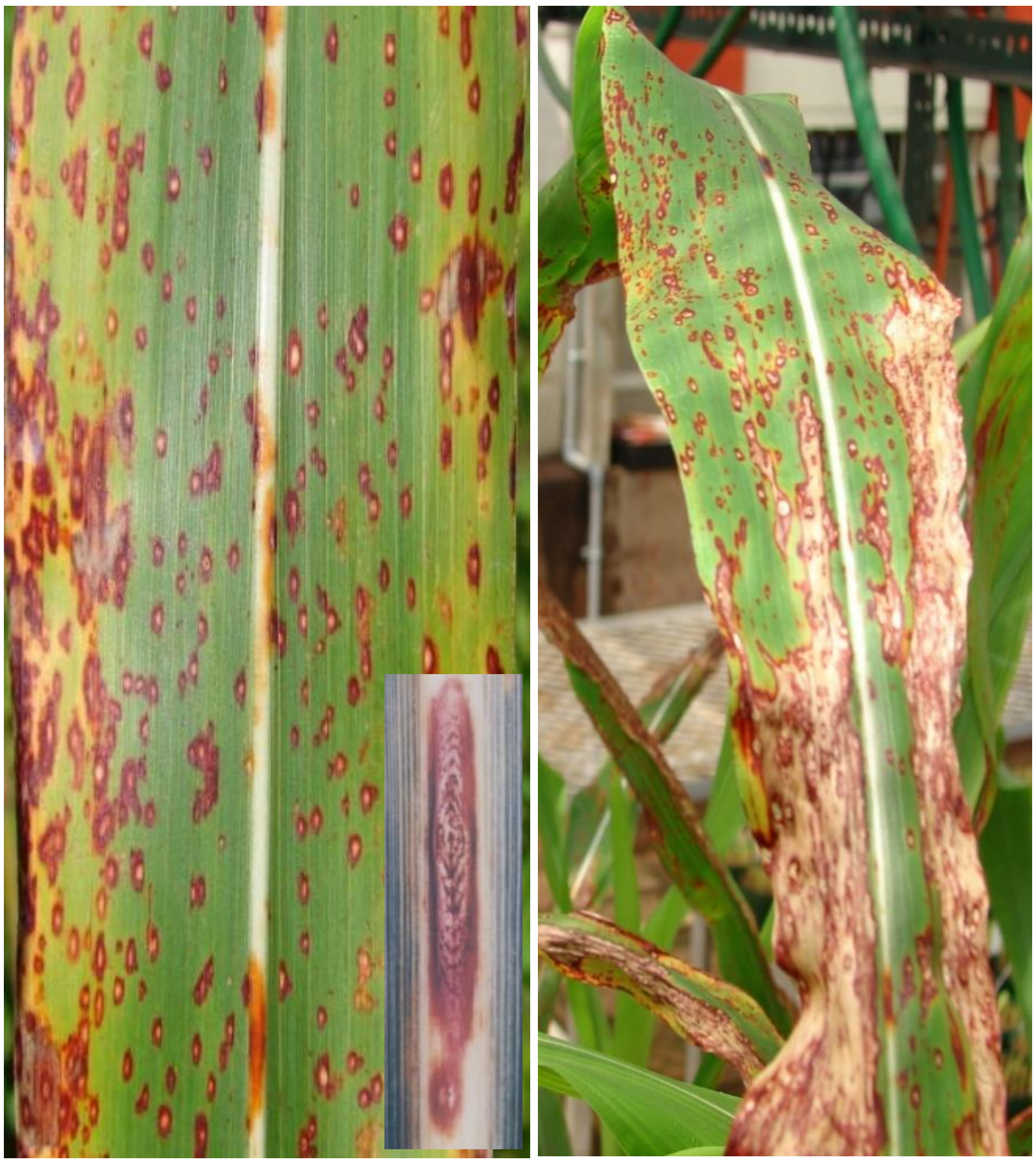

Source: @ICRISAT Rajan Sharma

Management

- Host plant resistance: Several resistant accessions and some tolerant hybrid seed parents, such as ICSA/B 260 to ICSA/B 295 are available at ICRISAT that can be tested in KSA.
- Planting disease free seeds, removal of crop residues and alternate hosts (wild sorghum), weed management, and crop rotation can serve in controlling the disease.
- *Chaetomium globosum* and *Trichoderma harzianum* are effective biocontrol agents that can be tested.
- Fungicides difenoconazole and propiconazole can be tested in KSA for the control anthracnose.

2. Leaf Blight, caused by *Exserohilum turcicum* (syns. *Helminthosporium turcicum; Bipolaris turcica;* and *Drechslera turcica*)

Pathogen/disease description

Leaf blight is an economically important and widespread disease of sorghum in highly humid areas of Asia, Africa and the Americas. The disease has been observed in the Jazan region of the KSA. When infection occurs at preflowering stage in susceptible cultivars, grain yield losses of up to 50% may occur. The pathogen can also infect maize, Johnson grass, teosinte and Sudan grass. However, there are some host-specific races/pathotypes known that do not infect other crop species. The pathogen survives in the field in crop debris as mycelia and conidia in the tropics and as chlamydospores in temperate climate. Minimum temperatures between 14 and 16°C, mean temperatures of 20-22°C and high humidity or heavy dew are most favorable for disease development.

Disease symptoms

- Symptoms are visible from the seedling stage to the crop maturity stage. Small, reddish or tan spots develop on seedlings, the spots later enlarge and coalesce resulting in wilting of young leaves.
- On mature plants, long, elliptical, reddish purple or yellowish lesions develop, first on lower leaves and later progress to the upper leaves and stem as well.
- Lesion size varies depending on resistance levels of the host genotypes, virulence levels of pathogen isolates and prevailing weather conditions.
- In humid weather, numerous grayish black spores are produced in the lesions in concentric zones.

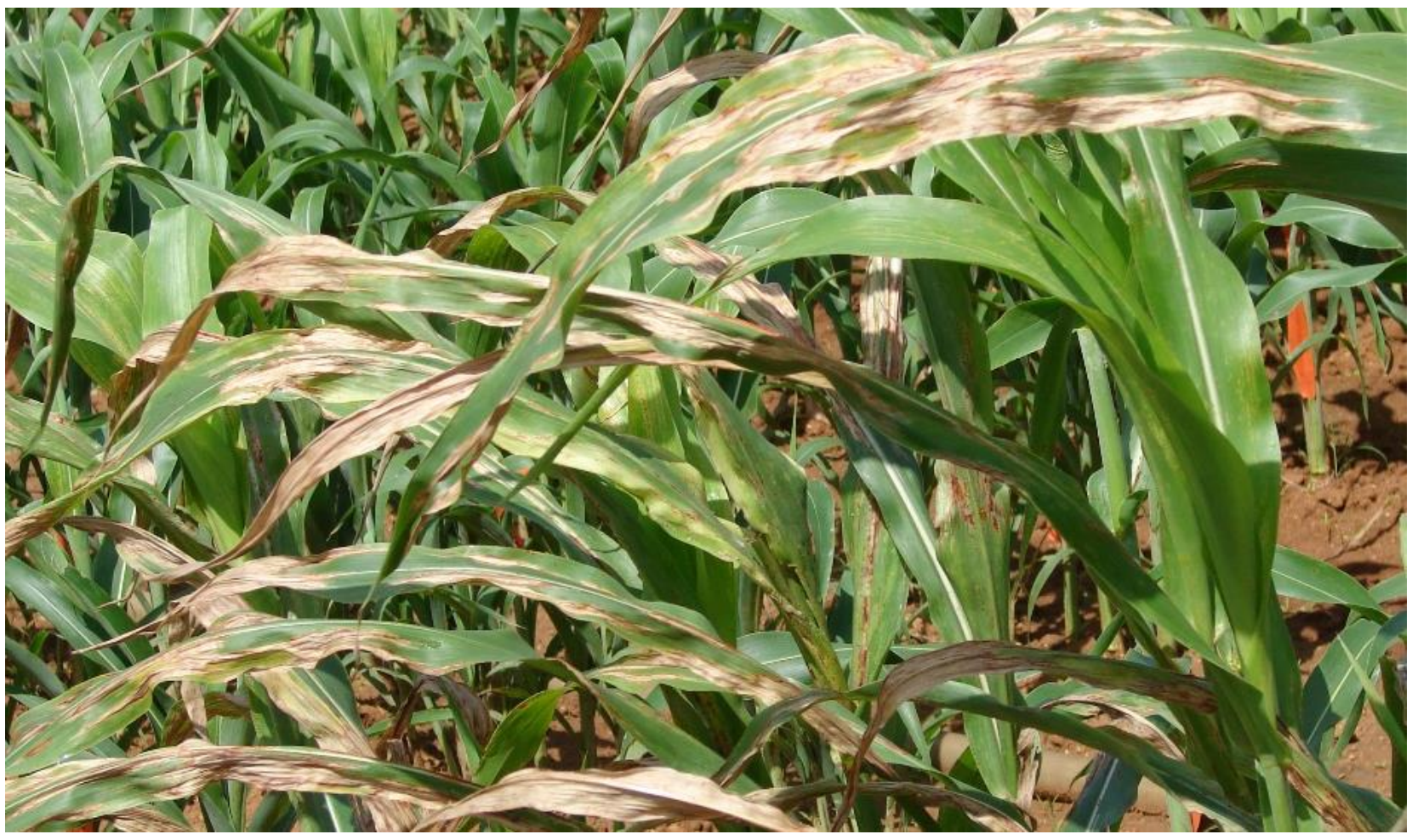

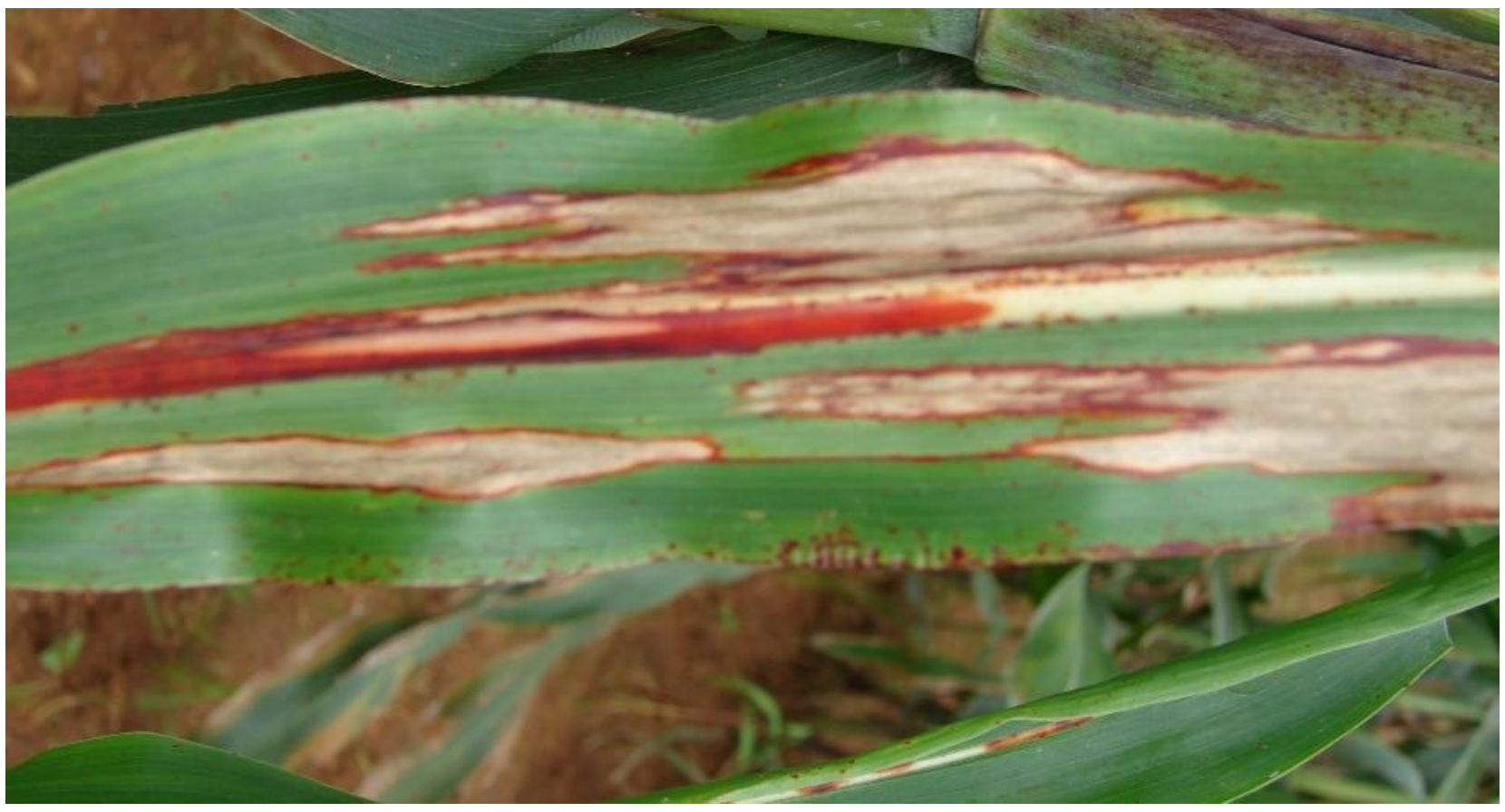

Source: @ICRISAT Rajan Sharma

Management

- Host plant resistance: Several resistant accessions and some leaf blight tolerant hybrid seed parents, such as ICSA/B 296 to ICSA/B 328 were developed during 1989 to 1998 and are available at ICRISAT which need to be tested in KSA.
- Fungicides registered in KSA for sorghum need to be tested for their efficacy against leaf blight.

3. Loose Kernel Smut, caused by *Sporisorium cruentum* (syn: *Sphacelotheca cruenta*)

Pathogen/disease description

Loose kernel smut is common in most sorghum growing regions except Australia and some parts of Asia. The disease has been observed in the Jazan region of the KSA. Loose kernel smut attacks all groups of sorghums, including Johnson grass, although certain varieties in some groups are immune or highly resistant. However, the percent infection under field conditions is usually relatively low, often less than 10%.

Disease symptoms

- Normally, all kernels in an infected panicle are smutted. Individual infected kernels are replaced by small smut sori that are 2.5 cm or longer, pointed and surrounded by a thin gray membrane.
- This membrane usually ruptures soon after the panicle emerges from the boot to release powdery, dark brown to black teliospores.
- These teliospores can be blown away by wind leaving a long, black, pointed, conical, often curved structure (columella) in the center of the gall.
- Infected plants are stunted, panicles on them appear earlier than the healthy plants.

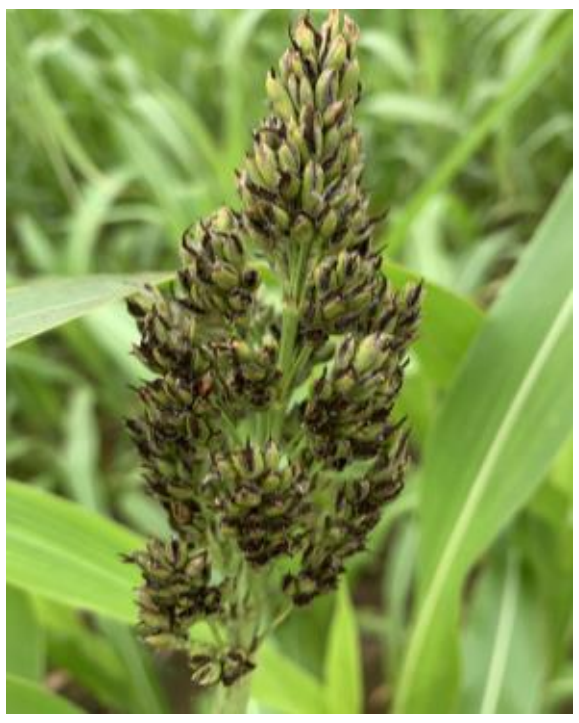

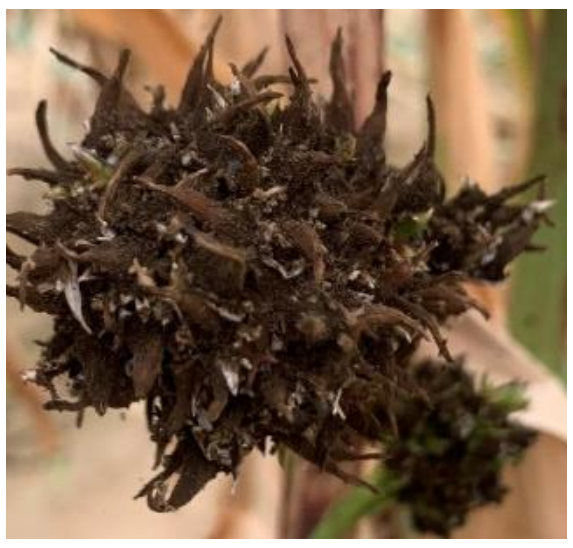

Source: KSA Survey Report

Management

- Smutted panicles are to be burnt or buried in the soil as and when they are observed to prevent the spread of the disease.
- Seed treatment with carboxin @ 2 g/kg seed is effective for the management of loose smut.

4. Covered Kernel Smut, caused by *Sporisorium sorghi* (syn. *Sphacelotheca sorghi*)

Pathogen/disease description

Covered kernel smut is a widespread disease in all sorghum growing regions and was also observed in Jazan region of KSA. Optimum temperature of 25 °C and half moistened soil during planting are most important for the development of covered smut. It infects all groups of sorghum, including Johnson grass. The pathogen is seedborne and infection is systemic, which begins at the seedling stage and progresses to the inflorescence.

Disease symptoms

- Normally, in an infected panicle individual ovules are replaced by conical to oval smut sori that are covered by persistent peridia that are larger than normal grain.
- Initially each sorus is covered with a light pink/silver-white membrane, which later ruptures to reveal brownish-black spores.
- Smut sori are generally smooth, oval, conical or cylindrical, and vary in size.

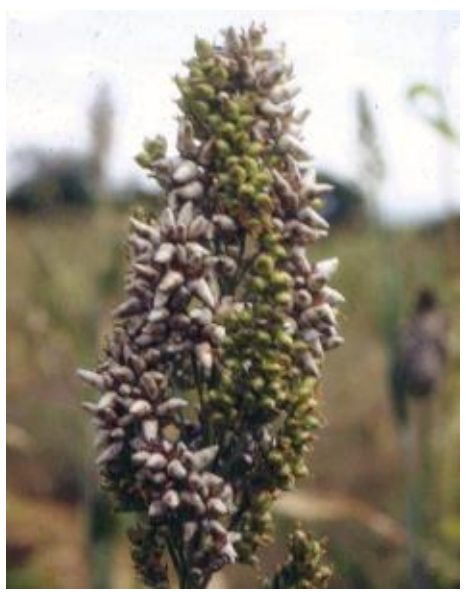

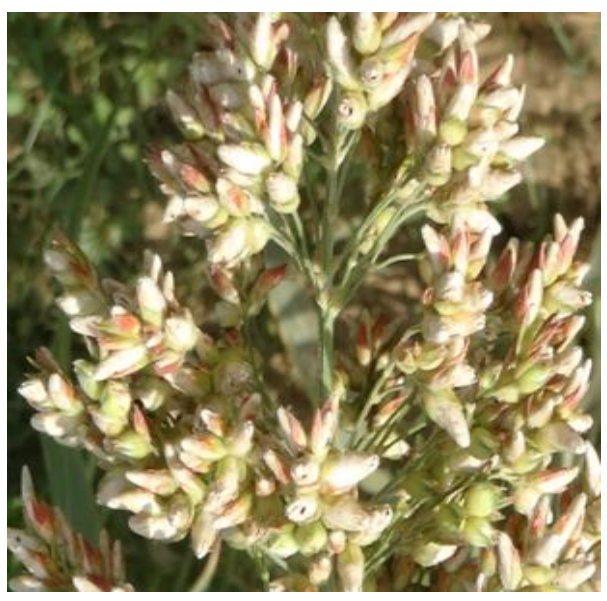

Source: FAO-KSA Survey Report

Management

- The disease is generally kept under control by seed treatment with chemical fungicide. Seed treatment with carboxin @ 2 g/kg is recommended.
- Remove and burn head smut galls before the spores are scattered.

5. Downy Mildew, caused by *Peronosclerospora sorghi*

Pathogen/disease description

Sorghum downy mildew is economically important and widespread in many tropical and subtropical regions of the world where sorghum and maize are grown. The disease has also been observed in the Jazan region of the KSA. The disease is highly destructive due to its systemic nature of infection resulting in death of plants or lack of panicle initiation.

Disease symptoms

- Systemic infection occurs when the young meristematic tissues of the growing seedlings are infected and the symptoms appear as chlorotic foliage and stunted, often resulting in death of seedlings. The first infected leaf shows chlorosis on the lower part of the lamina, which further grows to cover larger part of the leaf. The other leaves on a plant that gets infected subsequently show more chlorosis.
- Under cool and humid weather conditions, the abaxial surface of chlorotic leaves produce abundant spores (conidia) that appear as white, downy growth. As the plant grows, new emerging leaves exhibit parallel stripes of green and white tissue; the white interveinal tissue dies, and leaf shredding occurs.
- The local lesions on foliage are the result of infection by conidia. These appear as stippled, necrotic lesions on leaf blades.

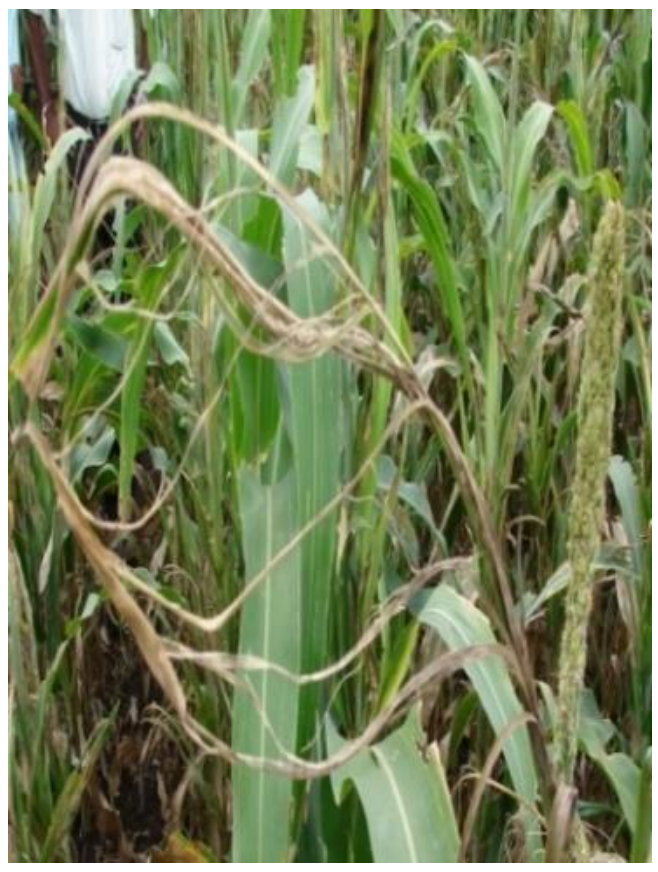

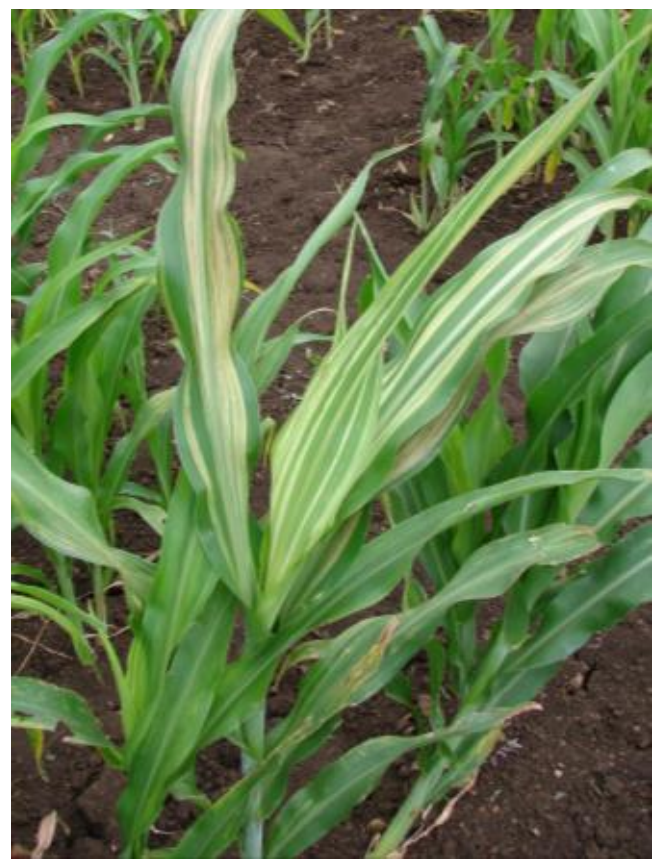

Source: @ICRISAT Information Bulletin No. 76

Management

- Some of the highly resistant lines/varieties are: QL 3, IS 3443, IS 8283, IS 1331, IS 2474, IS 3547, IS 5743, IS 7179, IS 8185, IS 8276, IS 8607, IS 8864, IS 8906, IS 8954, IS 18757, IS 22228, IS 22229, and IS 22230.
- Crop rotation for at least two years is helpful.
- Seed treatment with metalaxyl 35 SD @ 1 g ai/kg seed followed by one spray of metalaxyl 35 WS @ 1.5 ml/l water at 40 days after emergence (DAE) is effective.

6. Crazy top, caused by *Sclerophthora macrospora*

Pathogen/disease description

The disease develops in young plants growing in wet soil and is generally more prevalent in the wetter parts of a field. Oospores present in soil are the main inoculum source of *S. macrospora*; wild and cultivated grasses can also be source of inoculum.

Disease symptoms

- In young plants, the disease produces mottled yellow plant tissue like that of mosaic symptoms caused by some viruses.
- As the disease progresses, leaves become thick and twisted.
- In the infected plants, the heads often do not emerge or are malformed.

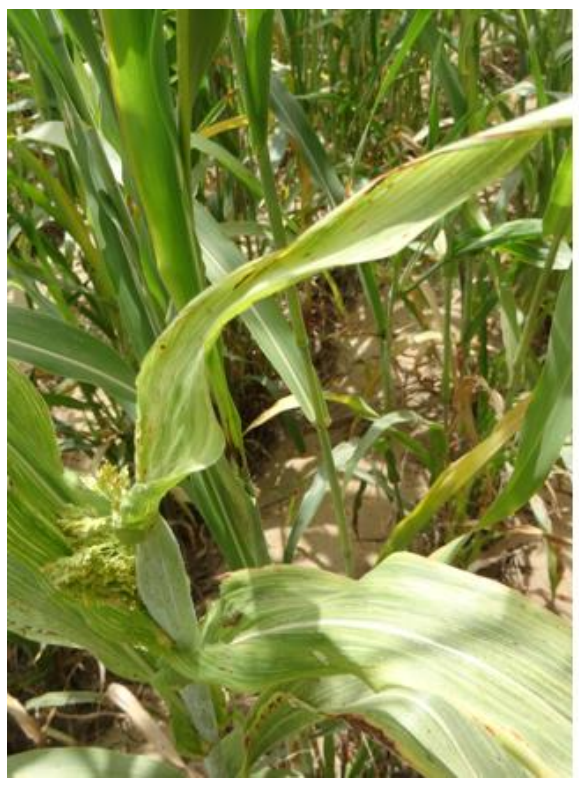

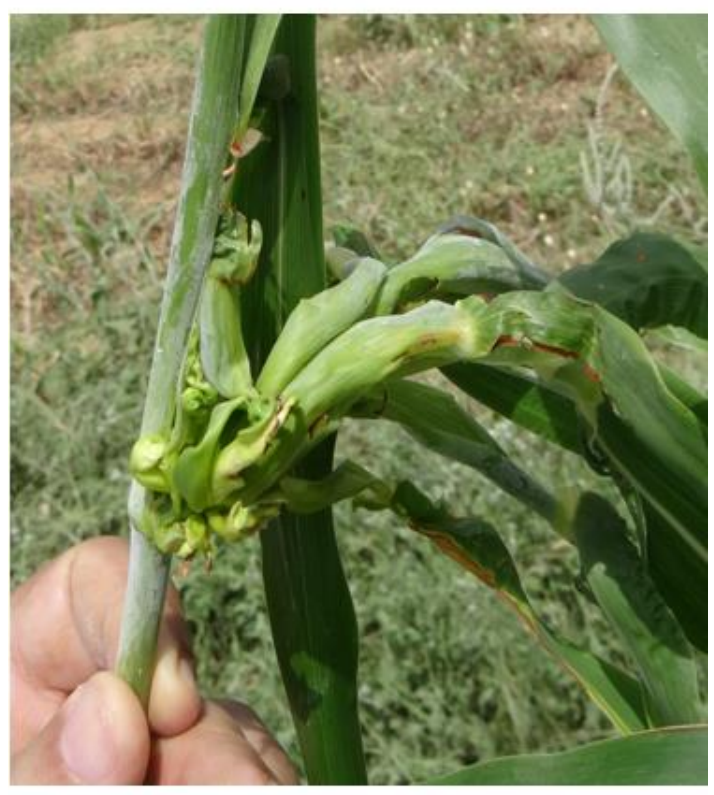

Source: FAO-KSA Survey Report

Management

- Fields frequently subjected to flooding should be avoided. Improving drainage in fields, deep tillage and crop rotations for more than three years are helpful in disease management.
- Seed treatment with metalaxyl at 2 g a.i./kg seed is recommended.

7. Grain Mold, caused by *Several fungal species*

Pathogen/disease description

Grain mold is a major constraint for optimum sorghum grain yield and quality. Early maturing, high yielding hybrids and improved varieties that are grown during the rainy season are more vulnerable to grain mold. The major pathogenic fungi associated with grain mold complex are species of *Fusarium* (*F. thapsinum, F. proliferatum, F. andiyazi, F. nygamai* and *F. verticillioides*), *Curvularia lunata, Alternaria alternata,* and *Phoma sorghina*. Some of the strains of *Fusarium* species are known to produce mycotoxins, such as fumonisins, moniliformin and trichothecenes.

Disease symptoms

- Initial symptoms of grain mold are discoloration of grains due to infection and colonization by mold fungi. Grain discoloration varies from light whitish, pinkish, grayish, to shiny black depending on infection and colonization by individual fungal species. Often grains are colonized by multiple fungi. In severe cases, grains turn completely black.
- In case of severe infection at anthesis, grain development is affected resulting in chaffy florets or small grains.
- Grain mold infection leads to reduced kernel development, discoloration of grain, colonization and degradation of endosperm and germ, decreased grain density, decreased germination and seedling vigor.

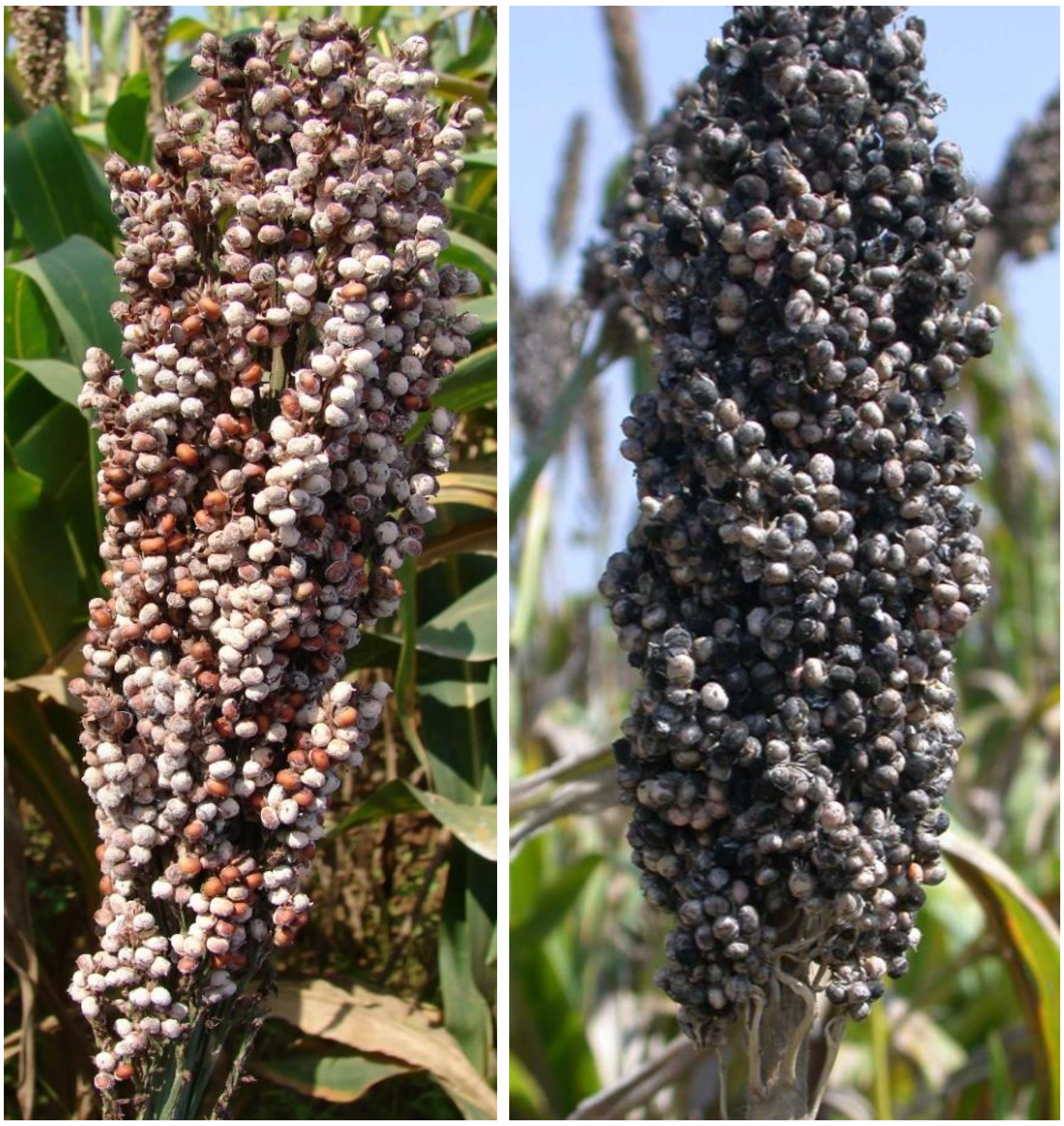

Source: @ICRISAT Rajan Sharma and RP Thakur

Management

- Host plant resistance: Several resistant accessions (IS 2815, IS 21599, IS 10288, IS 3436, IS 10646, IS 10475 and IS 23585) have been used in breeding programs to develop restorer lines, varieties and hybrid parents.
- Harvesting at physiological maturity and drying. Maintain grain moisture level up to 10% in storage.
- Spray propiconazole 25 EC @ 0.1% at flowering.

8. Head blights, caused by *Fusarium* and *Colletotrichum* spp.

Pathogen/disease description

Head blight is a panicle disease of sorghum. It is caused by many of the same fungi responsible for grain mold primarily *Fusarium* and *Colletotrichum* spp. The disease is described differently due to different symptoms. It is considered as a minor disease with sporadic incidence in major sorghum growing areas of the world.

Disease symptoms

- Symptoms of the disease include reddening of the pith tissues of the peduncle and the rachis or rachis branches.
- The initial infection takes place at the lower part of the panicle that spreads upward.
- High humidity at the time of flowering favors infection and colonization.
- Sometimes, small black reproductive structures (acervuli) with spines (setae) are observed on the panicle indicating infection and disease caused by *Colletotrichum*, the same fungus responsible for anthracnose.

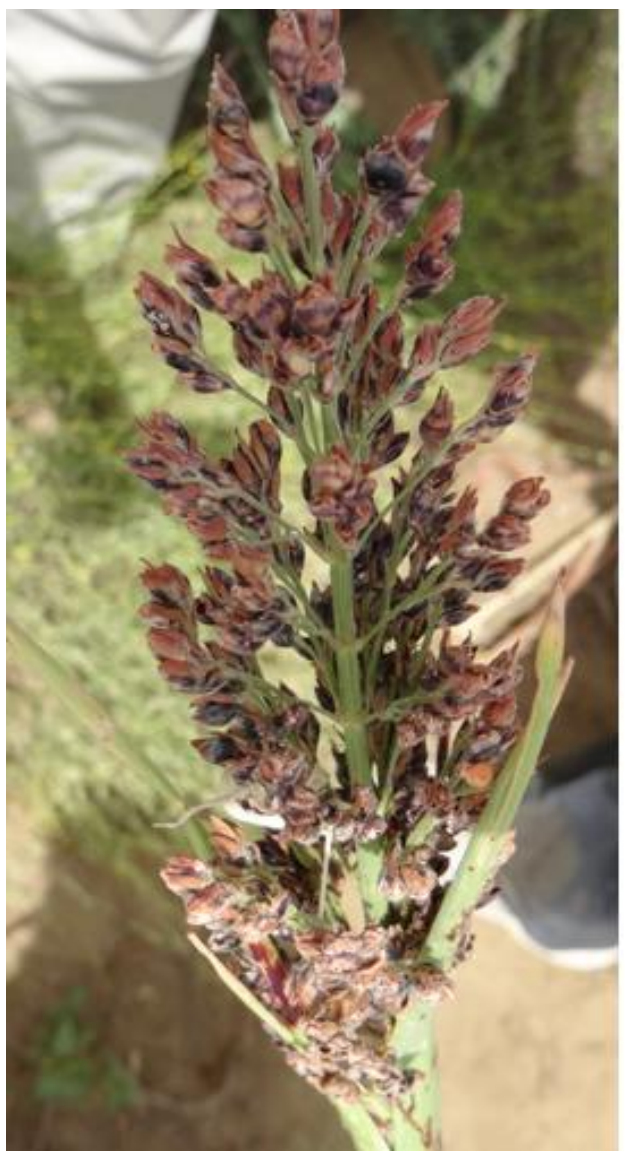
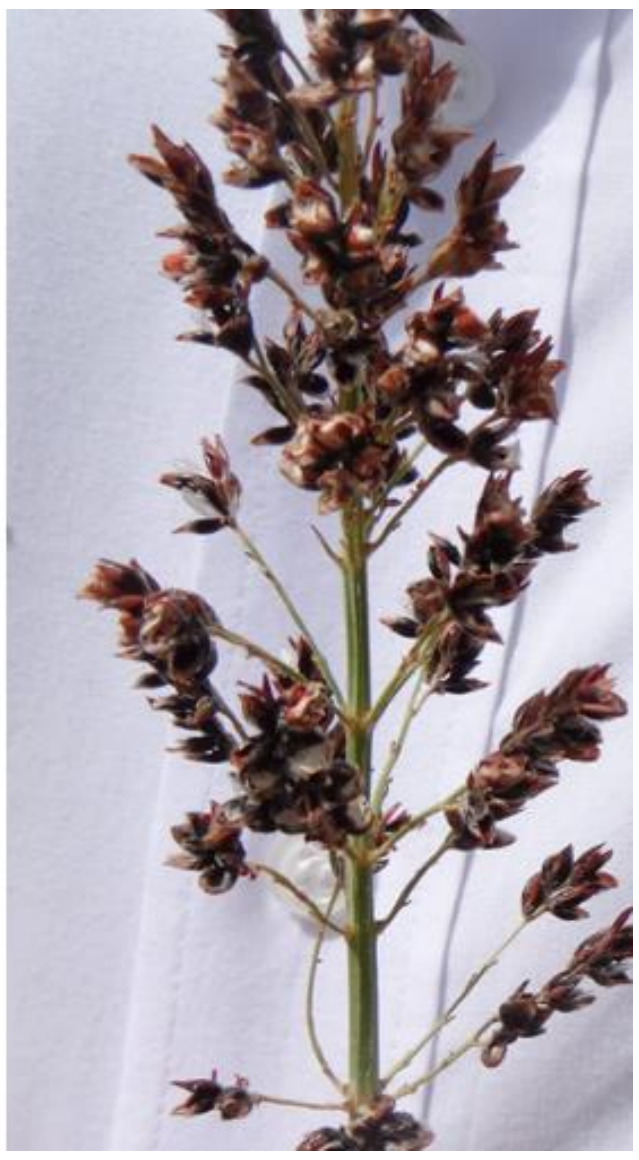

Source: FAO-KSA Survey Report

Management

- Host plant resistance is the best management option. Elite sorghum breeding lines need to be screened in KSA for resistance against head blight.
- Harvesting at physiological maturity.

9. Sooty stripe, caused by *Ramulispora sorghi*

Pathogen/disease description

Sooty stripe is an important leaf disease in many sorghum-producing regions. It can cause serious yield losses in Africa, India, and the United States. The pathogen also infects Columbus grass, Johnson grass, and Sudan grass.

Disease symptoms

- The initial symptoms appear as small non-diagnostic lesions on the lower leaves. These lesions elongate and form the typical diagnostic oval-elongate sooty stripe lesions surrounded by a prominent reddish-brown lesion margins with yellow (chlorotic) halos.
- The production of numerous black microsclerotia in the center of mature lesions gives the lesions a sooty appearance.
- Lesions may expand and result in leaf blight.
- The disease progresses under favorable weather conditions and lesions may occur on all leaves of the plant.

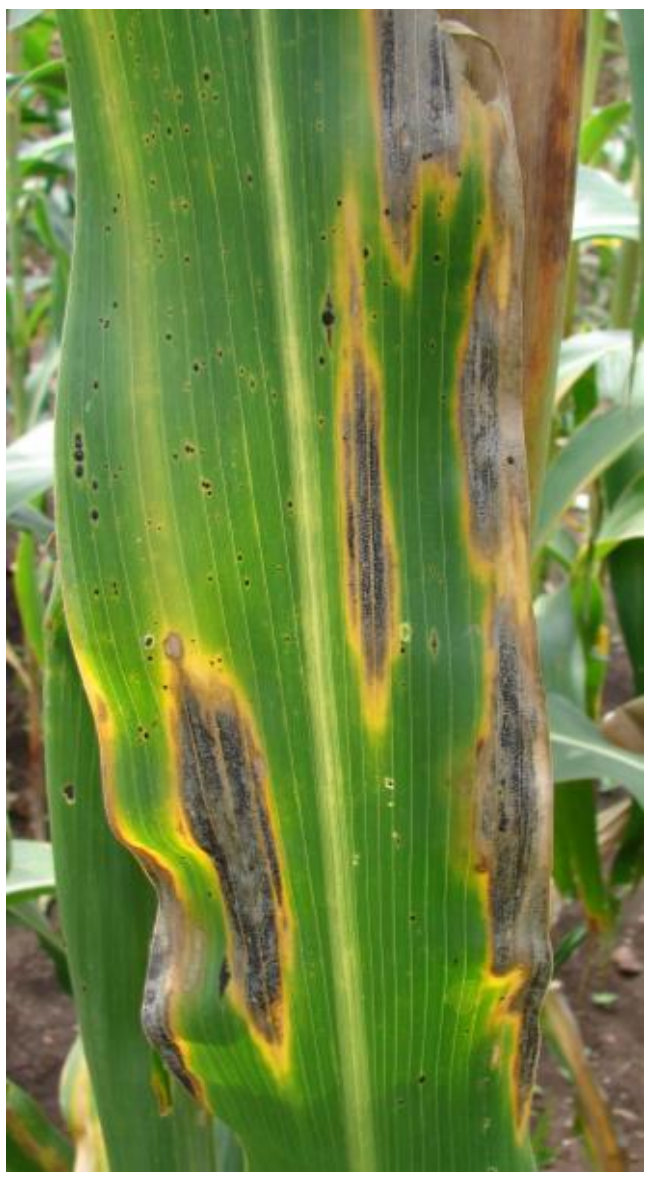

Source: @ICRISAT RP Thakur

Management

- Crop rotation and removal of crop residues.
- Fungicides registered in KSA for sorghum can be tested for their efficacy against sooty stripe.

10. Banded leaf and sheath blight , caused by *Rhizoctonia solani*

Pathogen/disease description

Rhizoctonia sheath blight is one of the destructive diseases affecting sorghum, and other tropical cereals such as maize and rice growing in high rain fall conditions. The casual fungus is soil borne and survives between cropping seasons on crop like corn, rice, mung bean, other plants and weeds as mycelium and in the soil as sclerotia.

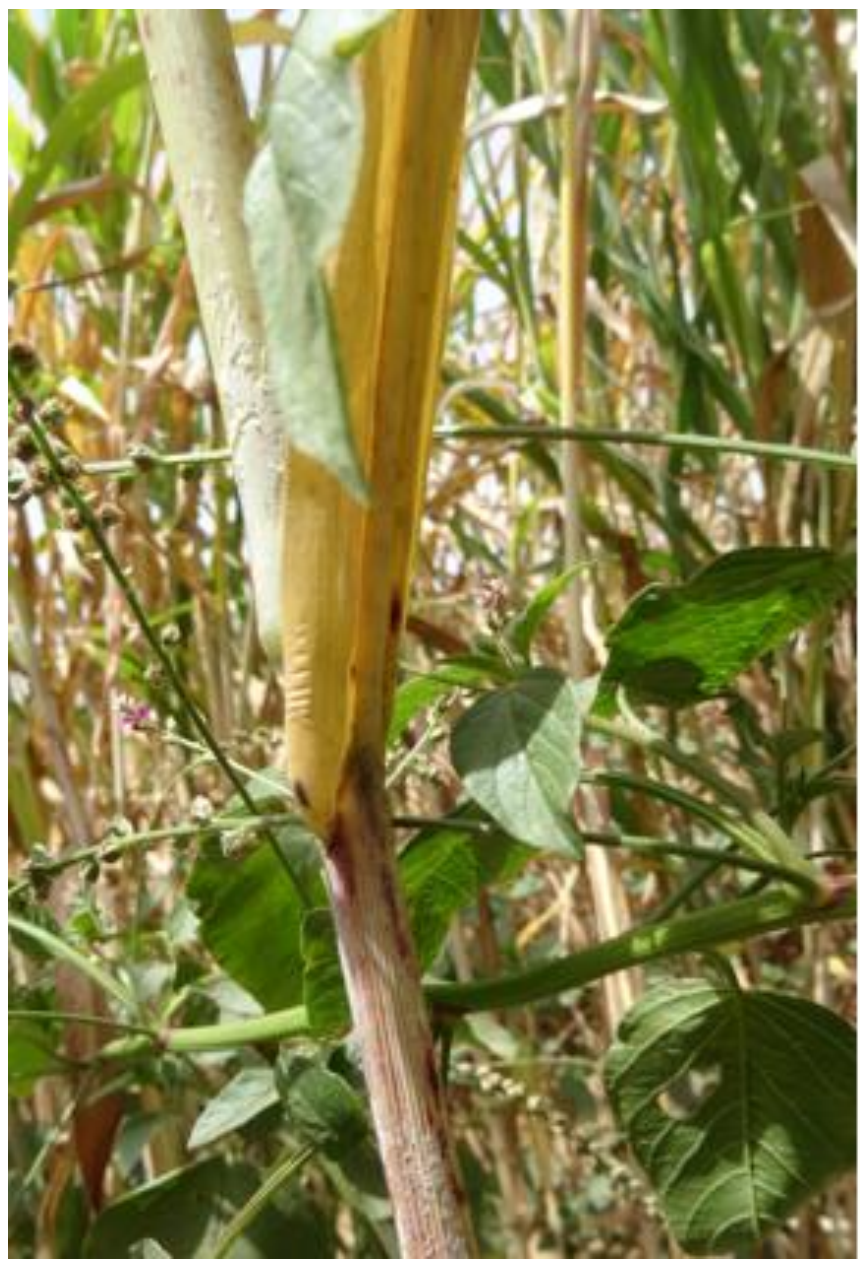

Source: FAO-KSA Survey Report

Disease symptoms

- Normally, the disease starts from the basal leaf sheaths and spreads upward reaching as high as the panicle.
- The infected hosts develop water soaked grey-green leaf and sheath lesions which becomes necrotic and give the infected tissues a banded appearance.
- Mycelial strands and fluffy white to light brown mycelial mats (sclerotia) of the casual fungus can be clearly seen on the surface of the lesions.

Management

- Several of species of *Trichoderma, Gliocladium,* and *Laetisaria*, bacteria (*Pseudomonas* sp., *Bacillus subtilis*), and nematodes (*Aphelenchus avenae*) have been reported to parasitize *Rhizoctonia* species and can be tested in KSA.
- Cultural practices like stripping of the second and third leaf sheaths from the ground level at 35-40 days old crop has been found effective in maize.
- Selection of a well-drained field and planting on raised beds, optimum plant population, application of farmyard manure prior to planting are important cultural aspects to manage the disease.

11. Gray leaf spot, caused by *Cercospora sorghi*

Pathogen/disease description

Gray leaf spot, or rectangular leaf spot or Cercosporiosis, is a disease of medium importance as it appears late in the crop. It occurs in most of the sorghum-growing regions and has also been observed in the Jazan region of the KSA. The disease spreads through seed and by wind. Warm temperature, frequent rain, and high humidity favor the development of the disease.

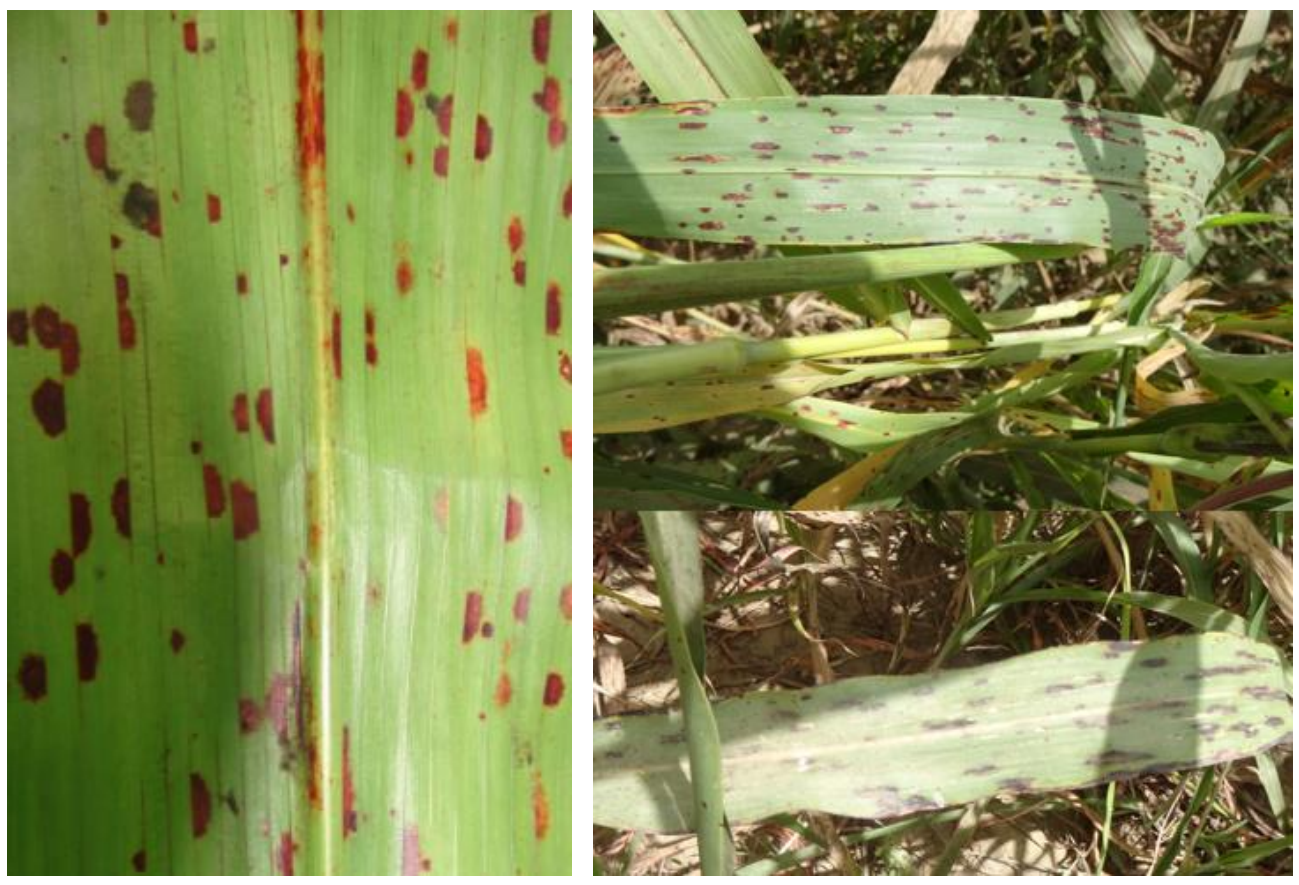

Source: FAO-KSA Survey Report

Disease symptoms

- Initial symptoms include small red spots on leaves which then enlarge to form narrow rectangular lesions delimited by veins.
- As the disease progresses, the spots elongate and heavy sporulation occurs, indicated by velvety gray spore masses on both the surfaces of leaf.

Management

- Crop rotation, use of healthy seeds or resistant cultivars, avoiding overcrowding, proper field sanitation, removal of infected plant debris, etc. are helpful in disease management.
- Propiconazole 25% EC @ 1 ml/l water can be used.

12. Goss's Wilt, caused by *Clavibacter michiganensis*

Pathogen/disease description

The disease is caused by a bacterium which primarily infects leaves that have been wounded. The disease was observed in the Jazan region of the KSA with about 10% disease severity (FAO-KSA survey report). The pathogen infects several hosts including maize, grain sorghum, green foxtail, barnyard grass, shattercane, large crabgrass, and others. The disease can spread from plant to plant and disease development is favored by warm climatic conditions.

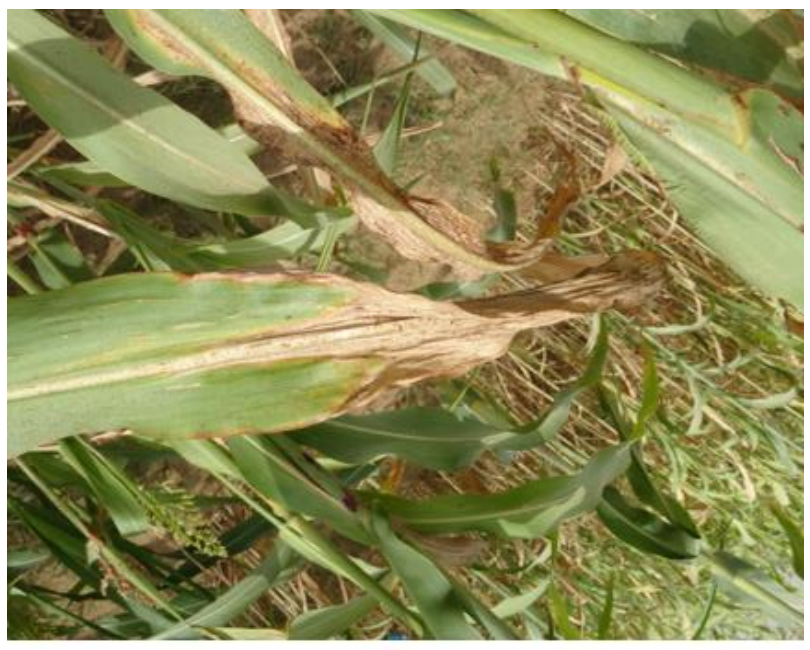

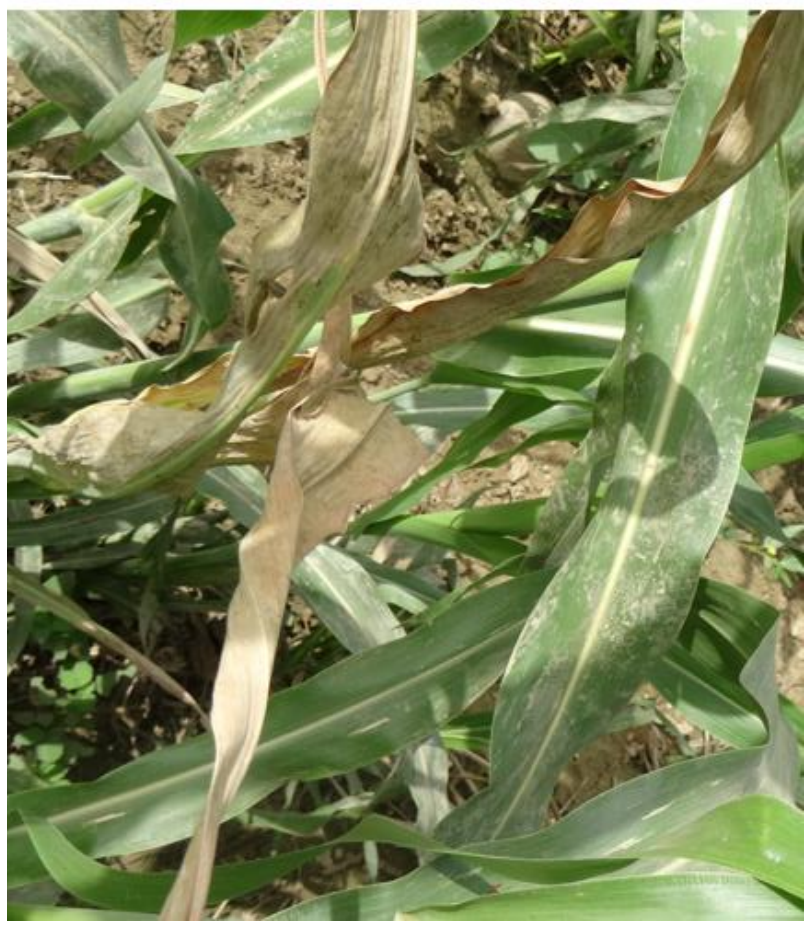

Disease symptoms

- The primary symptoms appear on leaves as elongated tan lesions with irregular margins extending parallel to the veins.
- Dark, water-soaked spots ('freckles') develop within the margin of the expanding lesions. When these "freckles" are observed against the light, transparent spots can be seen.
- Shiny patches of dried bacterial ooze can be observed on the lesions.
- The seedling blight phase of this disease may cause wilting and death of seedlings.

Management

- Cop rotation between broadleaf crops and small grains other than sorghum and maize as they are primary hosts for Goss's wilt.
- Deep tillage practices that bury the crop residues may speed up decomposition thus reduce the inoculum level.

Source: FAO-KSA Survey Report

13. Mosaic virus

Pathogen/disease description

Maize mosaic virus (MMV) is an economically important sorghum virus prevailing in semi-arid tropics of the world. The MMV-S infecting sorghum is serologically related to MMV and belongs to subgroup plant Rhabdovirus with four major proteins. MMV-S is transmitted by a delphacid planthopper *Peregrinus maidis* which is one of the major pests on sorghum.

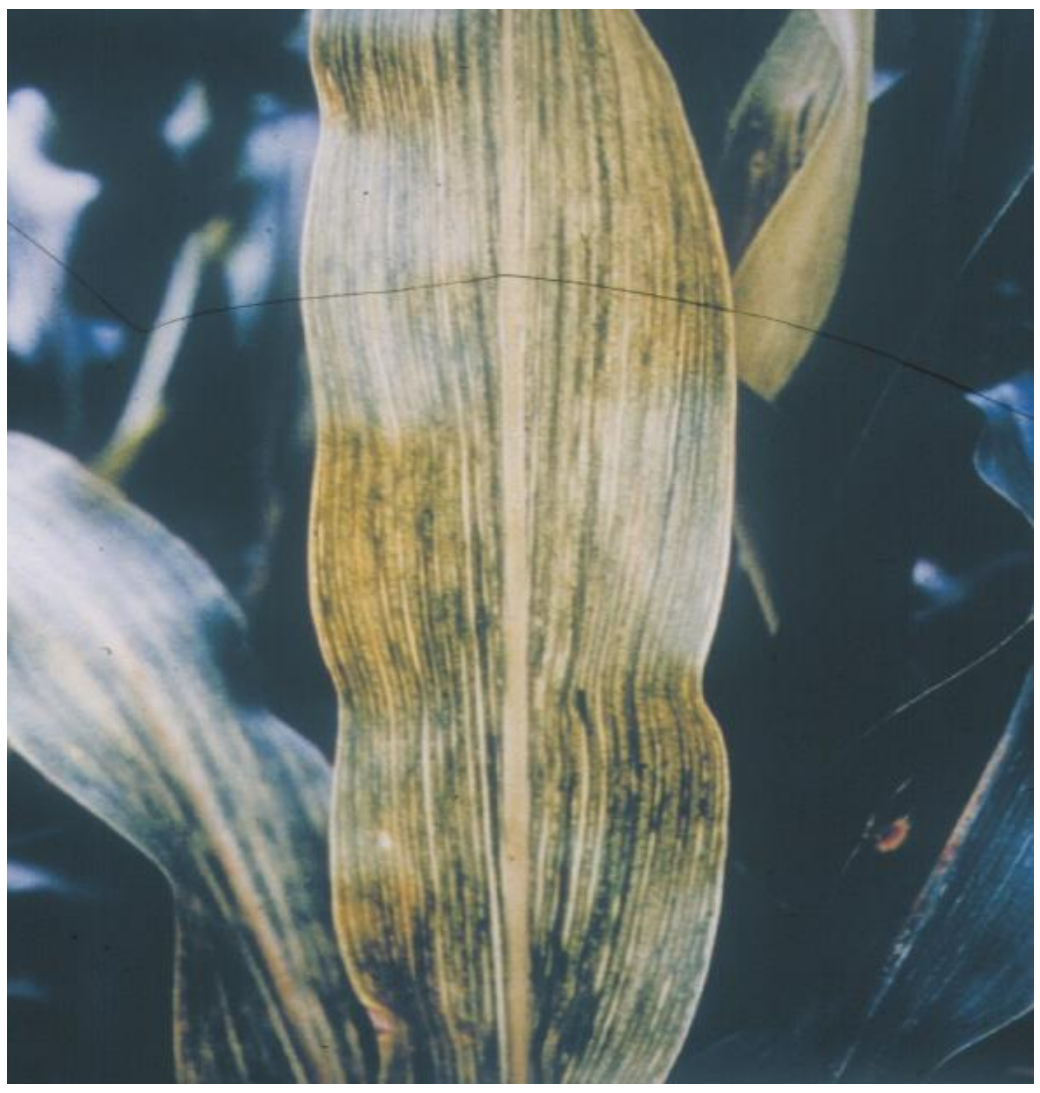

Source: @ICRISAT RP Thakur

Disease symptoms

- The symptoms include the appearance of fine continuous chlorotic or broken streaks between the veins on leaves that may become necrotic as the disease progresses.
- Severe stunting of plants with shortened internodes and fewer seed setting on panicles.
- Some of the symptoms of nutrient deficiency in sorghum and diseases such as downy mildew also resemble the symptoms of viral diseases. Therefore, symptoms diagnosis should be confirmed by other techniques.

Management

- Eradication of weed hosts, removal of ratoons, rouging of symptomatic plants, adjustment of sowing time, when possible, avoidance of the vector and chemical control of vector with acetamiprid 20% SP 0.5 g/l water are useful in the management of MMV in sorghum.

Insect pests of sorghum

1. Shoot fly, *Atherigona vavia soccata* (Muscidae: Diptera)

Pest description

- Shoot fly females lay cigar shaped eggs on the lower leaf surface at the 1 to 7 leaf stage.
- **Eggs** - White, cylindrical, distal slightly flattened; **Adult** - Whitish gray fly.

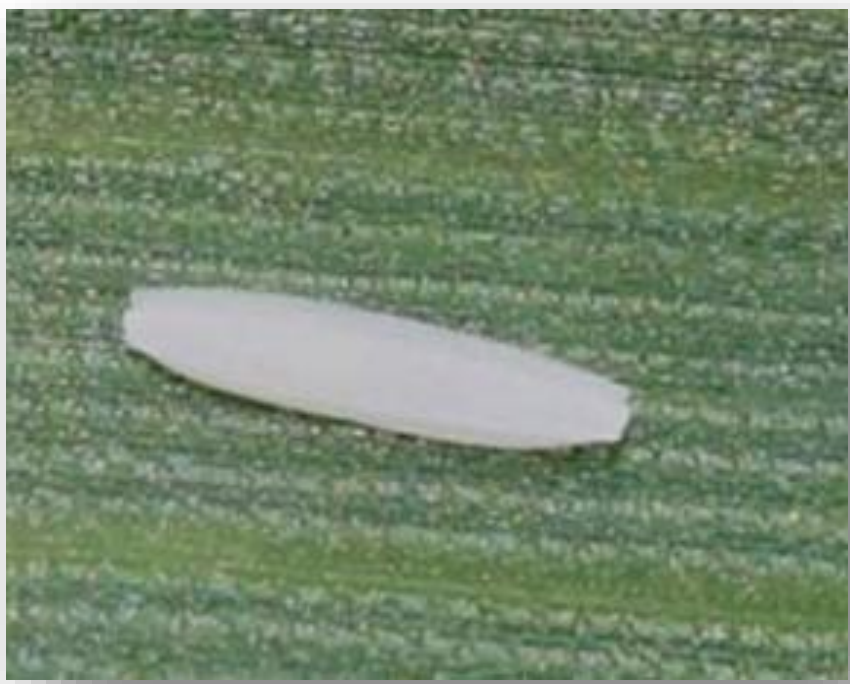
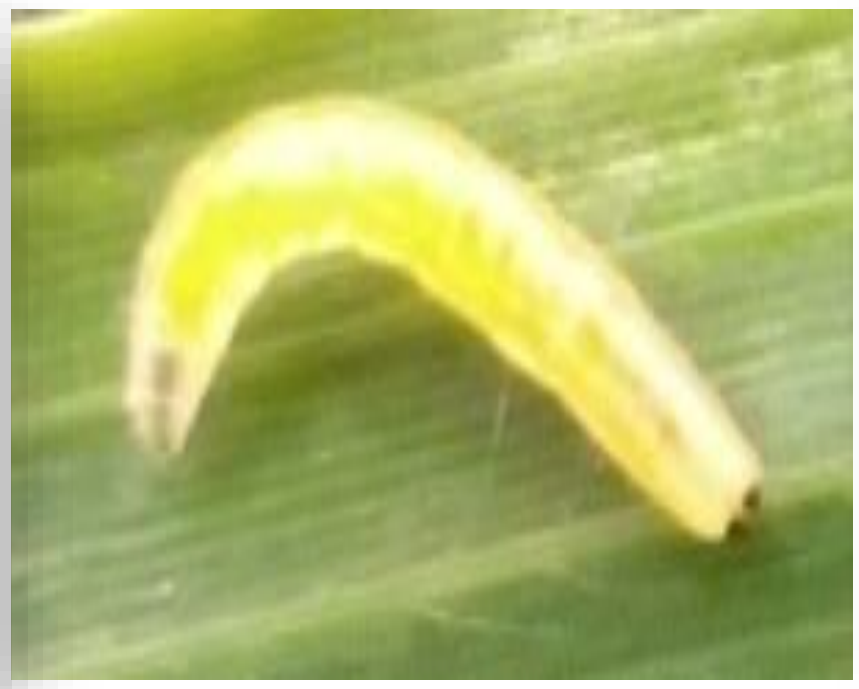
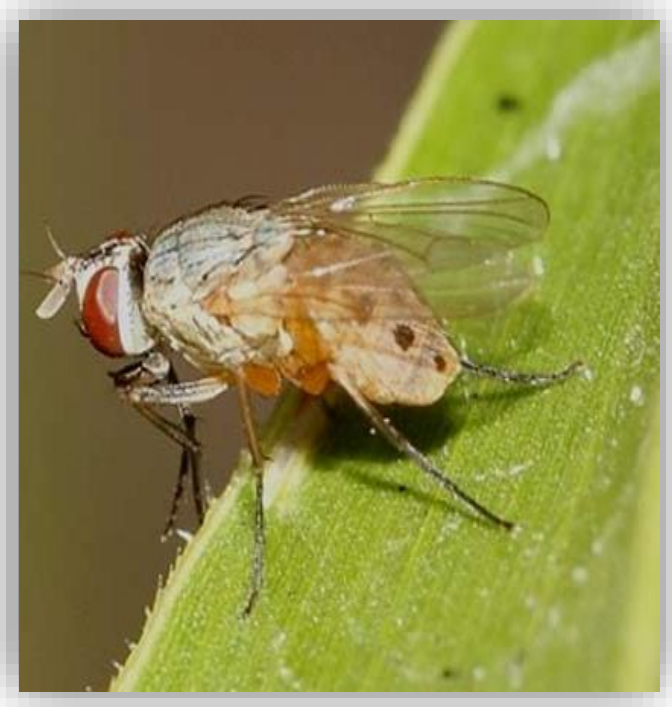
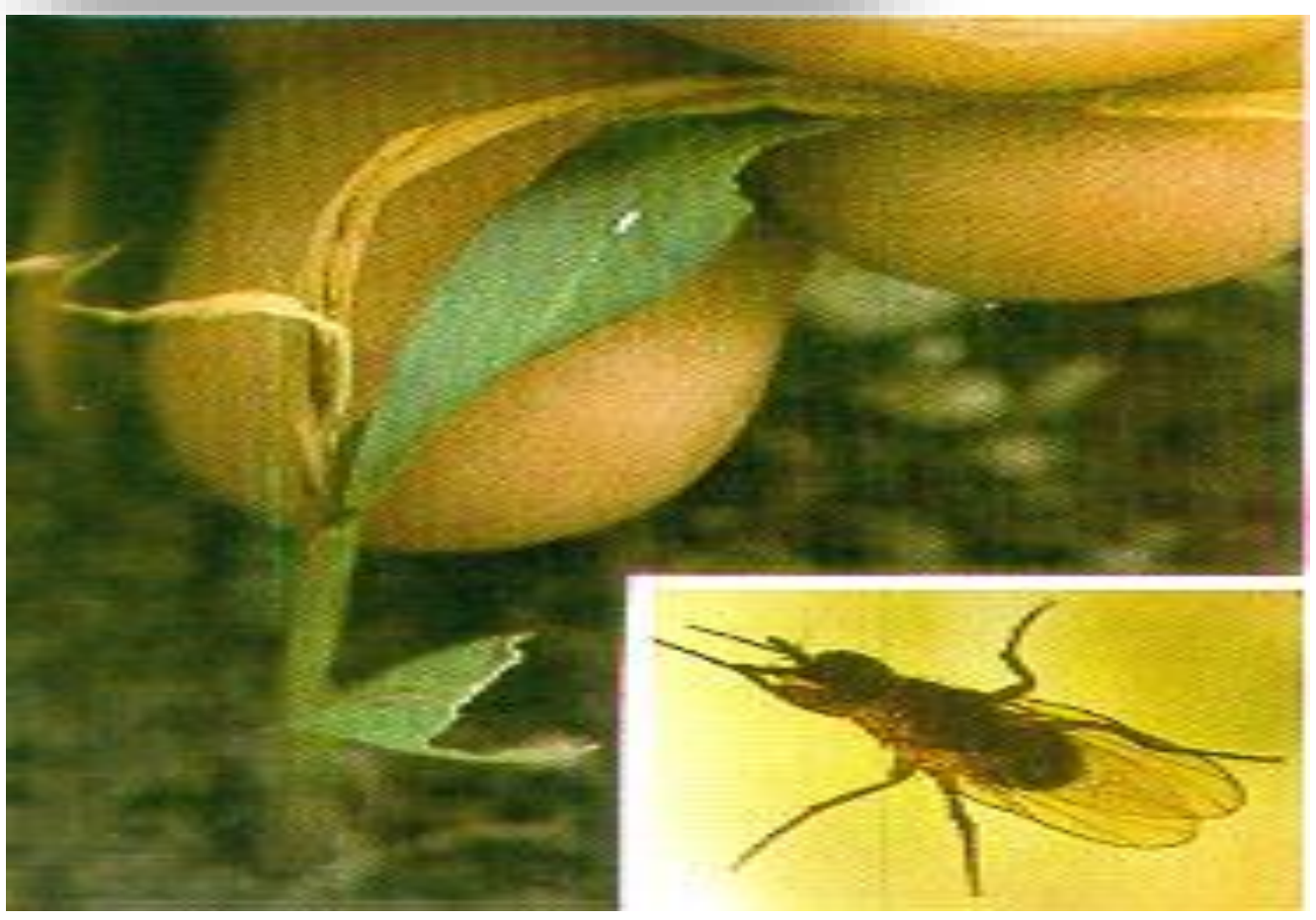
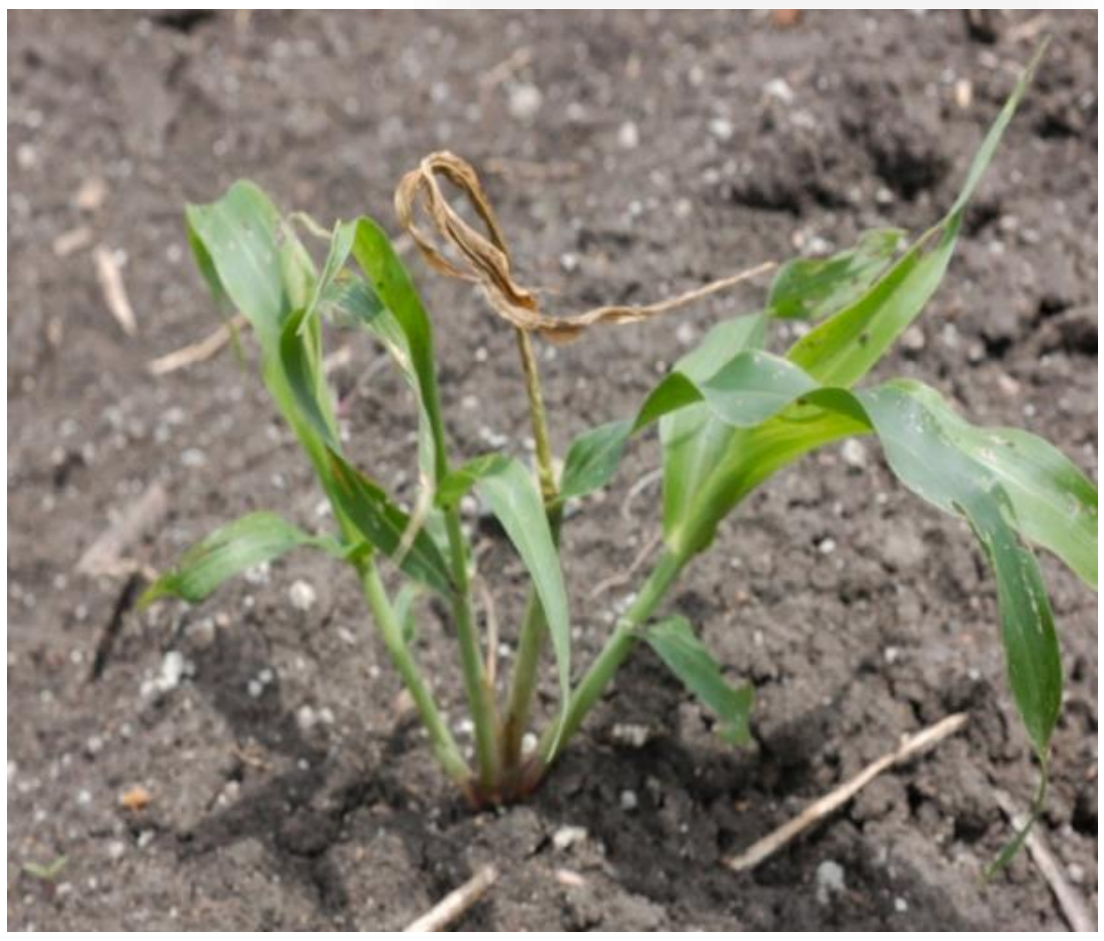

Source: @ICRISAT HC Sharma

Damage symptoms

- The maggot bores inside the stem and cuts the growing point.
- Central shoots dry up and produce “dead heart” symptom.
- The infested plant produces side tillers.

Management

- *Trichogramma chilonis* and *T. simmondsi* are egg parasitoids and several species of spiders are important predators of eggs of shoot fly and could be reared and used as natural enemies.
- In case of early sowing, maintain a high seed rate.
- Pulling out alternate weed host such as barnyard grass.
- Seed treatment with acetamiprid 70 WDG @ 5 g/kg of seeds.
- Under late plantings, shoot fly can be controlled with the application of imidacloprid 2 GR @ 450 g/ha at the time of planting in the seed furrows. Alternatively, shoot fly damage can be minimized by spraying chlorantraniliprole 20% SC @ 0.3 ml/l water at 7 and 14 days after the emergence of seedlings.
- For monitoring, set up the low-cost fish meal trap @ 12/ha till the crop is 30 days old.

2. Stem borer, *Chilo partellus* (Crambidae: Lepidoptera)

Pest description

Eggs - Scale-like flat oval eggs in batches on the under surface of leaves near the midribs; **Larva** - Yellowish brown with a brown head and prothoracic shield; **Adult** - Moth is medium sized and straw colored.

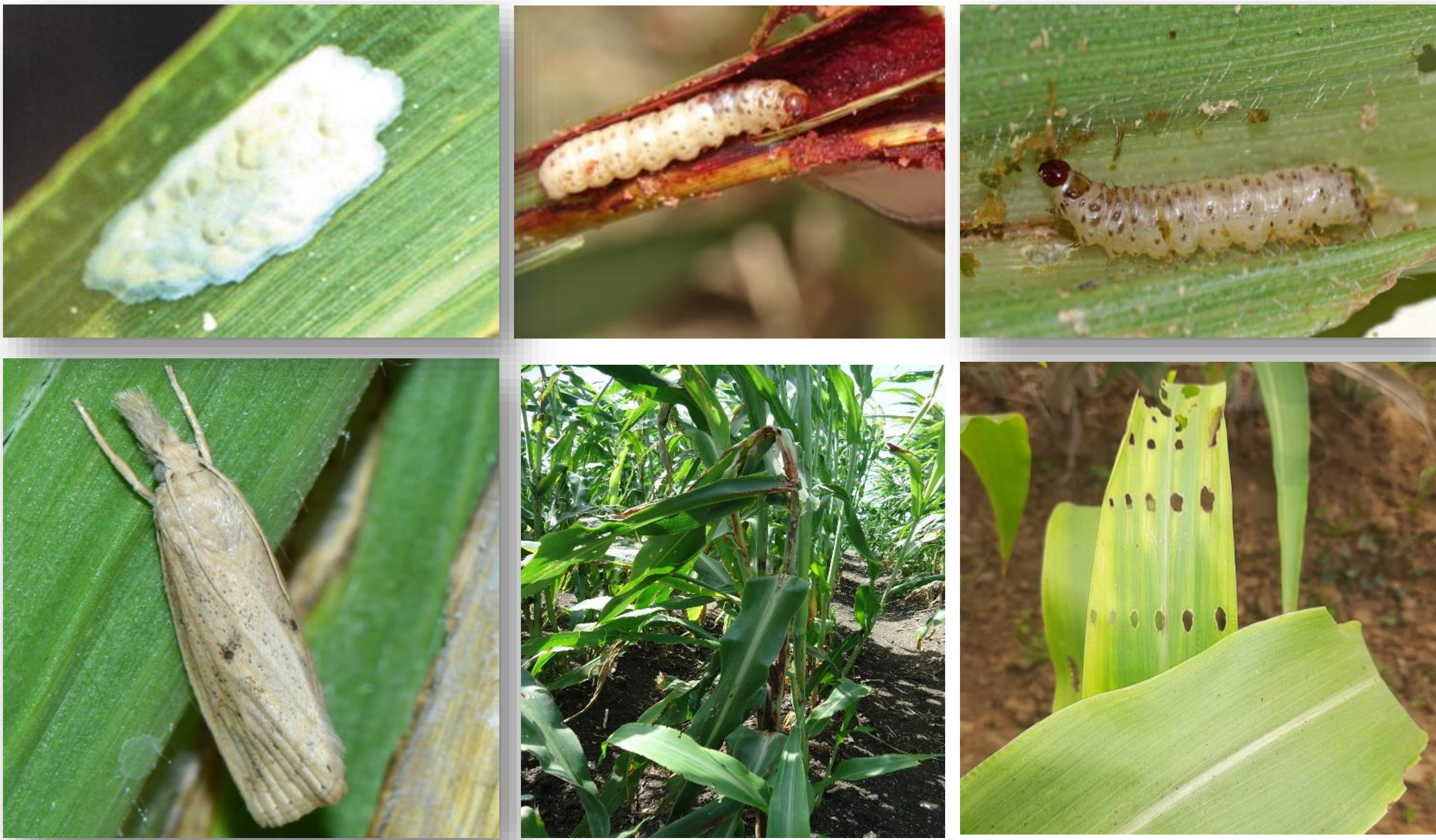

Source: FAO-KSA Survey Report and @ICRISAT HC Sharma

Damage symptoms

- Withering and drying of central shoot -“dead heart”.
- Red mining in the midrib.
- Bored holes visible on the stem near the nodes.
- Tender folded leaves have parallel “shot holes”.
- Affected parts of stem may show internal tunneling of caterpillar holes.
- Tunneling of earheads results either in its breakage, or complete/ partial chaffy earheads affecting the grain development.

Management

- Set up light traps till mid night to monitor, attract and kill adults of stem borer, grain midge and earhead caterpillars.
- *Trichogramma chilonis* and *Cotesia chilonis* are egg and larval parasitoid, respectively, and *Chrysoperla zastrowi* is a predator of stem borer that can be used to manage stem borer.
- Emamectin benzoate 5 SG @ 0.4 g/l water or imidacloprid 2 GR @ 450 g/ha can be used.
- Sorghum genotypes ICSV 700, ICSV 705, IS 18551 and ICSV 93046 have tolerance to stem borer.

3. Fall armyworm, *Spodoptera frugiperda* (Noctuidae: Lepidoptera)

Pest description

Larvae with 4 black spots arranged (in square shape) on 8th and 9th abdominal segment and inverted "Y" shaped mark on head. Male adult moth's forewings are brown with triangular white spots whereas, females' forewings are grayish brown.

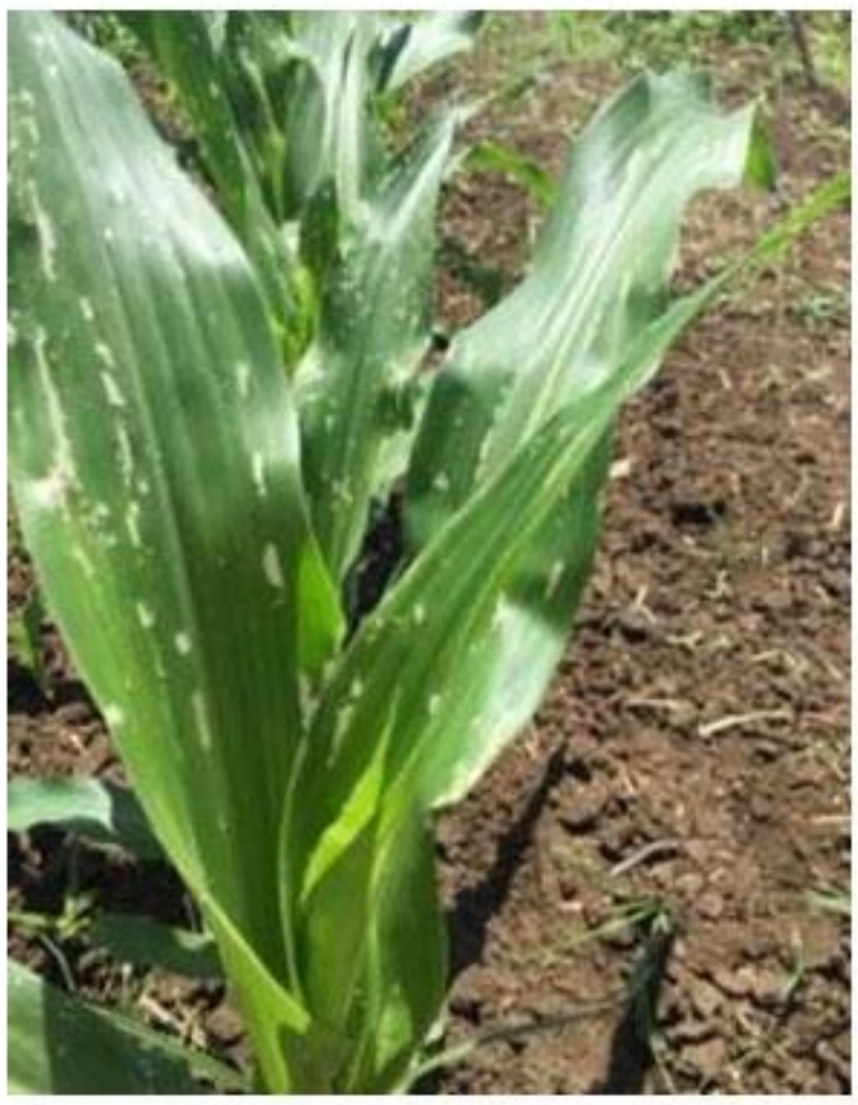

Scraping of leaf

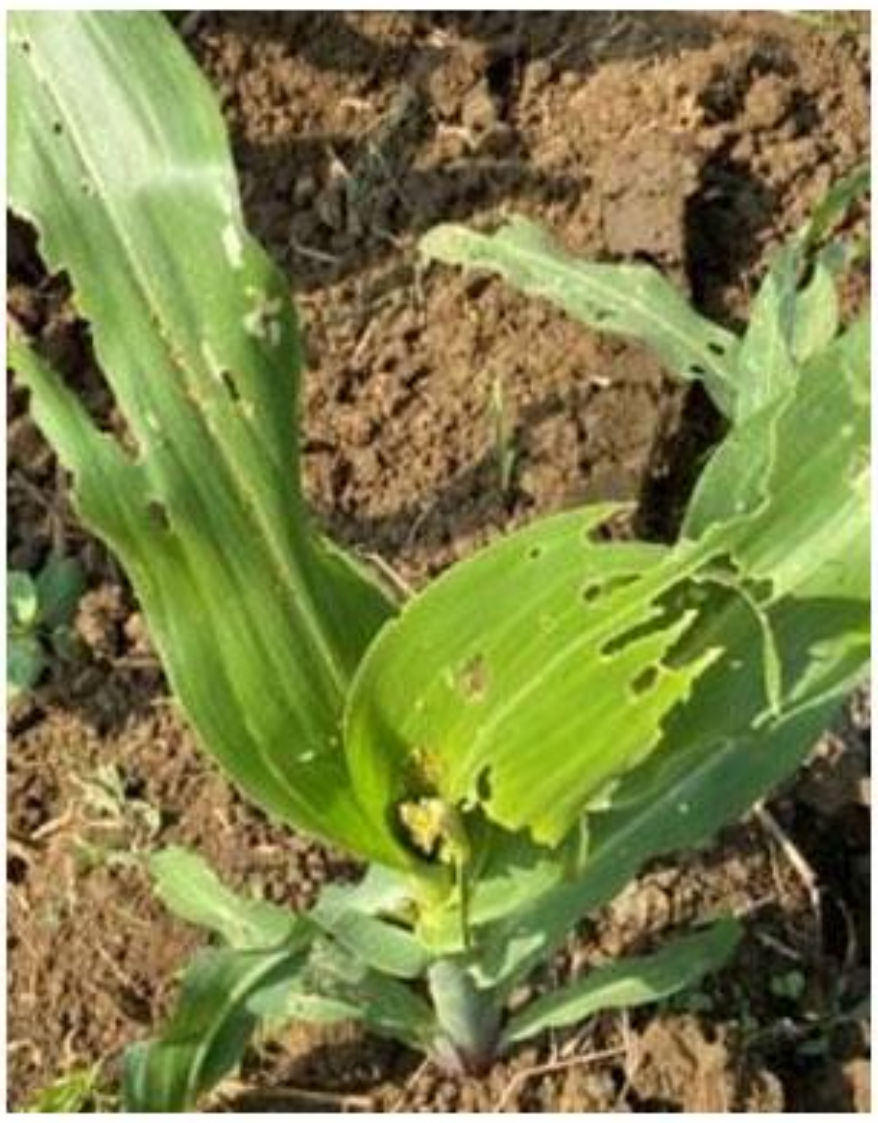

Extensive foliage damage

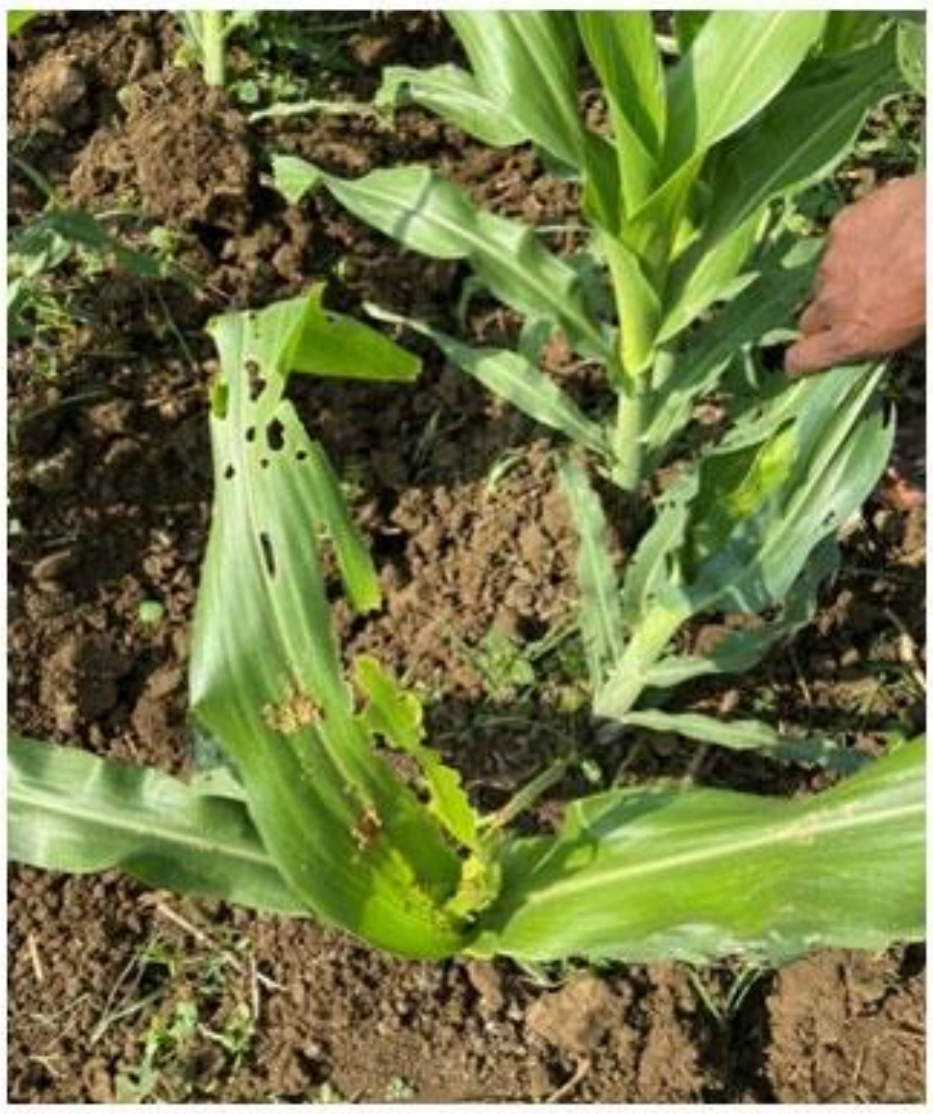

Whorl damage with excreta

Source: @ICRISAT Jagdish Jaba

Damage symptoms

- Scrapping of leaf surface by young larvae.
- The damage by late instars (4th instar) results in extensive defoliation of leaves and the presence of large amounts of fecal pellets in whorls.

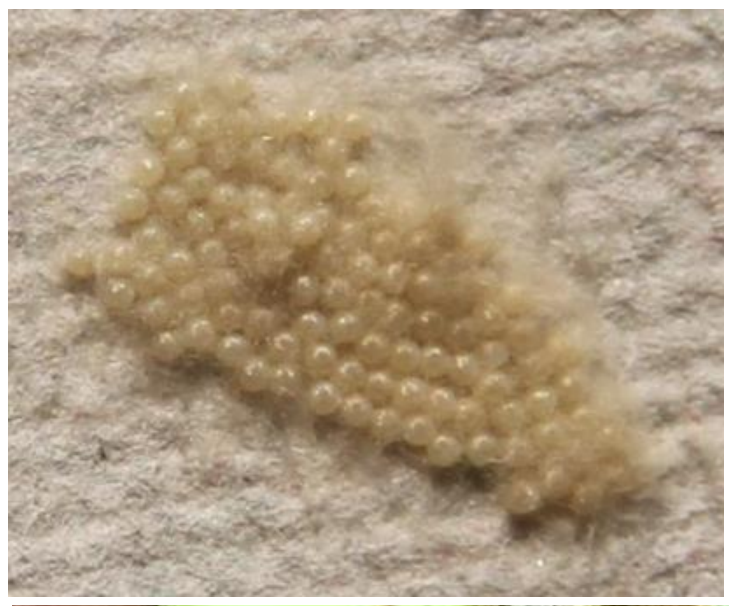

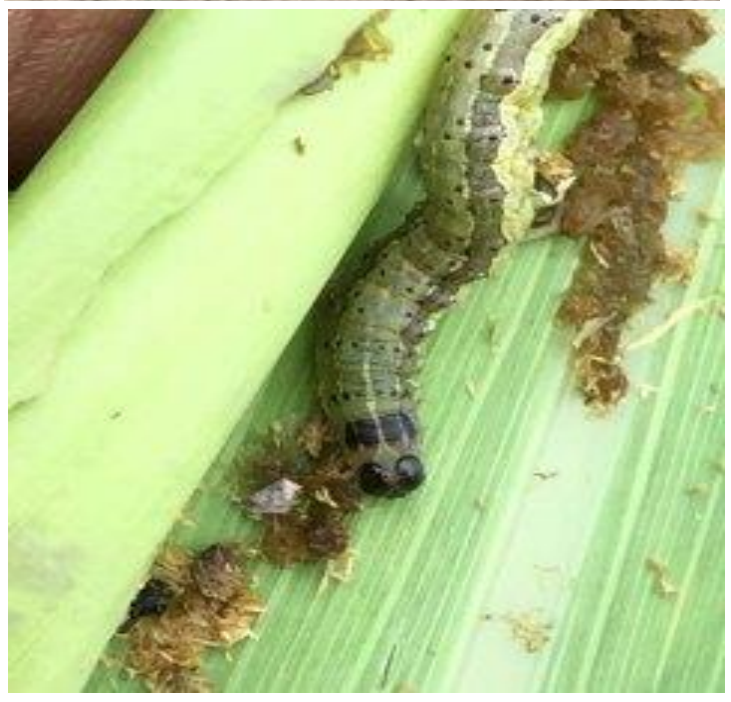

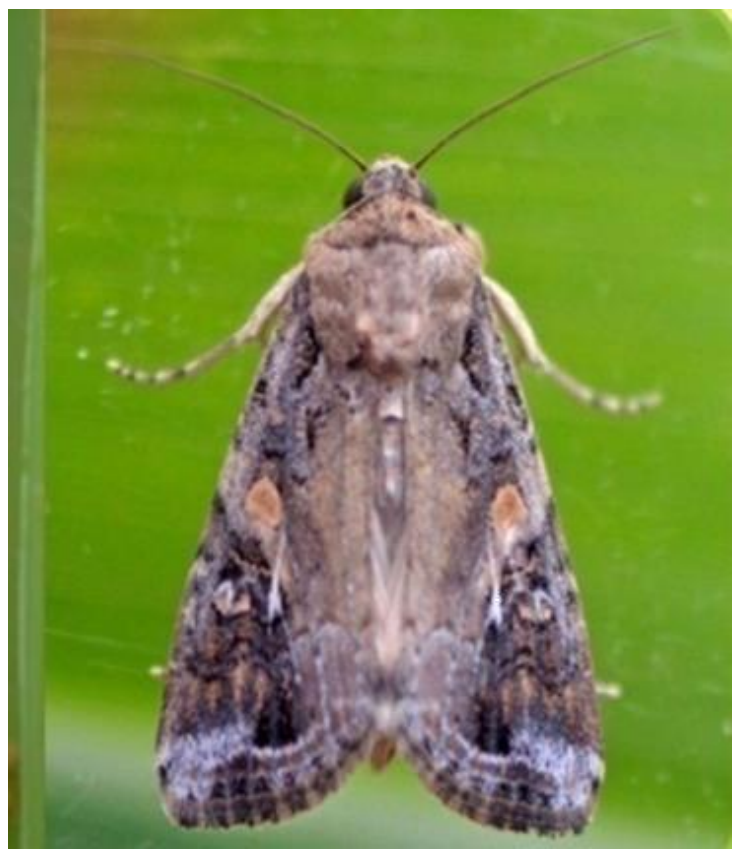

Source: @ICRISAT Jagdish Jaba

Management

- Critical period to protect the crop is up to 50-60 days after seedling emergence.
- Seed treatment with cyantraniliprole-10% OD @ 4 ml/kg seed is reported to offer protection for 2-3 weeks after germination.
- Scouting and assessment of the damage and existence of natural enemies.
- Mass trapping: Pheromone traps @ 25 traps/ha.
- *Telenomus remus* and *Trichogramma* spp. are egg parasitoids and ladybird beetles and ground beetle feed on young caterpillars which can be used as natural enemies.
- Azadirachtin (5ml/l): Ovipositional deterrent.
- Other effective pesticides are emamectin benzoate 5 SG @ 0.4 g/l of water or chlorantraniliprole 20% SC @ 0.3 ml/l water.

4. Aphids, *Rhopalosiphum maidis/Melanaphis sacchari* (Aphididae: Hemiptera)

Pest description

- ***Rhopalosiphum maidis:*** The aphid is dark bluish-green and slightly ovate with black legs.
- ***Melanaphis sacchari:*** The sugarcane aphid is yellow to buff colored.

Damage symptoms

- Yellowing of leaves.
- Colonies of aphids found in central leaf whorl.
- The aphids secrete honeydew, which falls on leaves and on the ground on which sooty molds grow.

Management

- Yellow sticky traps @ 15-20 traps/ha can be used for the effective control of aphids in sorghum plants.
- *Aphelinus nigritus* is an effective parasitoid and most of the ladybird beetle species are general predators.
- Avoid applying excessive doses of nitrogenous fertilizers.
- Spraying acetamiprid 20% SP 0.5 g/l water controls the aphids.
- Sorghum cultivars IS 40618, ICSV 12001, ICSV 12002, ICSV 745, ICSV 700, ICSV 708 have tolerance to aphids.

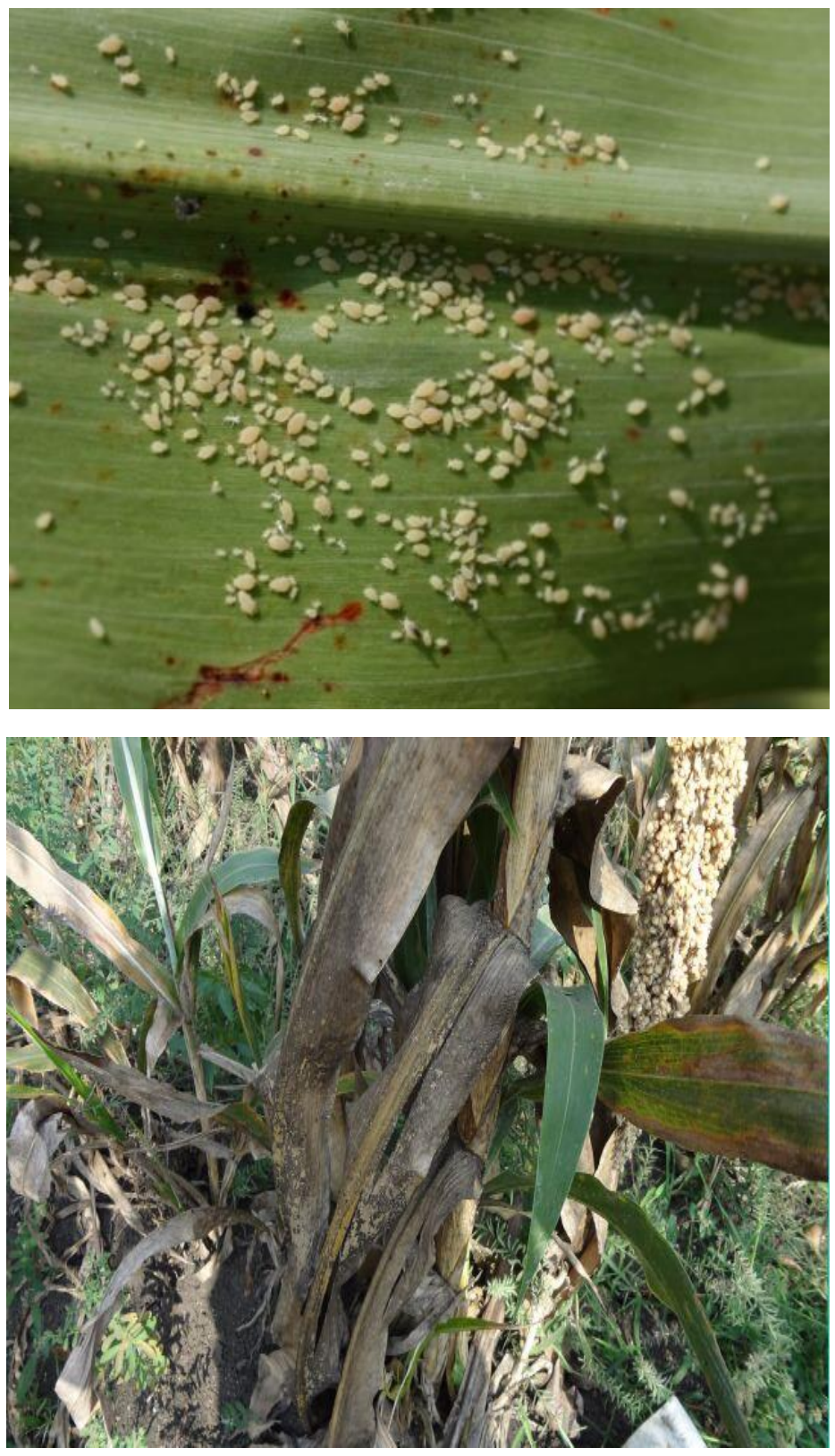

Source: @ICRISAT HC Sharma

5. Earhead bug, *Calocoris angustatus* (Miridae: Hemiptera)

Pest description

- **Eggs -** Blue cigar shaped , laid under the glumes or into the middle of the florets.
- **Nymphs -** Slender, green in color.
- **Adults -** Male is green in color and female is green with a brown margin.

Damage symptoms

- Nymphs and adults suck the juice from within the grains when they are in the milky stage.
- Grains shrink and turn black in color and ill filled (or) chaffy.
- Presence of large number of nymphs and adults are seen on the earhead.

Management

- Apply acetamiprid 20 %SP @ 0.5g/l water at 3rd and 18th day after panicle emergence or spray twice with 5% neem seed kernel extract at 10% heading and after 9 days.

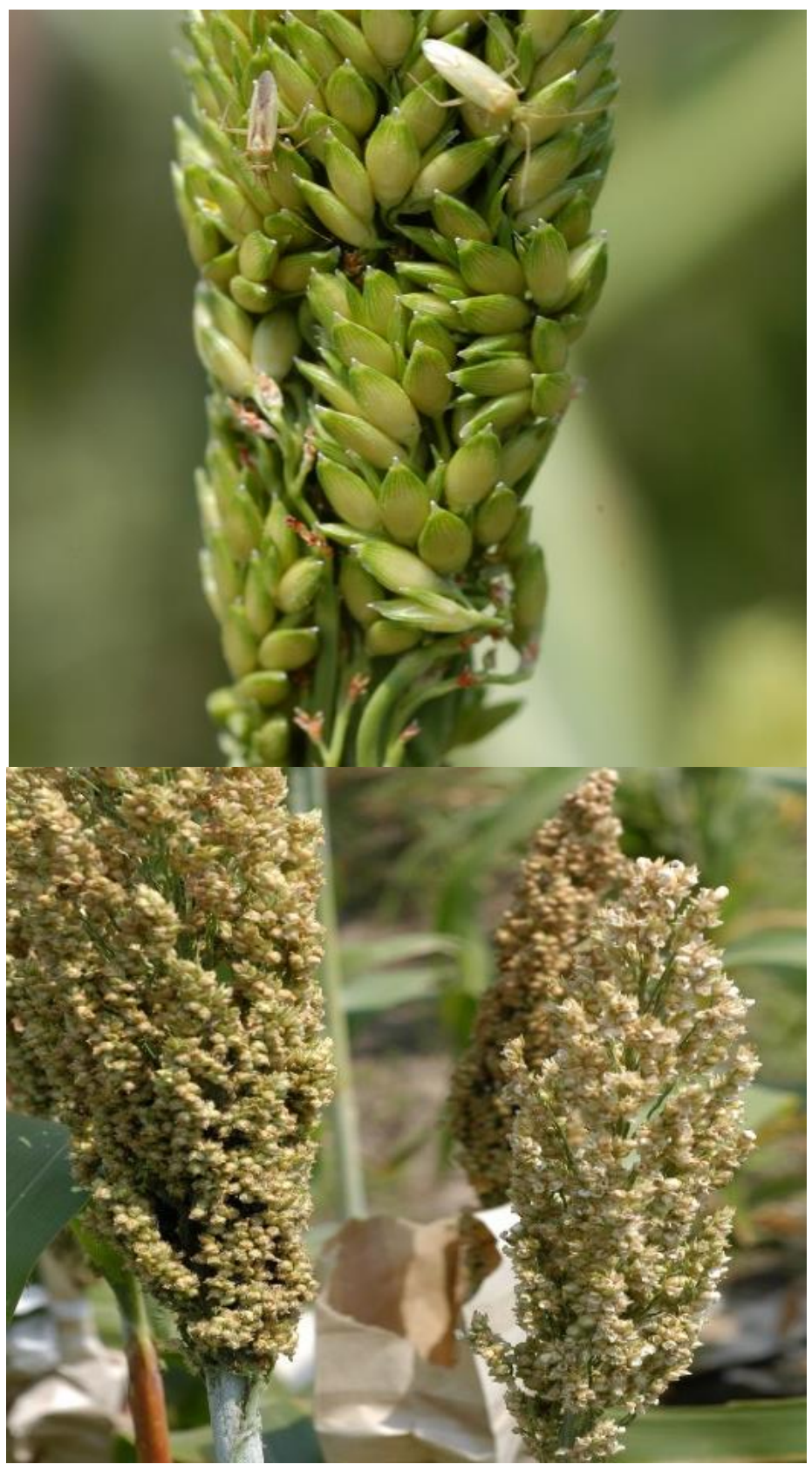

Source: @ICRISAT HC Sharma

6. Chinch bug, *Blissus leucopterus* (Blissidae: Hemiptera)

Pest description

- Adults are small, black bugs about 1/8 inch long with white wings folded over the back.
- Two small, dark, triangular markings appear near the mid-portion of the wings.

Damage symptoms

- Bugs attack any part of the vegetative phase of sorghum.
- Heavy infestation sometimes results in the death of young plants.
- Even if plants are not killed, the growth may be stunted, and grain and fodder yield is reduced.

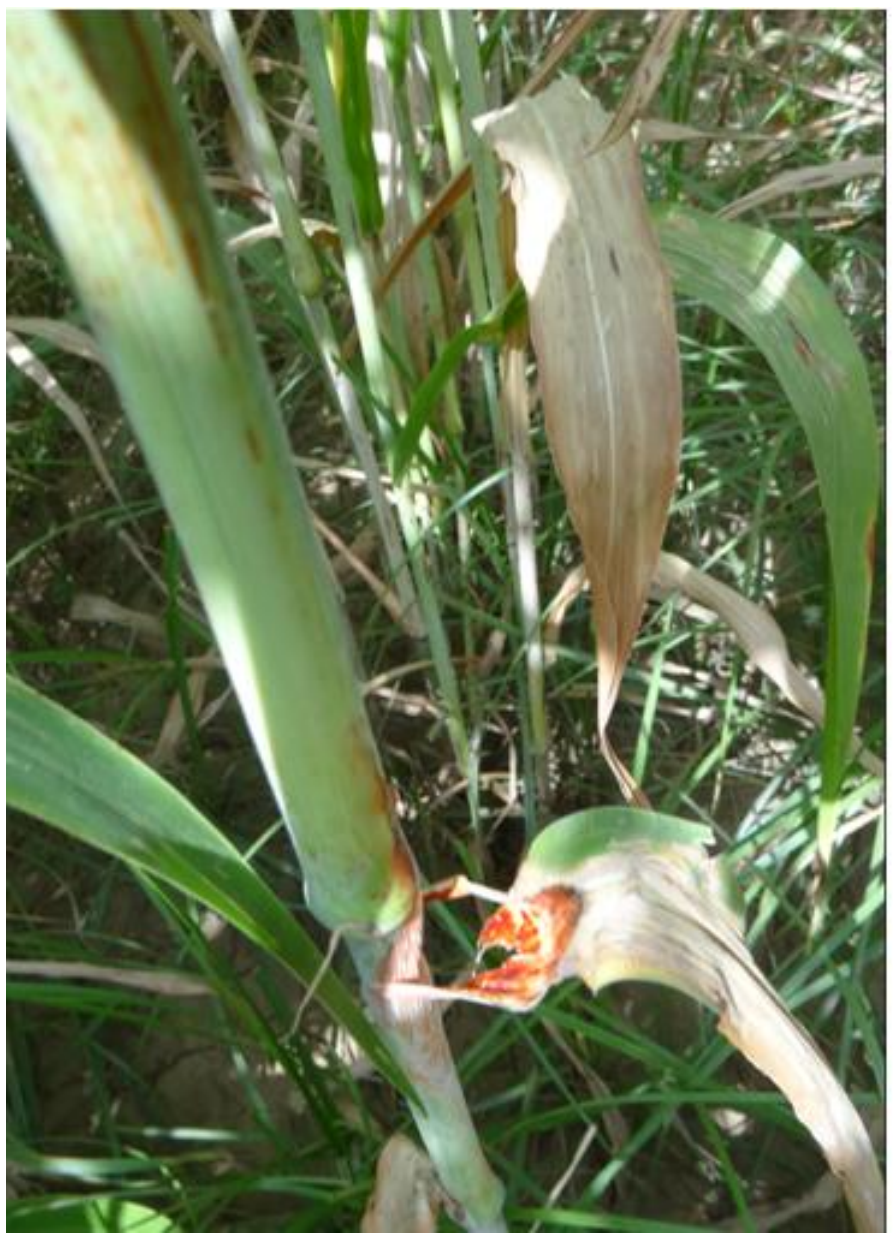

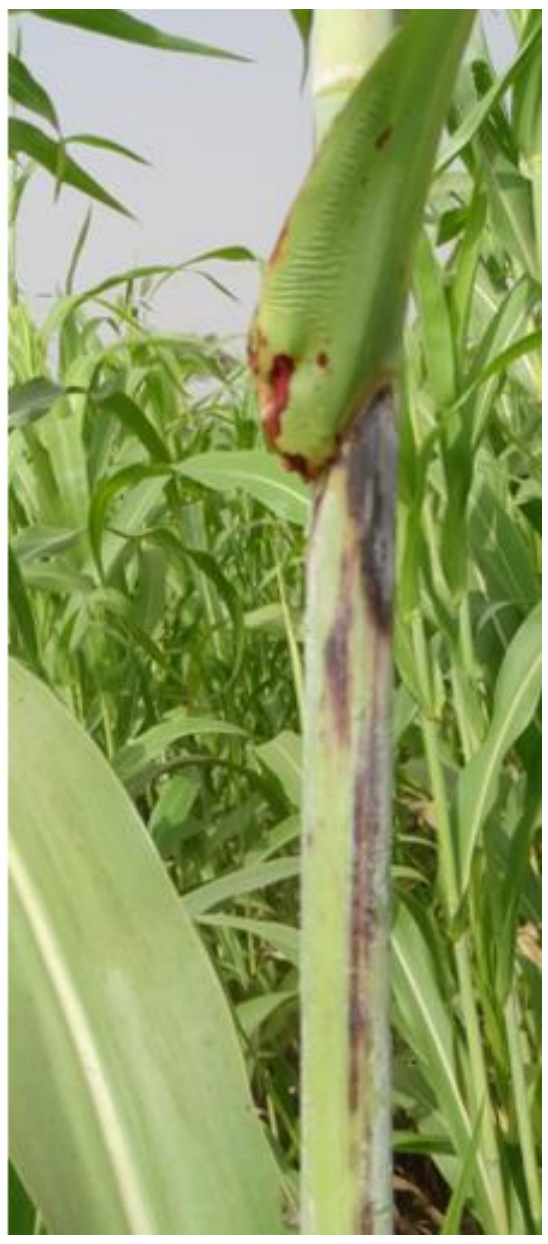

Source: FAO-KSA Survey Report

Management

- Seed treatment with acetamiprid 70 WDG @ 5 g/kg seeds followed by spraying acetamiprid 20% SP @ 0.5 g/l water controls the bugs.

7. Earworm, *Helicoverpa zea*

Pest description

The eggs are pale green when first deposited; larva's head color is usually orange or light brown, patterned like a white net. The forewings of the moths are yellowish brown and hind wings are creamy white basally and blackish.

Damage symptoms

- Larvae chew circular holes in the seed and feed on the starchy content.
- Earheads are partially eaten with a chalky appearance.

Management

- Installation of sex pheromone traps @ 12 traps/ha at 1 ft height above earhead formation for the monitoring of adult male moths.

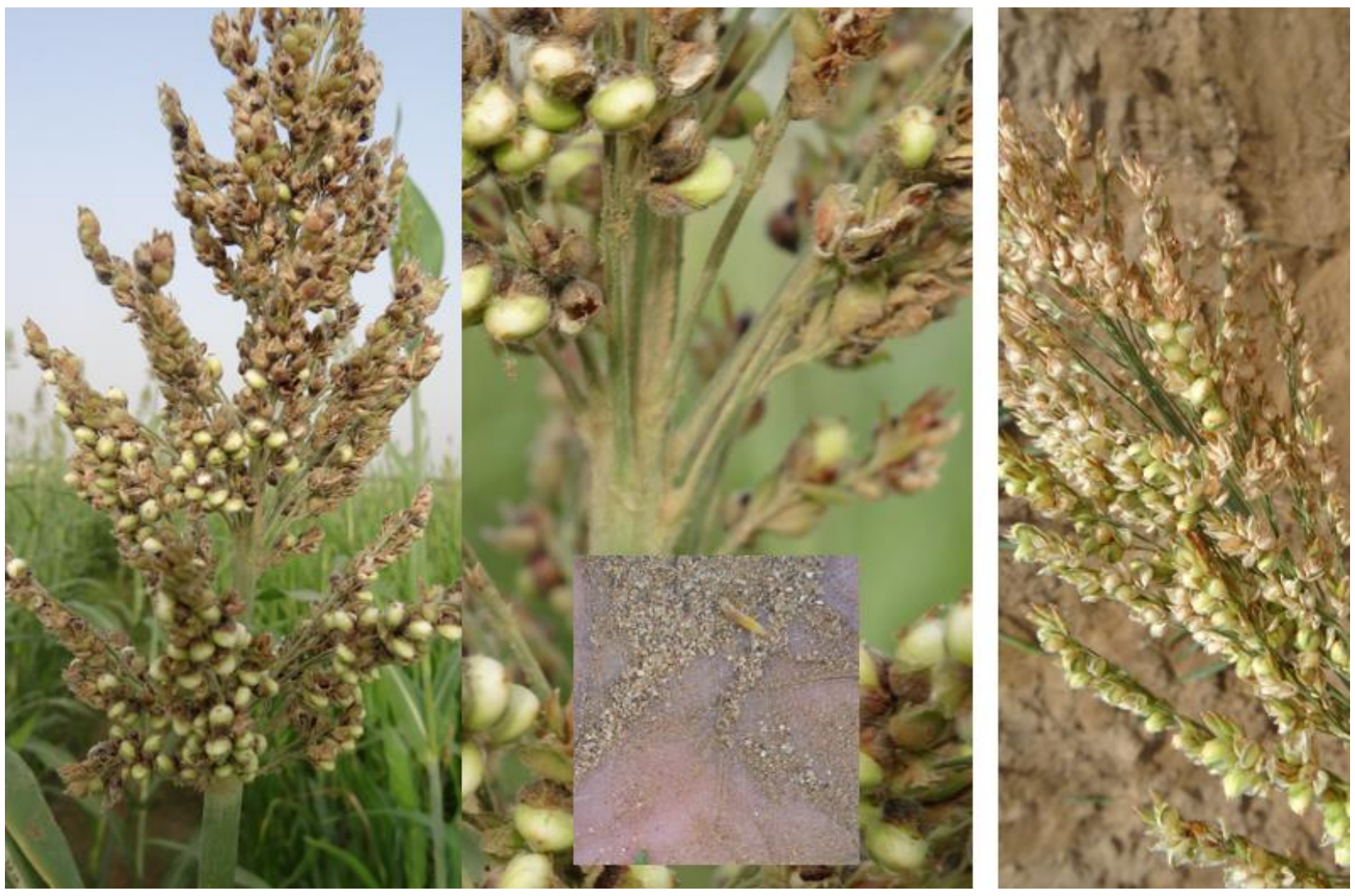

Source: FAO-KSA Survey Report

- Larvae generally appear at earhead stage, hand collection and destruction of this pest is suggested.
- Spinosad 45 SC @ 0.25 ml/l, azadirachtin (5 ml/l), emamectin benzoate 5 SG @ 0.4 g/l water or chlorantraniliprole 20 % SC @ 0.3 ml/l water can be recommended for the management of earworm.
- *Habrobracon hebetor* and *Chelonus blackburni* are the parasitoid and wolf spiders are predators of this pest which can be used as natural enemies.

Suggested readings:

Anitha K, Das IK, Holajjer P, Sivaraj N, Reddy CR, Balijepalli SB. 2020. Sorghum Diseases: Diagnosis and Management. Sorghum in the 21st century: food–fodder–feed–fuel for a rapidly changing world. Pp. 565-619.

Belum VS Reddy, Ravinder Reddy Ch and Ashok Kumar A. 2012. Sweet Sorghum Crop Production and Management Practices. Information Bulletin No. 91. International Crops Research Institute for the Semi-Arid Tropics. Patancheru, 502324, Andhra Pradesh, India. 40 pp. ISBN 978-92- 9066-547-2. Order code: IBE 091.

Nwanze KF, Kokubu H and Teetes GL. 1988. Insect Pests of Sorghum and Their Control. In: 11th International Congress of Plant Protection, 5-9 Oct 1987, Manila, Philippines.

Sharma HC, Leuschner K, Nwanze KF, and Taneja SL. 1992. Techniques to Screen Sorghums for Resistance to Insect Pests. Technical Report. International Crops Research Institute for the Semi-Arid Tropics.

Sharma HC, Singh F and Nwanze KF. 1997. Plant Resistance to Insects in Sorghum. Monograph. International Crops Research Institute for the Semi-Arid Tropics.

Spurlock T, Faske T and Kelley J. Diseases of Grain Sorghum https://www.uaex.uada.edu/publications/pdf/mp297/Diseases%20of%20Grain%20Sorghum-RV2018.pdf

Thakur RP, Reddy BVS and Mathur K. (eds.). 2007. Screening Techniques for Sorghum Diseases. Information Bulletin No. 76. Patancheru 502 324, Andhra Pradesh, India: International Crops Research Institute for the Semi-Arid Tropics. 92 pp. ISBN 978-92-9066-504-5.

Thakur RP, Reddy BVS, Indira S, Rao VP, Navi SS, Yang XB and Ramesh S. 2006. Sorghum Grain Mold. Information Bulletin No. 72. International Crops Research Institute for the Semi-Arid Tropics. Patancheru 502324, Andhra Pradesh, India: 32 pp. ISBN 92-9066-488-6. Order code IBE 072.

TNAU Agritech Portal https://agritech.tnau.ac.in/crop_protection/crop_prot_crop_insectpest%20_cereals_sorghum.html

Acknowledgement: Some of the images and materials presented in this catalogue are taken from ICRISAT's archives and publications and from the IPM Survey Report, FAO-KSA Technical Cooperation Programme, for the project entitled, "Strengthening the Ministry of Environment, Water and Agriculture's (MoEWA) capacity to implement its Sustainable Rural Agricultural Development (SRAD) Programme ".

For further information contact:

Dr Rajan Sharma; Email: rajan.sharma@icrisat.org
Principal Scientist and Cluster Leader - Crop Protection and Seed Health,
Research Program-Accelerated Crop Improvement, ICRISAT, Patancheru - 502 324, India.

Citation: Sharma Rajan, Jagdish Jaba, Ephrem Habyarimana, Shashi Kumar Gupta, Srikanth Rupavatharam, Ashok Kumar Are, Abdullah Alhendi, Alexey Morgounov, Yasaratna Ketipearachchi. 2023. Pests and diseases catalogue – Sorghum. FAO-KSA

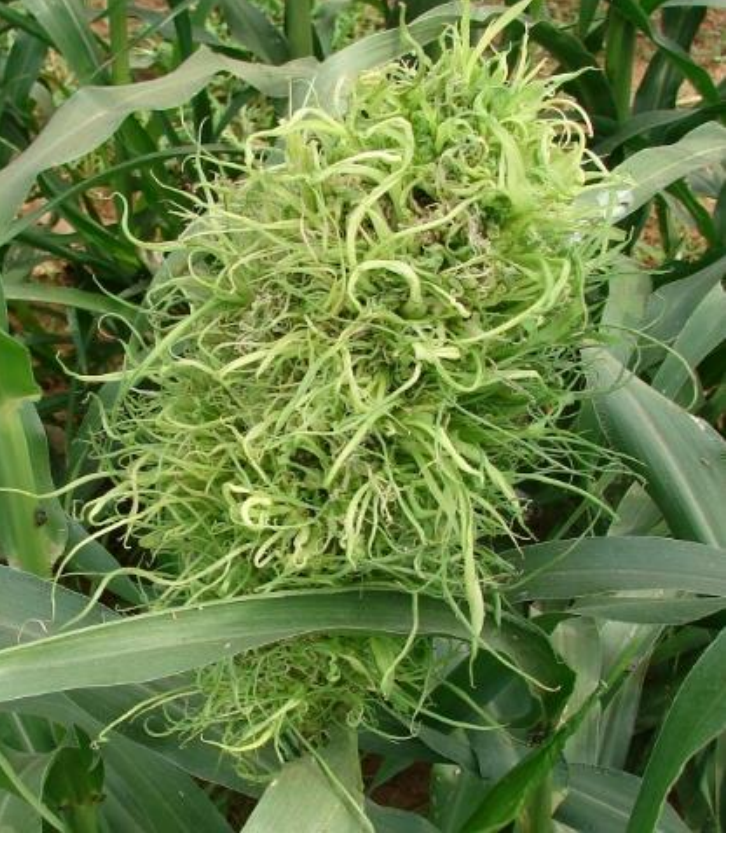

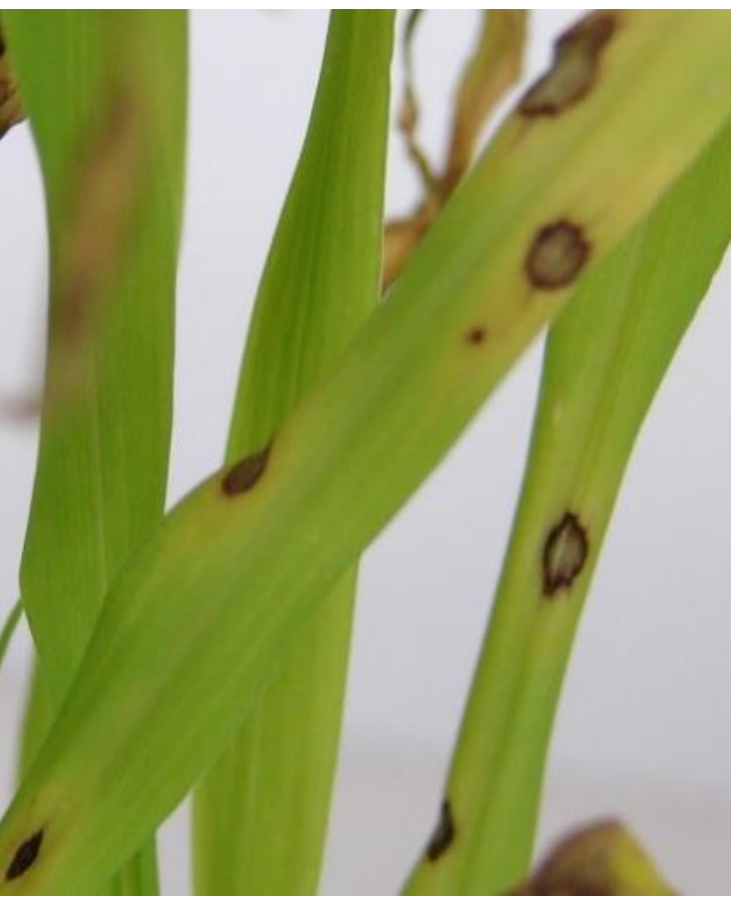

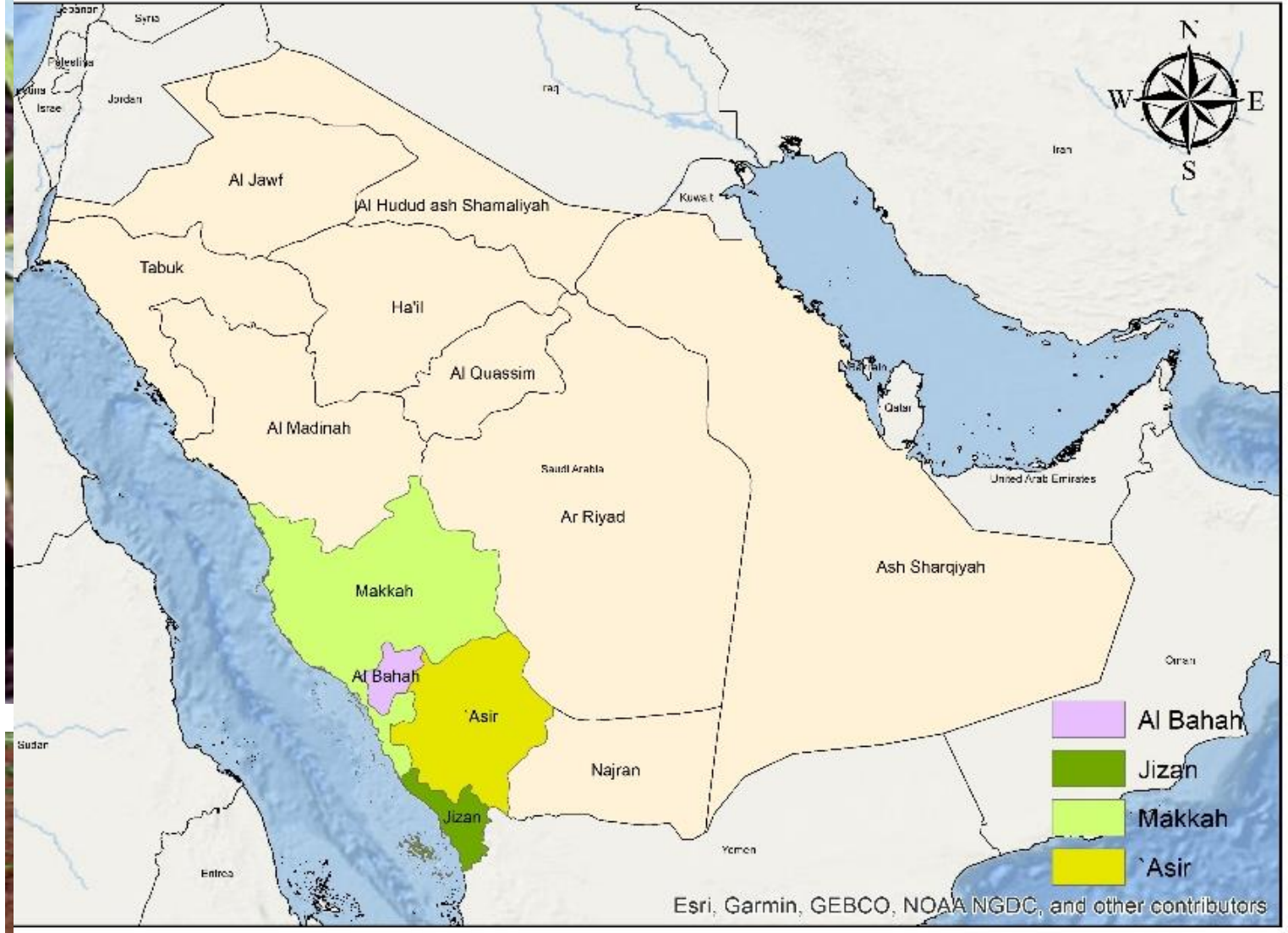

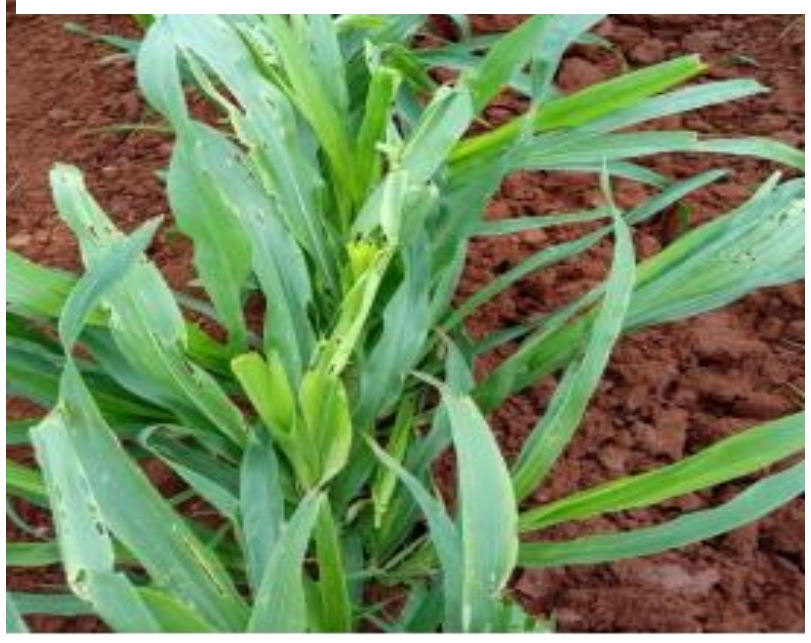

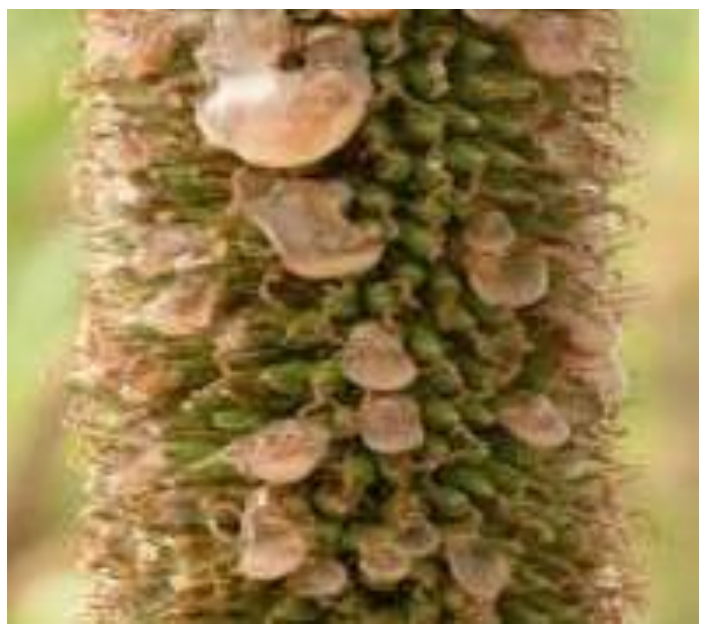

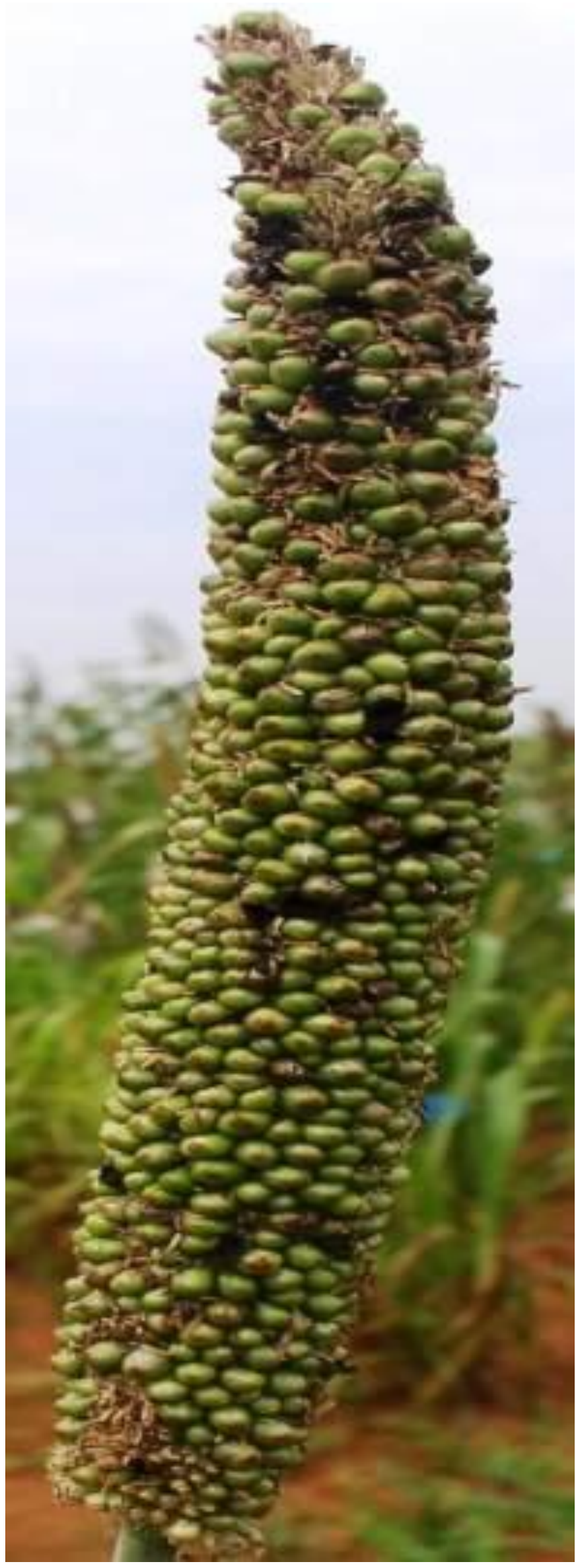

S4. Pests and diseases catalogue – Pearl millet

Pearl millet [*Cenchrus americanus* (L.) Morrone, synonym: *Pennisetum glaucum* (L.) R. Br.,], is a staple food for millions of people living in the semi-arid tropical regions of Africa and Asia. The crop is grown both for grain and fodder. Besides food for human consumption, grains are also used for poultry feed and some industrial uses. Pearl millet is attacked by many diseases caused by oomycetes, fungal, bacterial, and viral pathogens. However, only a few diseases such as downy mildew, blast, rust, ergot, and smut are economically important. Downy mildew is the most destructive and widespread disease of pearl millet. These diseases can be best managed by growing disease resistant varieties and hybrids. Several sources of disease resistance are available at ICRISAT which need to be tested in the Kingdom of Saudi Arabia (KSA).

Worldwide, at least 150 insect species have been recorded as feeding on millets; most of these pests are common to all species of millets. Insect damage in millets has been recorded across all plant growth stages and can occur on foliage, flowers as well as on seeds. Pests and diseases infesting/infecting different plant parts at various plant growth stages result in economic losses due to decreased crop productivity, grain quality, and fodder yield. Pearl millet is grown in the South-West of the Kingdom of Saudi Arabia (KSA) mainly in Al Baha, Aseer, Makkah and Jazan. Caterpillar leaf damage, millet stem borer (*Acigona ignefusalis*), head miner *(Heiliocheilus albipunctella*) and leaf spot diseases have been observed on pearl millet in the Jazan region of the KSA. This digital catalogue provides detailed pest/disease description, pictorial representation of damage symptoms of insect pests and diagnostic signs and symptoms of diseases, and pest/disease management options in pearl millet.

ICRISAT
INTERNATIONAL CROPS RESEARCH INSTITUTE FOR THE SEMI-ARID TROPICS

Food and Agriculture Organization of the United Nations

وزارة البيئة والمياه والزراعة
Ministry of Environment Water & Agriculture
Kingdom of Saudi Arabia المملكة العربية السعودية

Diseases of pearl millet

1. Blast, caused by *Pyricularia grisea* [teleomorph - *Magnaporthe grisea*]

Pathogen/disease description

Pyricularia leaf spot, also known as blast disease, is an important disease in the southern United States and more recently it has emerged as a serious disease of dual purpose (grain and fodder) pearl millet hybrids in India. The disease appears in several countries in WCA such as Senegal, Mali, Burkina Faso, Niger, Nigeria and Chad. It was also observed in the Jazan region of the KSA. Leaf blast has been found to be negatively correlated with green-plot yield, dry matter yield and digestive dry matter thus affecting the productivity and quality of the crop. The pathogen infects more than 50 gramineous hosts including rice, wheat, pearl millet, finger millet, foxtail millet, forage, turf grass and several weed hosts. The pathogen is highly variable but specialized in its host range.

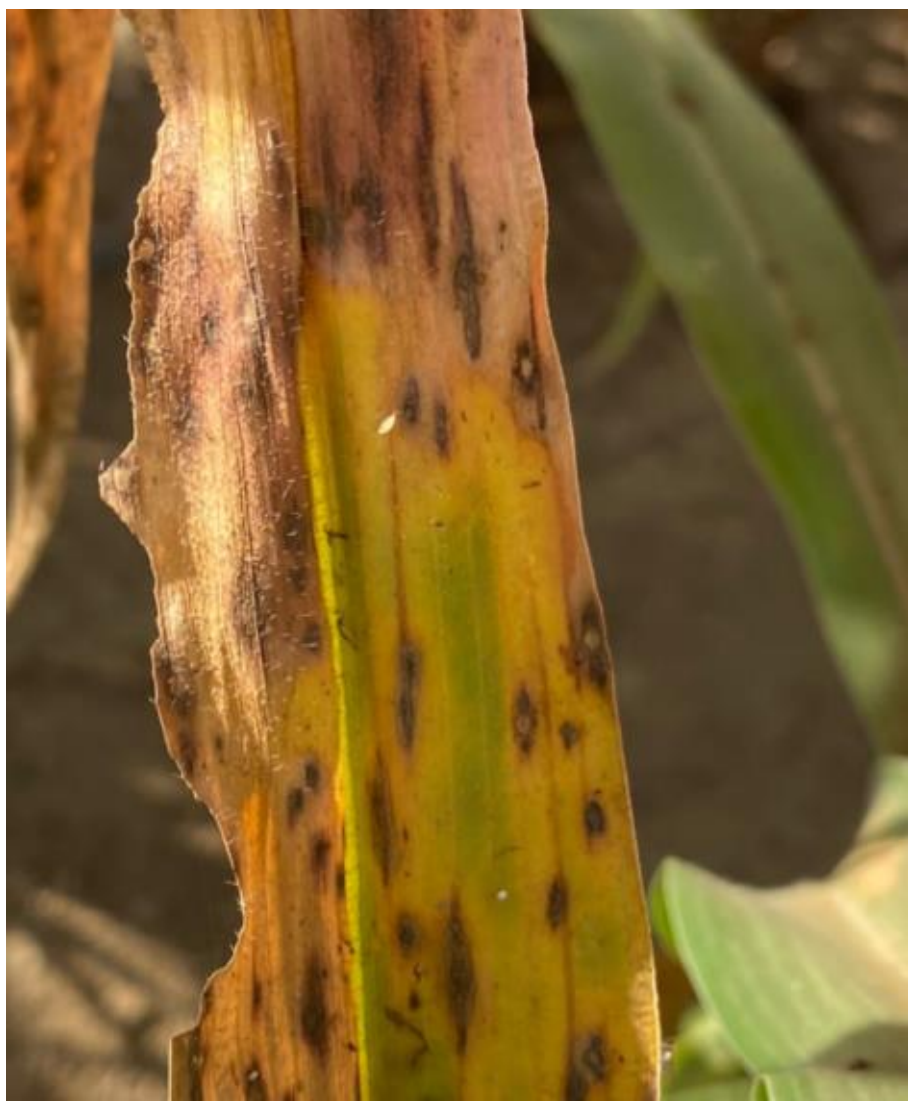
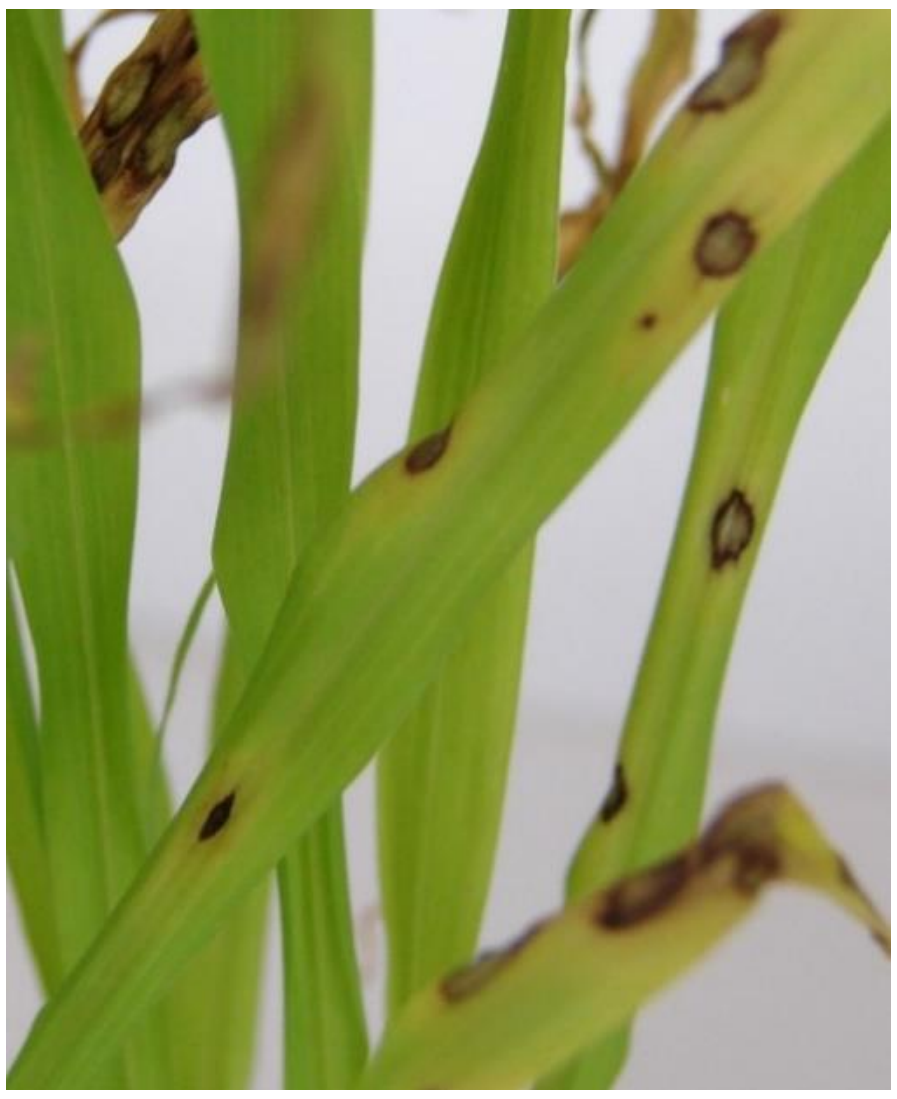
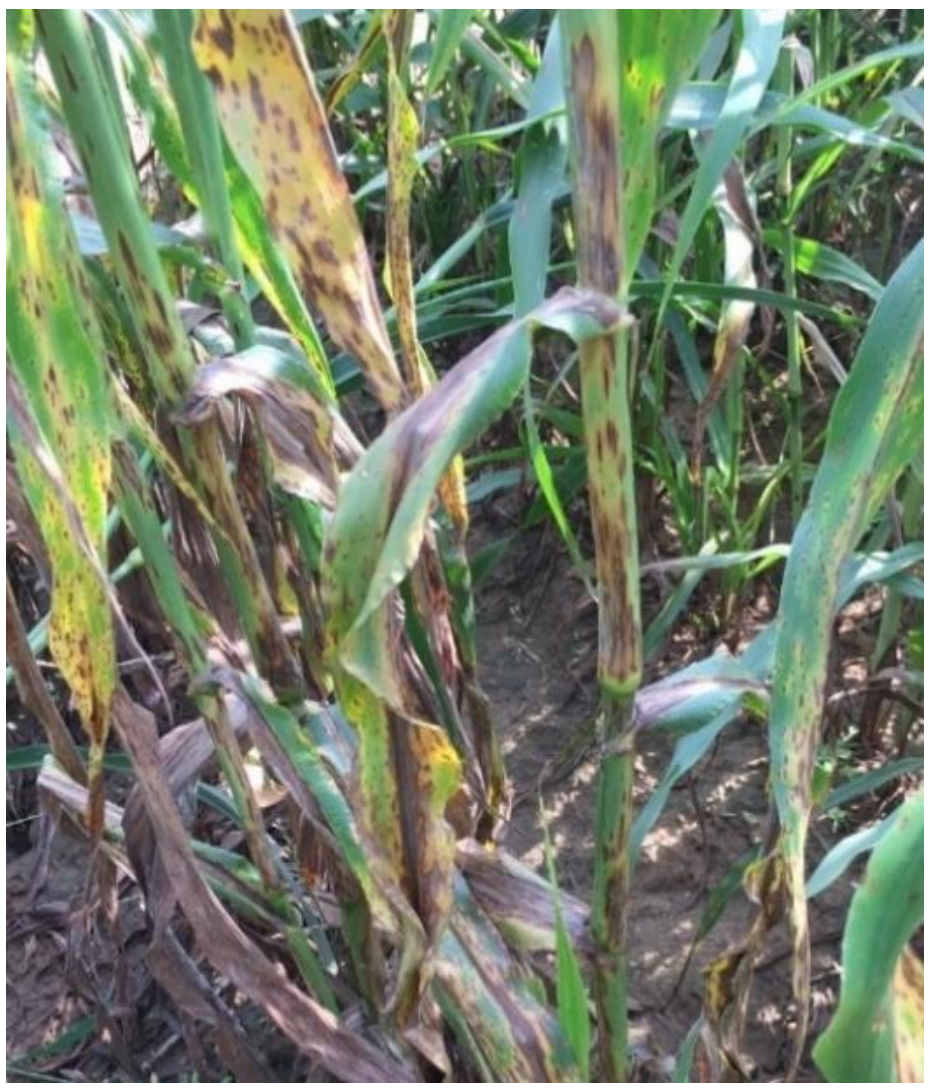

Source: @ICRISAT Rajan Sharma

Disease symptoms

- Grayish, water-soaked lesions on foliage that enlarge and become necrotic.
- The lesion size varies from small, roundish, elliptical, diamond shaped to elongated.
- Lesions grow and coalesce to cover large surface areas and cause necrosis of tissues. Lesions are often surrounded by a chlorotic halo, which turns necrotic, giving the appearance of concentric rings.
- In case of a susceptible cultivar, the entire foliage gives a burnt appearance.
- Severely infected plants produce no grain or few shriveled grains in blasted florets.

Management

- Pathotype-specific resistance sources have been identified at ICRISAT and are being used in breeding programs in India.
- Three sprays of tebuconazole 50% + trifloxystrobin 25% WG @ 0.4 g/l or propiconazole 25% EC @ 1 ml/l water have been found effective in controlling blast.
- Some species of *Pseudomonas*, *Streptomyces* and *Bacillus* have been reported for their antagonistic action against *M. grisea*.
- Integrated approach to manage pearl millet blast includes removal of infected crop residues and weed hosts, use of resistant cultivars and foliar sprays of tebuconazole 50% + trifloxystrobin 25% WG @ 0.4 g/l or propiconazole 25% EC @ 1 ml/l water.

2. Rust, caused by *Puccinia substriata* var. *penicillariae*

Pathogen/disease description

Rust on pearl millet has been reported from many countries of Asia and Africa, and also from the US and Brazil. Substantial losses in grain yield and fodder quality may occur when infection occurs before flowering. Rust is of greater importance on multicut forage hybrids where even low severity can result in substantial losses of digestible dry matter yield. The disease initiates from the infection by urediniospores available from the off season or volunteer pearl millet plants or by the aeciospores from *Solanum* species. Clear, cool nights (<20°C) and dew formation favor infection and rust development.

Disease symptoms

- The rust pathogen is heteroecious and requires two unrelated hosts to complete its life cycle. Pearl millet, on which uredial and telial stages are produced, is called the 'primary host' and brinjal (egg plant – *Solanum melongena* L.) on which spermatia and aeciospores are produced, are the 'alternate host'.
- Infected leaves initially show pinhead chlorotic flecks, which later turn into reddish-orange, round to elliptical pustules on both surfaces. Individual pustules (uredinia) are small and erumpent, and as the disease progresses, they coalesce to occupy larger leaf surface. These pustules contain numerous urediniospores that become airborne as pustules burst.
- As the pustules age, teliospores also appear, although with low frequency.

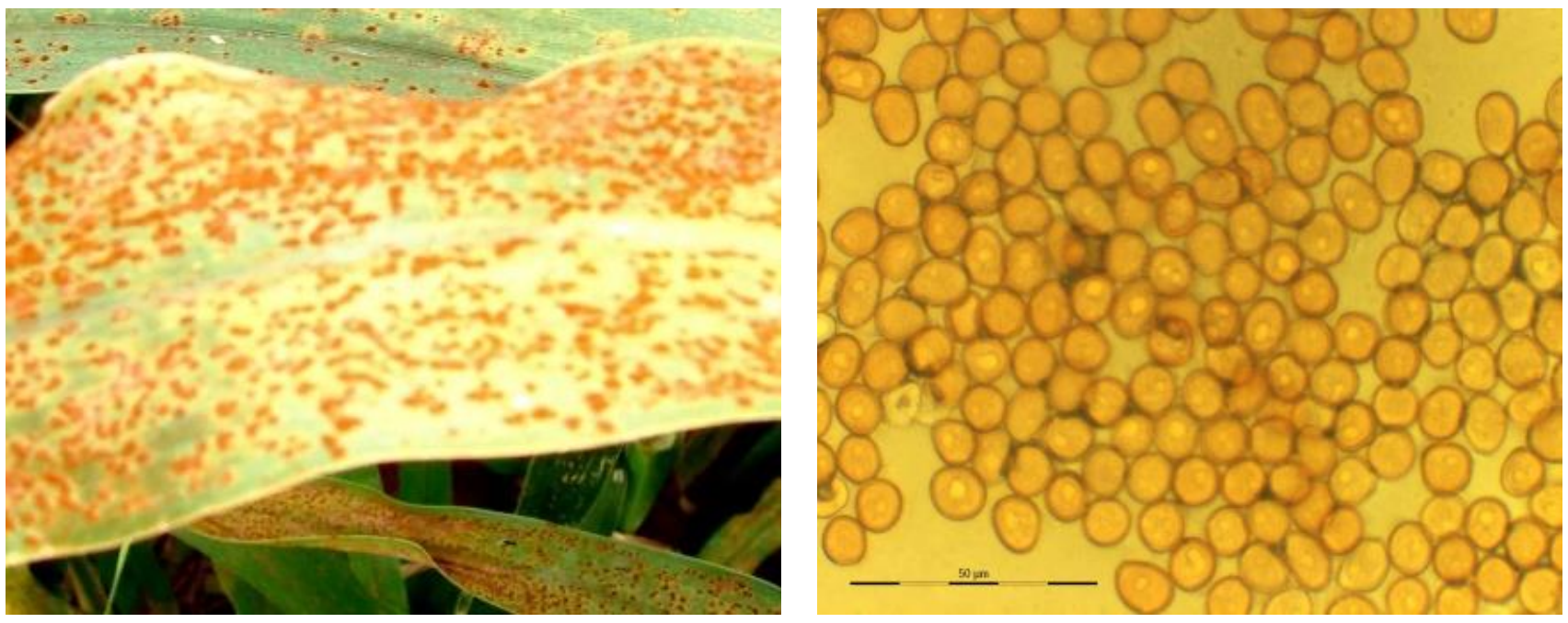

Uredinial pustules and urediniospores

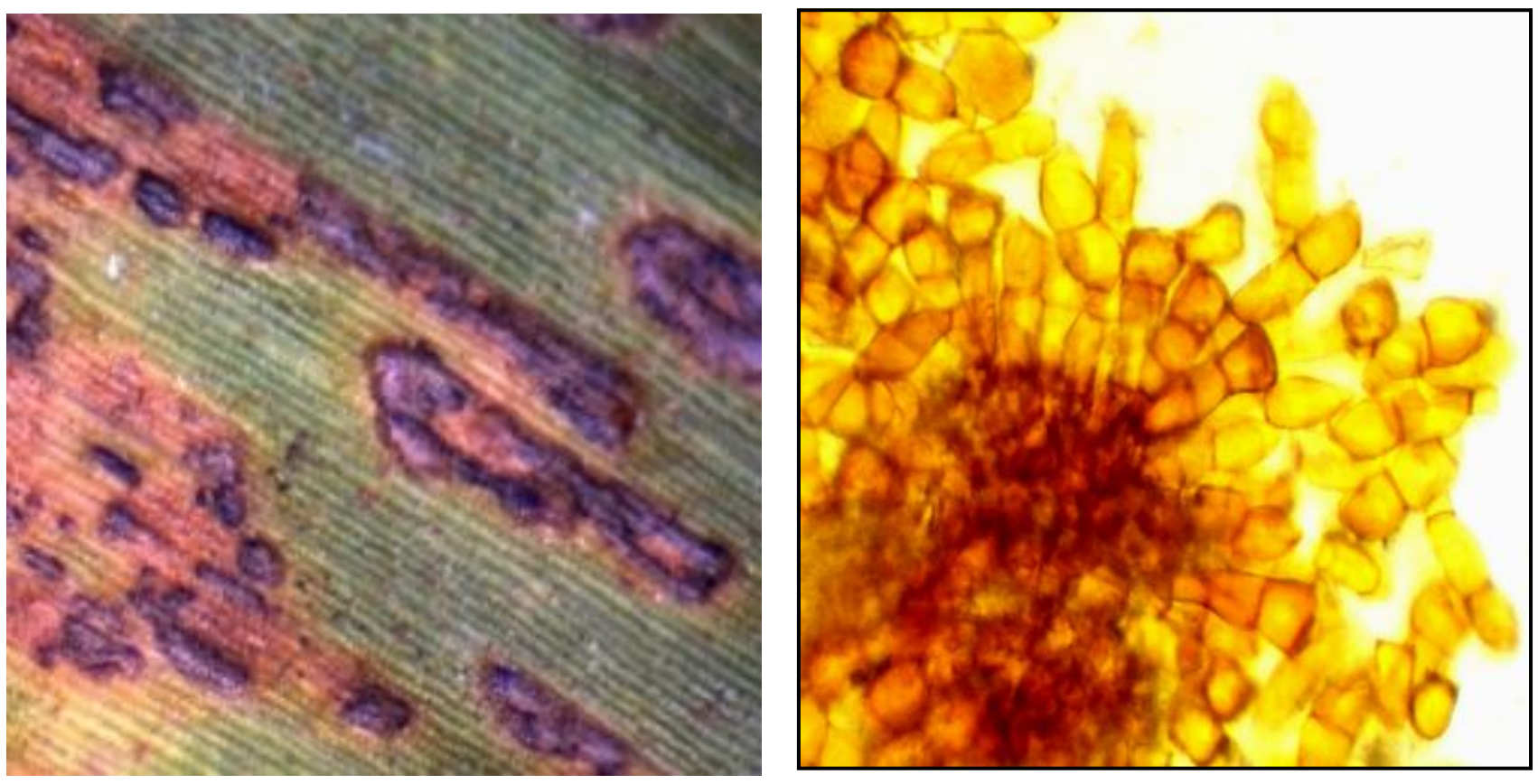

Telial pustules and teliospores

Source: @ICRISAT Information Bulletin No. 89

Management

- A large number of germplasm accessions were evaluated, and many resistant lines have been identified at ICRISAT.
- Spraying the crop with propiconazole 25% EC @ 0.1% have been found effective in controlling rust.
- Avoid the use of overhead irrigation; do not plant pearl millet near eggplant.

3. Downy Mildew, caused by *Sclerospora graminicola*

Pathogen/disease description

Downy mildew is a highly destructive and widespread disease of pearl millet in Asia and Africa. It causes about 20-40% grain yield losses annually worldwide and the losses could be much higher when a susceptible cultivar is repeatedly grown in the same field. Genetically uniform single-cross F_1 hybrids are more susceptible than heterogeneous open-pollinated varieties. The pathogen is highly variable and reproduces both sexually (oospores) as well as asexually (sporangia-zoospores).

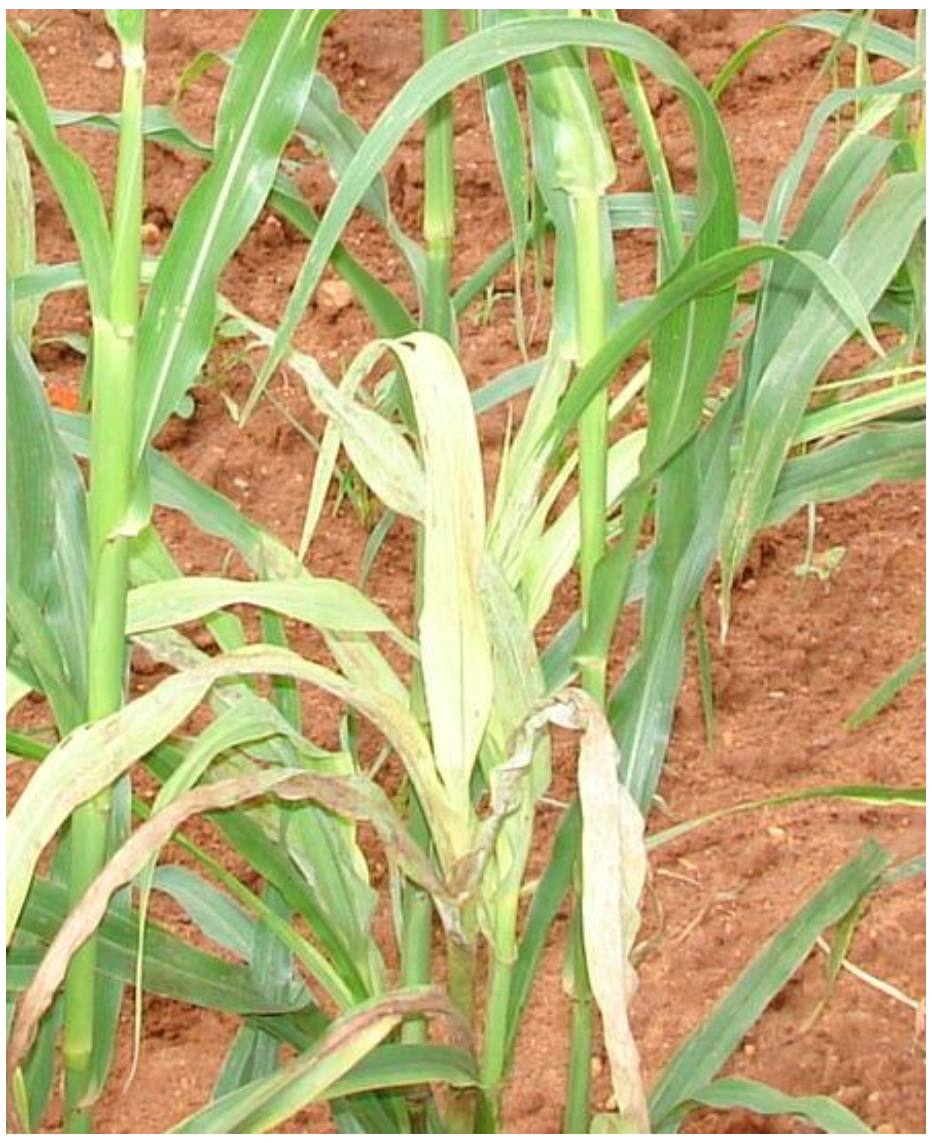
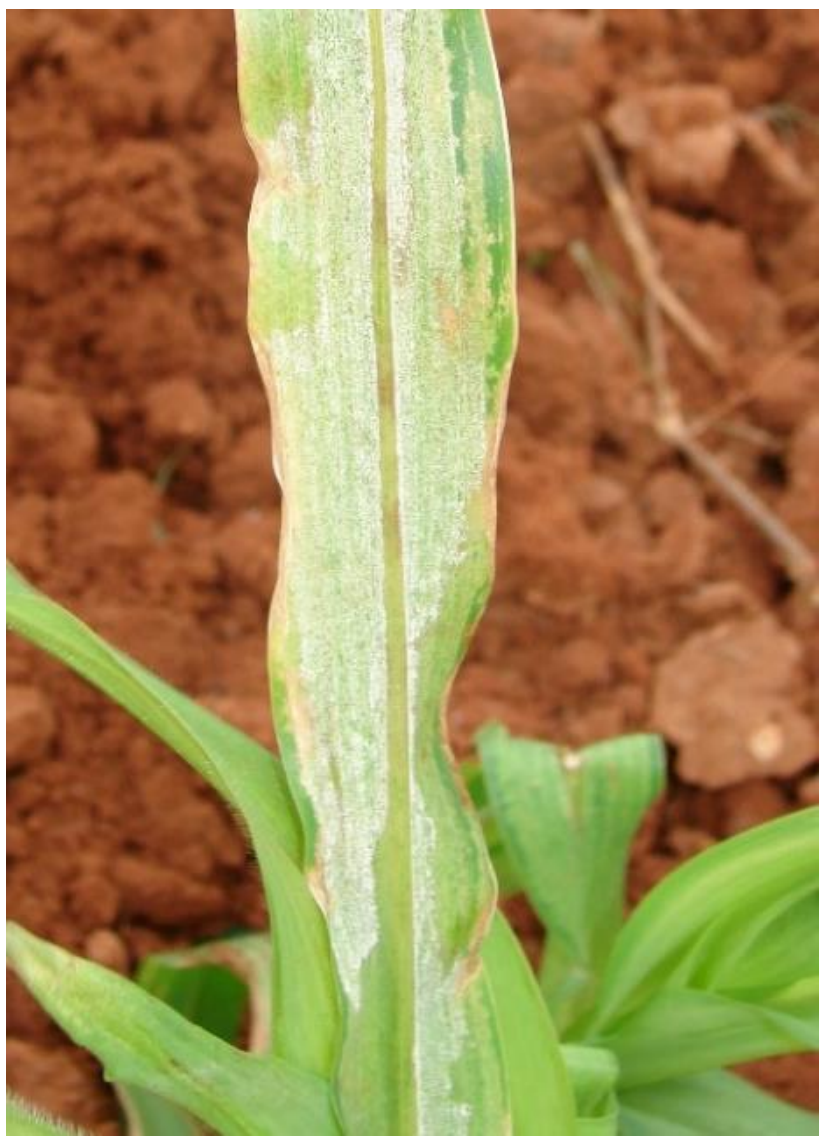
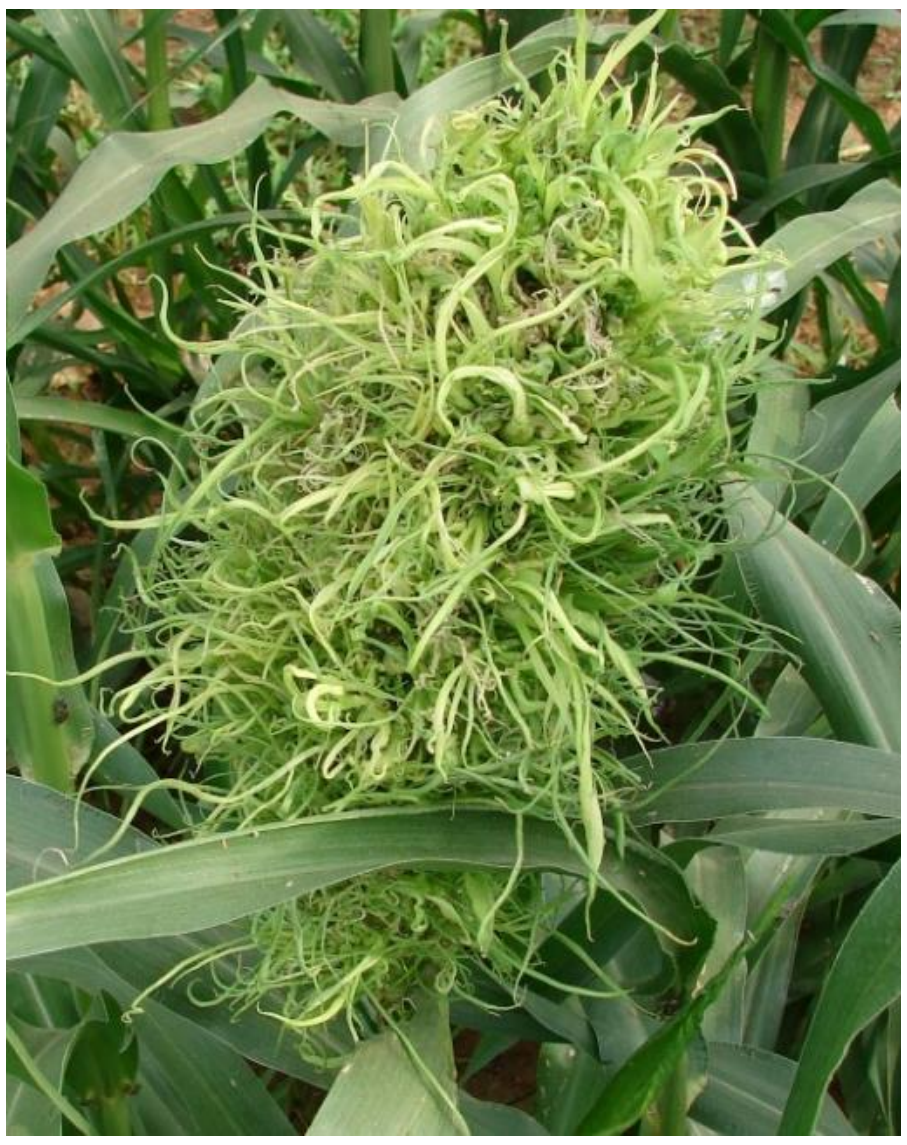

Source: @ICRISAT Rajan Sharma

Disease symptoms

- Primary infection occurs at the seedling stage from the infection caused by soil-borne oospores. Systemic symptoms generally appear as chlorosis on the second leaf, and on all the subsequent leaves. Half-leaf symptoms are also observed.
- Whitish growth of the pathogen in the form of sporangiophores and sporangia appear more on the abaxial leaf surface. These spores get blown off by wind and rain splash and cause secondary infection in the field.
- Green ear symptoms are the appearance of green panicles due to transformation of floral parts into leafy structures.

Management

- The disease can be successfully managed through host plant resistance; several resistance sources were identified at ICRISAT and systematically used in the breeding programs.
- Removal and complete destruction of infected plants from the field; downy mildew-infected plant debris should be burnt, or the field should be ploughed deeply to bury the debris.
- Seed treatment with metalaxyl at 2 g a.i./kg seed controls the disease for about the first 35 days after sowing.
- Metalaxyl seed treatment, deep ploughing and roguing of diseased plants, and the use of resistant cultivars can be combined to develop an effective disease management strategy for pearl millet downy mildew.

4. Smut, caused by *Moesziomyces penicillariae* (Syn. *Tolyposporium penicillariae*)

Pathogen/disease description

Smut is an important disease of pearl millet in India, western Africa and USA. Although, present in almost all countries where pearl millet is grown, no epidemics have been reported so far, and the extent of losses caused by the disease is quite variable. It is a panicle disease that is more severe in CMS-based single-cross hybrids than in open-pollinated varieties.

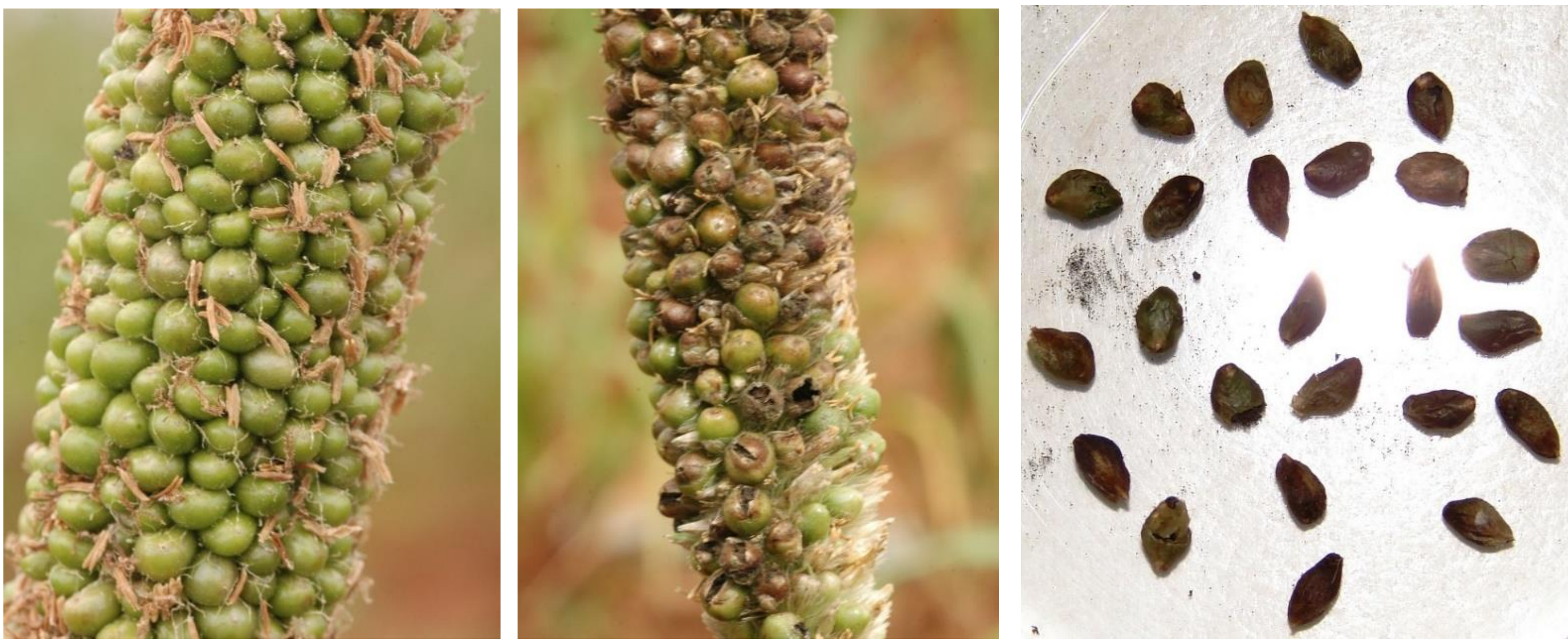

Source: @ICRISAT RP Thakur

Disease symptoms

- Smut symptoms appear on the panicle as green, shining smut sori in place of grains two weeks after infection; the sori mature within the next two weeks. Matured sori turn brown and rupture to release dark-brown to black sporeballs of numerous teleutospores.
- Smut infection begins from soil- and seed-contaminated (not internally seed borne) inocula. Teleutospores from the previously infected florets are left in the soil and seed get contaminated at threshing.
- Because of the long latent period secondary spread within a crop is limited, but the late tillers and late planted crops in the adjacent fields may get infected.

Management

- Roguing out of infected ears, use of clean seed, field sanitation, crop rotation and hot weather deep ploughing are some of the cultural practices that reduce primary inoculum of the fungus.
- A number of lines were evaluated at hot spots in India and countries in WCA and several lines with stable resistance to smut were identified. Some of the resistant lines are - SSC FS 252-S-4 (ICML 5), ICI 7517-S-1 (ICML 6), EBS 46-1-2-S-2 (ICML 7), EB 112-1-S-1-1 (ICML 8), NEP 588-5690-S-8-4 (ICML-9) and P 489-S-3 (ICML 10) that can be tested.
- Integrated approach to manage pearl millet smut includes removal of smutted ears, use of clean seeds, deep summer ploughing, crop rotation, and use of smut resistant cultivars.

Insect pests of pearl millet

1. Millet head miner (MHM), *Heliocheilus albipunctella* (Noctuidae: Lepidoptera)

Pest description

The millet head miner is a monophagous species that feeds only on millet. Female moths lay eggs on the developing millet heads and full-grown caterpillars are pink in color. The mature caterpillars drop to the ground, where they burrow into the soil to pupate, usually close to the host plant.

Damage symptoms

- Young larvae perforate millet glumes and eat the flowers, whereas mature larvae cut the floral peduncles, thereby preventing grain formation or causing mature grains to spill, with grain yield losses ranging from 15 to 85%.
- As the larvae chew between the rachis and flowers, they lift the destroyed flowers or developing grains, leaving a characteristic spiral pattern on the millet head.

Management

- ICMP 177001, ICMP 177002, ICMV 177003, ICMV IS 90311, LCIC 9702, Souna 3, ICMV IS 94206 and PE 08043 have moderate resistance to MHM.
- Biological control with the parasitoid wasp *Habrobracon* (= *Bracon*) *hebetor* Say (Hymenoptera: Braconidae) resulted in parasitism of 80% of the MHM larvae and 30% increase in grain yield thus can be used as natural enemy.
- Insecticides, chlorantraniliprole 20% SC @ 0.3 ml/l water, and insect growth regulators (diflubenzuron @ 1 g/l, lufenuron @ 1.5 ml/l) can significantly reduce the pest incidence with a single application at 75% flowering or two applications, the first at the beginning of flowering and the second 5–7 days later.

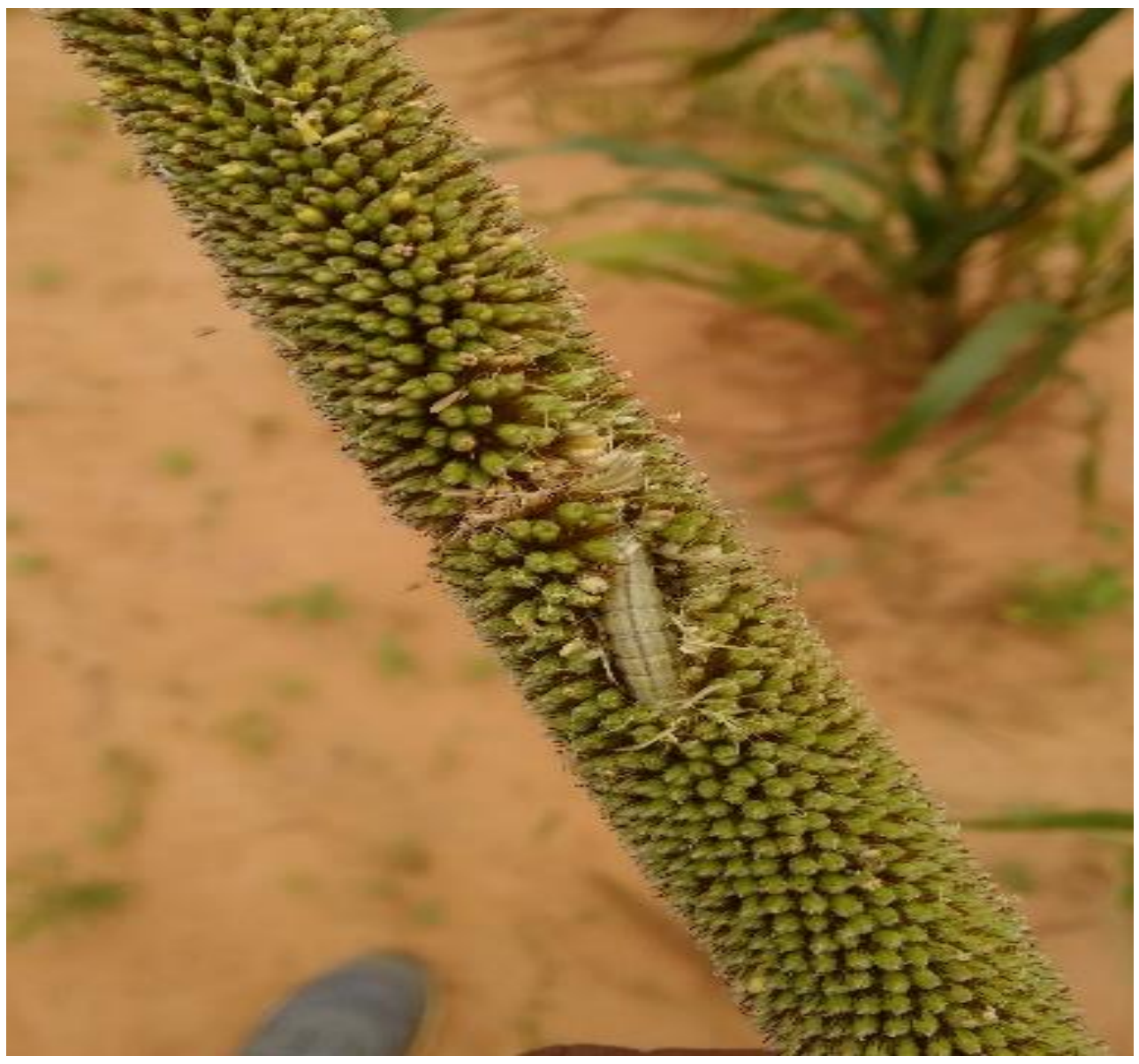

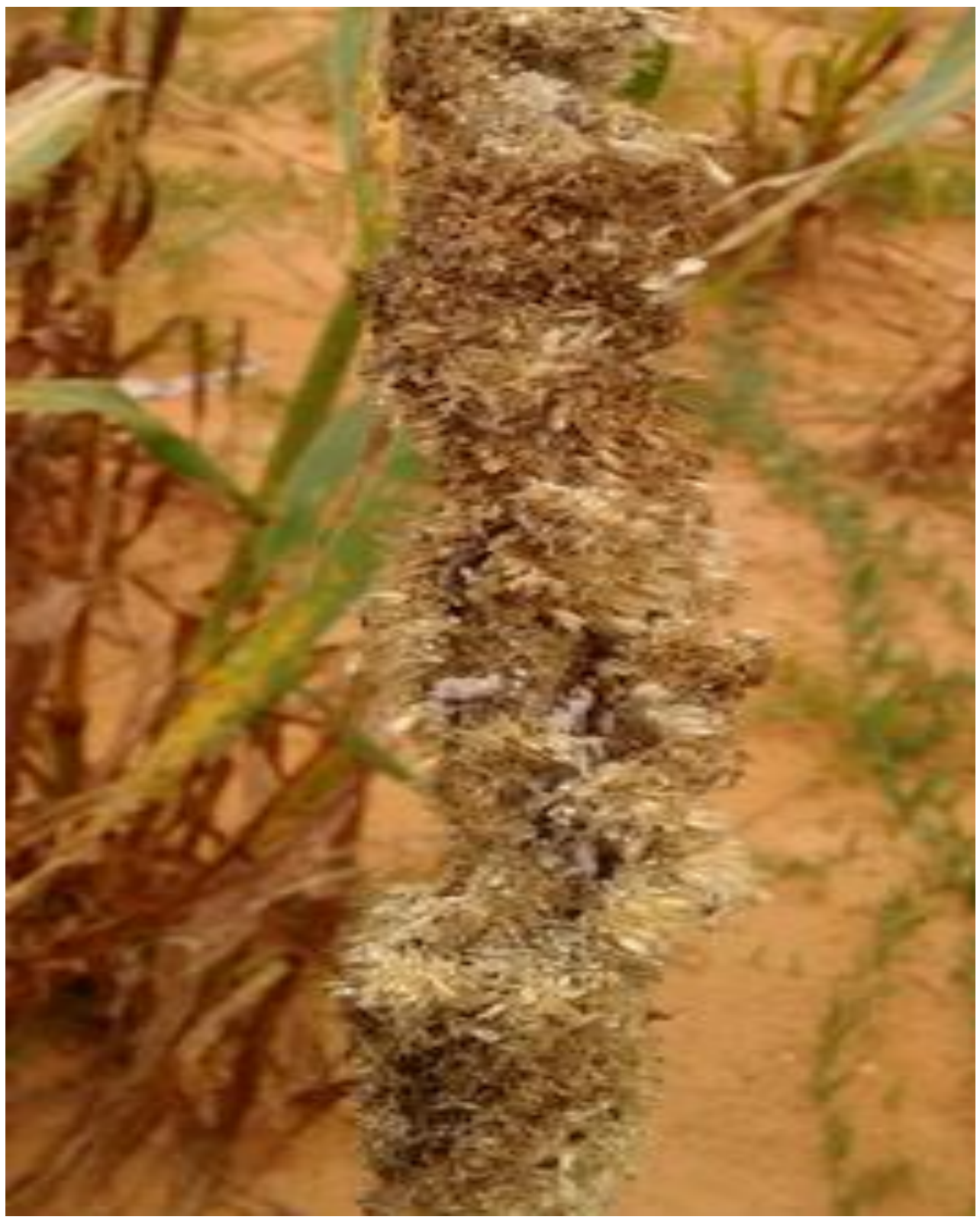

Source: @ICRISAT M Riyazaddin

2. Shoot fly, *Atherigona approximata,* Malloch (Muscidae: Diptera)

Pest description

Eggs are laid generally singly parallel to the midrib on the undersurface of the 3rd to 5th leaf; maggot are typically yellow in color and the adults are dark brown and resemble housefly.

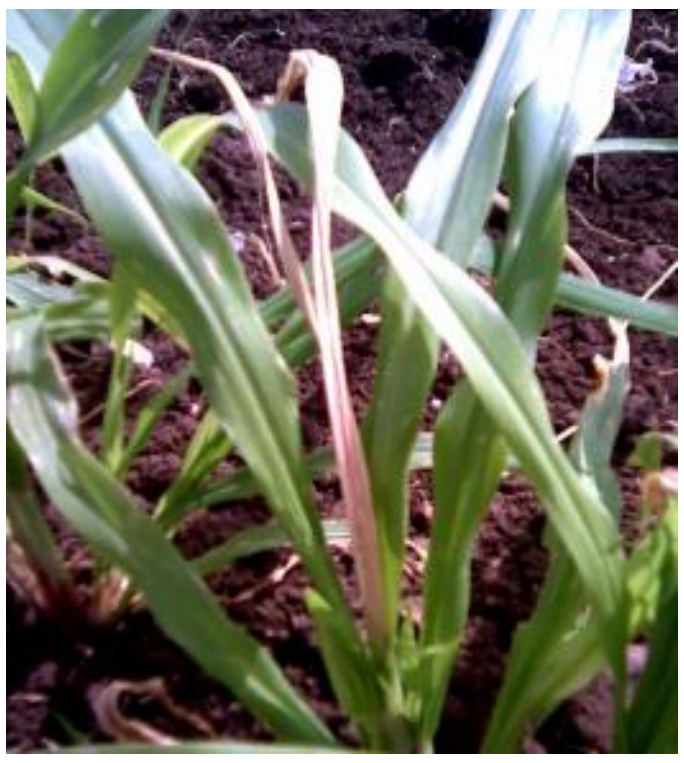

Damage symptoms

- Damage by larvae at the seedling stage (5 to 30 days after seedling emergence) leads to the typical dead heart symptoms.
- The larva cut the growing point. As a result, the central leaf dries up forming a dead heart, which can be pulled out easily.
- Late infestations may also damage the panicle in the formative stage, resulting in rotting or drying up and the earhead appear like cat's tail.

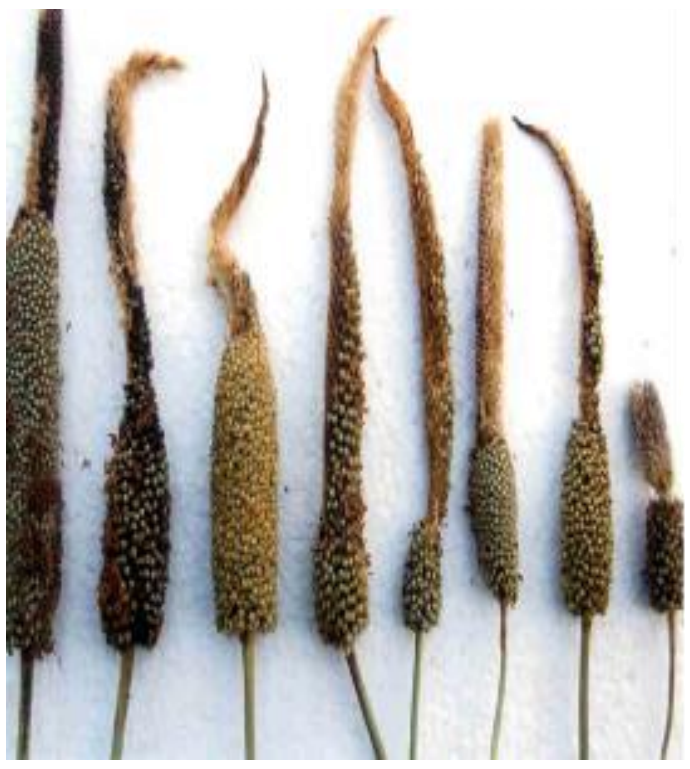

Source: @AICRP Pearl millet
Rajkumar Juneja

Management

- Use of resistant/tolerant varieties/hybrids.
- Use a higher seed rate and remove the shoot fly-damaged seedlings at the time of thinning.
- Set up hanging type plastic fishmeal trap @ 12/ha till the crop is 30 days old.
- Seed treatment with thiamethoxam 25 WG @ 5 g/kg of seeds.
- Under late plantings, shoot fly can be controlled with the application of imidacloprid 2 GR @ 450 g/ha at the time of planting in the seed furrows. Alternatively, shoot fly damage can be minimized by spraying chlorantraniliprole 20% SC @ 0.3 ml/l water at 7 and 14 days after the emergence of seedlings.

3. Stem borers, *Coniesta ignefusalis* and *Sesamia* spp. (Crambidae: Lepidoptera)

Pest description

- ***C. ignefusalis***: A full-grown larva measures 15–18 mm, has a whitish gray body with black oval spots on each segment.
- ***Sesamia* spp.**: Pinkish brown with dark head.

Damage Symptoms

- The pink larvae of *Sesamia* are gregarious in nature, congregate inside the leaf whorls and feed on the central leaves causing typical 'pin hole' symptom.
- The central shoot turns brownish and dries out that leads to the typical dead heart symptoms, although the lower leaves remain green and healthy. Bored holes are plugged with excreta.
- Early season attack by *C. ignefusalis* results in the destruction of the growing points of millet plants, resulting in premature plant death called 'dead heart'. Attack on older plants by tunnelling larvae causes disruption of nutrient flow, stem lodging and poor or no grain formation.

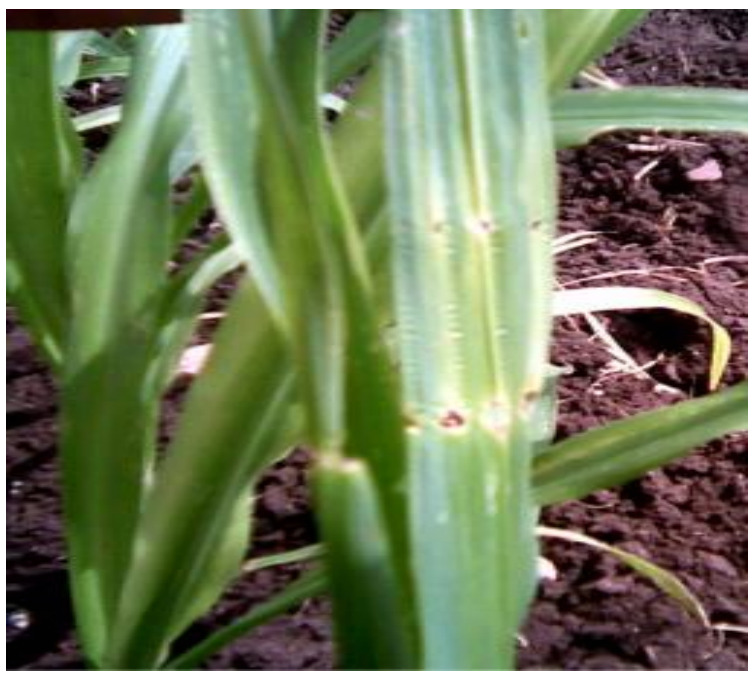

Source: @AICRP Pearl millet
Rajkumar Juneja

Management

- Spray chlorantraniliprole 20% SC @ 0.4 ml/l; or thiamethoxam 25% WG @ 0.5 g/l at 3rd and 18th day after panicle emergence.
- *Tetrastichus atriclavus* is a larval parasitoid of stem borer that can be used as natural enemy.
- Set up light traps till mid night to monitor, attract and kill adults of stem borer, grain midge and earhead caterpillars.

4. Fall armyworm, *Spodoptera frugiperda* (Noctuidae: Lepidoptera)

Pest description

Larvae with 4 black spots arranged (in square shape) on 8th and 9th abdominal segment and inverted “Y” shaped mark on head. Male adult moth’s forewings are brown with triangular white spots whereas, females’ forewings are grayish brown.

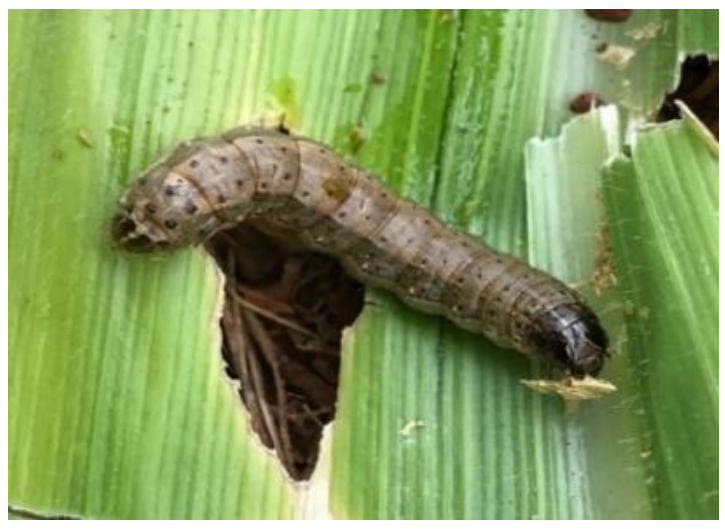

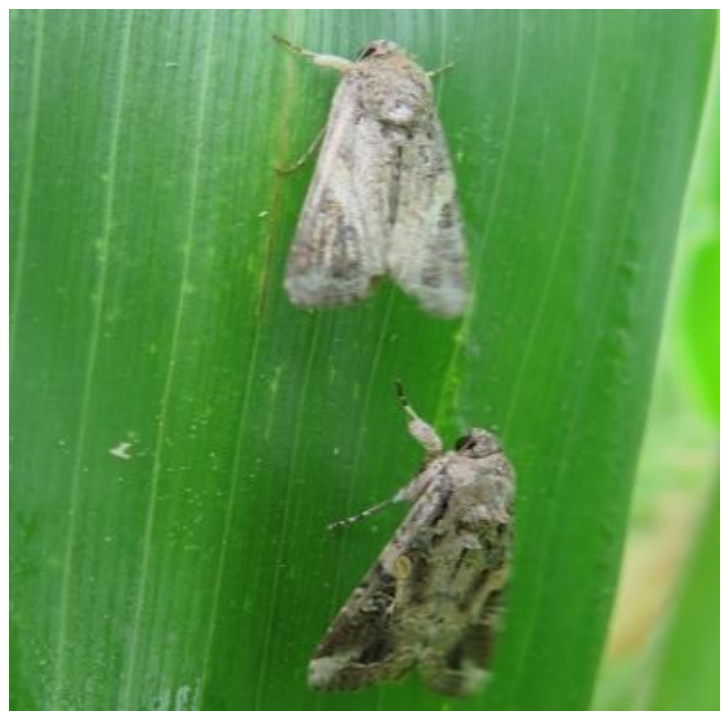

Damage symptoms

- Scrapping of leaf surface by young larvae.
- The damage by late instars (4th instar) results in extensive defoliation of leaves and the presence of large amounts of fecal pellets in whorls.

Management

- Critical period to protect the crop is up to 50-60 days after seedling emergence.
- Seed treatment with cyantraniliprole 10% OD @ 4 ml/kg seed is reported to offer protection for 2-3 weeks after germination.
- Scouting and assessment of the damage and existence of natural enemies.
- For mass trapping, use pheromone traps, 25 traps/ha.
- *Telenomus remus* and *Trichogramma* spp. are egg parasitoids, and ladybird beetles and ground beetle feed on young caterpillars. These can be used as natural enemies of the pest.
- Spinosad 45 SC @ 0.25 ml/l or azadirachtin (5 ml/l) can be used.
- Other effective pesticides are: Emamectin benzoate 5 SG @ 0.4 g/l; chlorantraniliprole 20% SC @ 0.3 ml/l water.

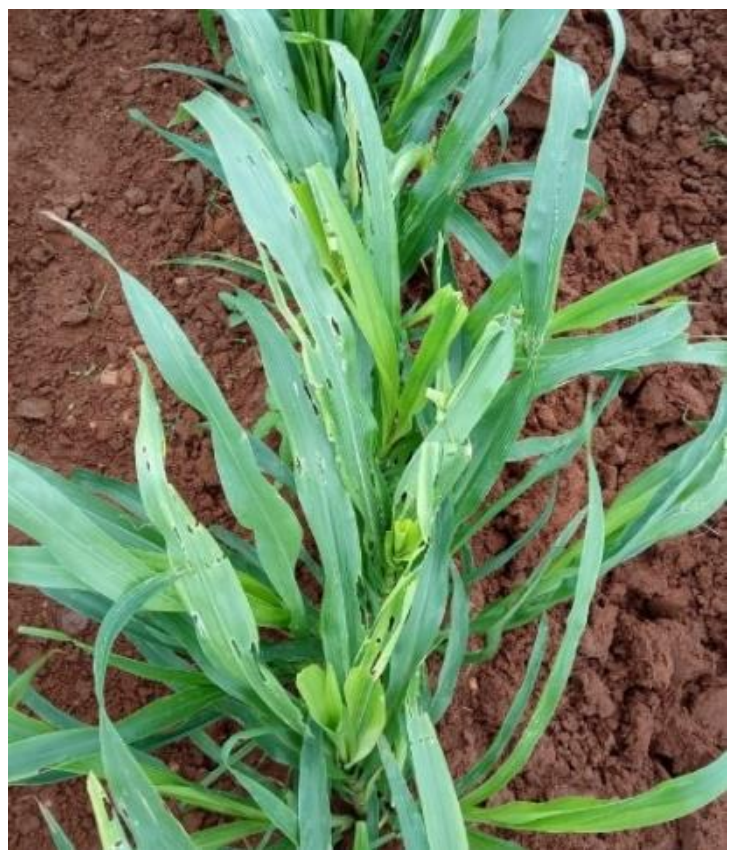

Source: @ICRISAT Jagdish Jaba

5. Earhead worm, *Helicoverpa armigera* (Noctuidae: Lepidoptera)

Pest description

Most of the larvae are dark greenish brown, but they can also be pink, cream or almost black colored.

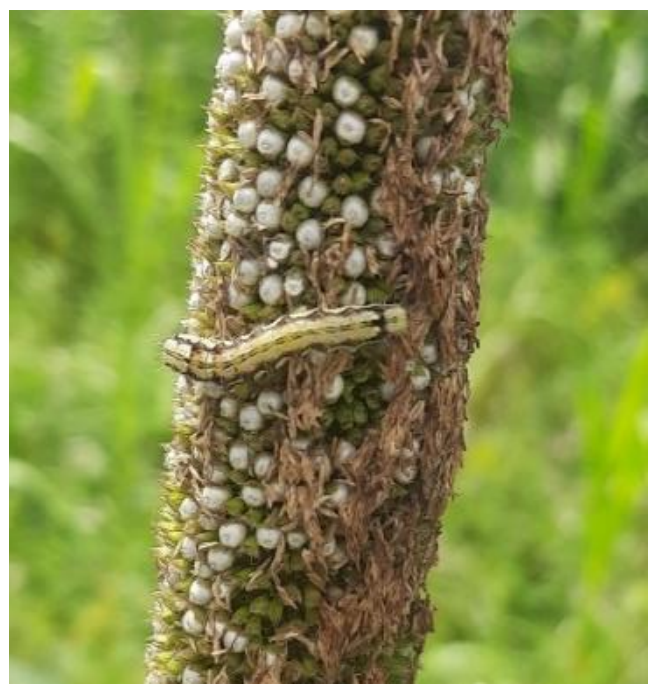

Damage Symptoms

- Egg laying by female moth is specifically done on the pearl millet earheads at earhead emergence stage.
- Freshly hatched larvae feed on stigma which ultimately leads to poor grain setting and it is sometimes misguided with sterility.

Source: @ICRISAT Jagdish Jaba

Management

- Installation of sex pheromone traps @ 12 traps/ha at 1 ft height above earhead formation for monitoring of adult male moths.
- *H. armigera* larvae generally appear at earhead stage, hand collection and destruction of this pest is suggested.
- Spinosad @ 0.25 ml/l is an effective ovicidal; Azadirachtin (5 ml/l) an ovipositional deterrent can also be used.
- *Habrobracon hebetor* and *Chelonus blackburni* are parasitoids and wolf spiders are predators which can be used as natural enemies.
- Emamectin benzoate 5 SG @ 0.4 g/l water and chlorantraniliprole 20% SC @ 0.3 ml/l are effective pesticides.

Suggested readings:

Das IK, Nagaraja A and Tonapi VA. 2016. Diseases of millets-a ready reckoner. https://www.millets.res.in/books/DISEASES_OF_MILLETS.pdf

Sharma HC and Davies, JC. 1988. Insect and other animal pests of millets. Documentation. International Crops Research Institute for the Semi-Arid Tropics, Patancheru, Andhra Pradesh, India.

Gahukar RT and Reddy Gadi VP. 2019. Management of Economically Important Insect Pests of Millet. Journal of Integrated Pest Management, 10 (1), 8. https://doi.org/10.1093/jipm/pmz026

Juneja RP, Parmar GM, Chaudhari RJ and Mungra KD. 2022. Monitoring of major insect pests, correlation and yield loss in pearl millet. Insect Environment 25 (2): 202-206.

Kalaisekar A, Padmaja PG, Bhagwat VR and Patil JV. 2017. Insect pests of millets: systematics, bionomics and management. 1st ed. Elsevier, New York, NY.

Sharma R, Karthikeyan G, Pushpavathi B, Gate VL and Raj C. 2016. Diseases of pearl millet. Pp.235-294. In: Diseases of field crops and their management (Dubey SC, Aggarwal R, Patro TSSK and Sharma P (Eds.). Today & Tomorrow's Printers and Publishers, New Delhi.

Singh SD, King SB and Weraer J. 1993. Downy mildew disease of pearl millet. Information Bulletin no. 37. (In En. Summaries in Fr, Es.) Patancheru, A.P. 502 324, India: International Crops Research Institute for the Semi-Arid Tropics. 36 pp. ISBN 92-9066-257-3. Order code: IBE 037.

Thakur RP and King SB. 1988. Smut disease of pearl millet. Information Bulletin no.25. Patancheru, A.P. 502 324, India: International Crops Research Institute for the Semi-Arid Tropics.

Thakur RP, Sharma Rajan and Rao VP. 2011. Screening Techniques for Pearl Millet Diseases. Information Bulletin No. 89. Patancheru 502 324, Andhra Pradesh, India: International Crops Research Institute for the Semi-Arid Tropics. 56 pp.

Williams RJ, Frederiksen RA and Girard JC. 1978. Sorghum and pearl millet disease identification handbook. https://oar.icrisat.org/1213/1/RA_00423.pdf

Acknowledgement: Some of the images and materials presented in this catalogue are taken from ICRISAT's archives and publications and from the IPM Survey Report, FAO-KSA Technical Cooperation Programme, for the project entitled, "Strengthening the Ministry of Environment, Water and Agriculture's (MoEWA) capacity to implement its Sustainable Rural Agricultural Development (SRAD) Programme ".

For further information contact:

Dr Rajan Sharma; Email: rajan.sharma@icrisat.org
Principal Scientist and Cluster Leader - Crop Protection and Seed Health,
Research Program-Accelerated Crop Improvement, ICRISAT, Patancheru - 502 324, India.

Citation: Sharma Rajan, Jagdish Jaba, Shashi Kumar Gupta, Srikanth Rupavatharam, Ephrem Habyarimana, Ashok Kumar Are, Abdullah Alhendi, Alexey Morgounov, Yasaratna Ketipearachchi. 2023. Pests and diseases catalogue – Pearl millet. FAO-KSA
